# Anti-Infective-Associated AKI: A Narrative Review of the Epidemiology, Mechanisms, Risk Factors, Biomarkers, Clinical Course, Monitoring, Prevention, and Therapeutic Strategies

**DOI:** 10.3390/antibiotics14111138

**Published:** 2025-11-10

**Authors:** Iman Karimzadeh, Sandra L. Kane-Gill, Binglei Ma

**Affiliations:** 1 Department of Clinical Pharmacy, School of Pharmacy, Shiraz University of Medical Sciences, Shiraz 71468-64685, Iran; karimzadee@sums.ac.ir; 2 Department of Pharmacy and Therapeutics, School of Pharmacy, University of Pittsburgh, Pittsburgh, PA 15261, USA; kane-gill@pitt.edu; 3 Department of Pharmacy, University of Pittsburgh Medical Center, Pittsburgh, PA 15260, USA; 4 Center for Critical Care Nephrology, Department of Critical Care Medicine, University of Pittsburgh School of Medicine, Pittsburgh, PA 15213, USA; 5 State Key Laboratory of Oncology in South China, Collaborative Innovation Center of Cancer Medicine, Sun Yat-sen University Cancer Center, Guangzhou 510060, China; 6 Department of Urology, Sun Yat-sen University Cancer Center, Guangzhou 510060, China

**Keywords:** nephrotoxicity, acute kidney injury, anti-infective agents, drug induced, physiopathology, prevention, treatment

## Abstract

Acute kidney injury (AKI) occurs commonly in hospitalized patients, especially patients in intensive care units (ICUs). Medications are among the major causative factors of AKI. This narrative review addressed and updated different aspects of anti-infective-associated AKI, including amphotericin B, cidofovir, foscarnet, polymyxins, vancomycin, and aminoglycosides. There is no standard definition or operational criteria to describe anti-infective-associated AKI. Characteristically, it usually occurs during the first two weeks of treatment and is typically dose dependent. Functional resolution occurs, but kidney injury can affect renal functional reserve and increase susceptibility to future AKI events. A variety of pathophysiological mechanisms impacting glomerular, tubular, and interstitial components of the kidney are usually responsible for the development of AKI from anti-infective medications. Oxidative stress and inflammation play a pivotal role in the pathogenesis of antibiotic-related AKI. Numerous patient-related, medication-related, and co-administered-related scenarios have been demonstrated as risk factors for anti-infective-induced AKI. Apart from traditional indexes of kidney function (serum creatinine and urine output), novel biomarkers of kidney function (e.g., serum cystatin C) and damage (e.g., urinary kidney-injury molecule-1 and neutrophil gelatinase-associated lipocalin) have been noticed in recent clinical studies with promising findings. The efficiency of preventive strategies against anti-infective-associated AKI in most cases appears to be variable, relative, and modest. Close and regular monitoring of kidney function parameters is crucial during treatment with nephrotoxic antibiotics. Currently, there is no definitive treatment modalities for the management of AKI with anti-infectives. Therefore, supportive care is the mainstay of treatment.

## 1. Introduction

Acute kidney injury (AKI) transpires commonly in hospitalized patients, especially those in intensive care units (ICUs). It occurs in about 3–7% of hospitalized and 25–60% of ICU patients [1]. Patients with AKI may exhibit a 4- to 10-fold increase in mortality and prolonged hospital stays than those without AKI. In terms of economic burden, the average patient costs of AKI in the ICU are generally double than those of non-AKI patients [2]. About one-fourth of all medications given in hospitals are potentially nephrotoxic [3]. Therefore, drug-induced kidney disease (DIKD) accounts for about 19 to 26% of cases of AKI in hospitalized patients [4]. In ICU patients, following sepsis, hypotension, trauma, and surgery, medications are the third- to fifth-leading cause of AKI [5]. Hospital admission costs of patients who developed DIKD ranged from USD 47,696 to 173,569 [6].

A standardized list of nephrotoxins can enable a healthcare professional to better categorize and prioritize nephrotoxic stewardship in different clinical settings, especially for critically ill patients. Recently, Grey et al. demonstrated that by using an appraised nephrotoxic potential rating system, 20 medications were considered to have probable to probably/definite nephrotoxicity for critically ill patients by both physicians and pharmacists. They included amikacin, carboplatin, cisplatin, diclofenac sodium, ketorolac, tobramycin, amphotericin B, cidofovir, colistin, gentamicin, methotrexate, vancomycin, cyclosporine, indomethacin, naproxen, foscarnet, ibuprofen, ketoprofen, rofecoxib, and tacrolimus [7]. A similar pattern of high-risk nephrotoxic medications in pediatric and non-ICU inpatients has been also reported [8,9].

Although there are several dedicated reviews that consider the nephrotoxicity of prominent medications mentioned above, to our knowledge, there is no published review that has comprehensively discussed different aspects of all these nephrotoxic medications together. The aim of this narrative review is to provide in-depth and updated data about the epidemiology, definition, pathophysiological mechanism(s), risk factors, biomarkers, clinical/paraclinical manifestations, clinical outcome, and preventive as well as therapeutic strategies of AKI caused by well-known nephrotoxic anti-infectives. These include amphotericin B, cidofovir, foscarnet, polymyxins, vancomycin, and aminoglycosides. We selected these anti-infectives as the focus of this review because of their concern for a highly probable association with nephrotoxicity [7,8,9].

A literature review was performed by related search terms from MeSH like “liposomal amphotericin B”, “amphotericin B deoxycholate”, “cidofovir”, “foscarnet”, “polymyxins”, “vancomycin”, “aminoglycosides”, “acute kidney injury”, “physiopathology”, “risk factors”, “epidemiology”, “prevention”, “drug monitoring”, and “therapy”. The major databases used for searching were PubMed, MEDLINE, and the Web of Science. Only English-language articles and clinical investigations were recruited. Besides original research studies, case reports, case series, and review articles were also considered eligible. Despite there being no specific time limitation, we mostly focused on studies published during the recent 10 years.

## 2. Amphotericin B

### 2.1. Introduction

Amphotericin B (AmB) is a polyene macrolide antibiotic that has a broad spectrum of activity against pathogens, causing disseminated, serious, and life-threatening mycotic and parasitic infections [10]. These infections include disseminated candidiasis, blastomycosis, disseminated histoplasmosis, extra cutaneous sporotrichosis, mucormycosis, invasive aspergillosis, leishmaniasis, and naegleriasis [10,11]. According to its high efficiency, low rate of resistance, and also low cost, AmB generally has remained as the first-line antifungal agent for the empiric treatment of invasive candidiasis in critically ill patients [12].

### 2.2. Epidemiology

Despite its clinical effectiveness, AmB is associated with a wide variety of acute and chronic adverse reactions. Among them, nephrotoxicity is generally considered as the most clinically significant one that limits its utility. AmB deoxycholate, also known as conventional AmB, may induce some degree of kidney impairment in up to 80% of cases [13]. Wide variation in the frequency of AmB AKI results from the presence of risk for damage development, especially its cumulative dose [14]. The frequency of urinary potassium wasting/hypokalemia and urinary magnesium wasting /hypomagnesemia with conventional AmB is reported to be 75–90% and 15.3–48.9%, respectively [15]. The incidence of kidney tubular acidosis and nephrogenic diabetes insipidus caused by AmB is unknown. Lipid formulations of AmB, especially liposomal AmB, is substantially less nephrotoxic than conventional AmB [14]. In this regard, a systematic review and meta-analysis of randomized clinical trials (five trials comprising 1233 patients) reported that the incidence of AKI with conventional and liposomal AmB is 32.5% and 14.5%, respectively [16]. The reported rates of hypokalemia and hypomagnesemia with lipid formulations of AmB are 5–26% and 6–20%, respectively [17].

### 2.3. Definition

Different definitions of AmB-associated AKI have been applied in both observational and interventional clinical studies. For example, a serum creatinine level >2.5 mg/dL was previously considered as an arbitrary threshold for AmB-induced AKI [18]; while, a ≥50% decrease in estimated creatinine clearance or ≥100% increase in serum creatinine (doubling of the baseline value) is the more popular definition of AmB-related AKI in the literature [19]. Noting that more recent studies adhere to the 2012 KDIGO definition of AKI for identifying DIKD [20]. According to this definition, an increase in serum creatinine by ≥0.3 mg/dL within 48 h; an increase in serum creatinine to ≥1.5 times baseline within the previous seven days; or urine output less than 0.5 mL/kg/h for at least 6 h is considered as AmB-associated AKI [21].

### 2.4. Pathophysiological Mechanisms

**Figure 1:**
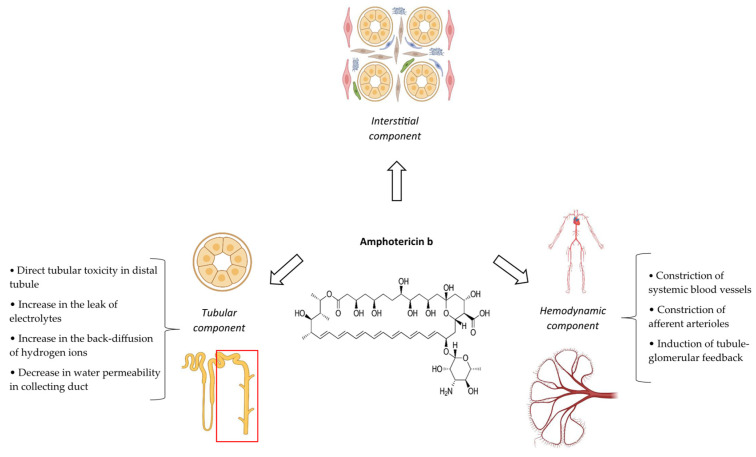
Summary of pathophysiologic mechanisms of amphotericin b nephrotoxicity [13,14,22].

The precise pathophysiology of AmB nephrotoxicity is incompletely understood [22]. Nevertheless, several probable mechanisms have been suggested (Figure 1). In the case of conventional formulation, AmB and its deoxycholate moiety (which is present in the formulation as a detergent) can acutely diminish kidney blood flow and glomerular filtration rate (GFR) via the constriction of systemic blood vessels as well as afferent arterioles [13]. The tubule-glomerular feedback (TGF) system is the other hemodynamic component of AmB nephrotoxicity. Increasing the permeability of the macula densa cells to monovalent ions (such as sodium and chloride) by AmB can inappropriately activate the TGF system, leading to afferent arteriolar vasoconstriction and a fall in the GFR [13].

AmB has also direct detrimental effects on tubules, leading to acute tubular injury (ATI) [14]. Tubular injury of AmB is attributed to the creation of pores secondary to its interaction with ergosterol in the cell membrane that increase cell membrane permeability to cations including sodium and calcium. This causes a cascade of events, culminating in the programmed cell death of tubules [23]. An increase in the permeability of the thick ascending limb of the loop of Henle and the distal tubule by AmB can promote passive distal potassium and magnesium secretion, consequently causing hypokalemia and hypomagnesemia, respectively. However, the serum calcium concentration is usually preserved within the course of AmB nephrotoxicity [13,24]. Acid–base disorders and nephrogenic diabetes insipidus are the other tubular features of AmB nephrotoxicity. In terms of acid–base disorders, AmB can enhance the back-diffusion of secreted hydrogen ions. This is secondary to an increase in passive permeability of the cell membrane, leading to bicarbonate wasting and the impairment of urine acidification. Therefore, AmB can develop classical distal (type 1) renal tubular acidosis [25]. Nephrogenic diabetes insipidus is mostly related to hypokalemia along with the inhibition of adenylyl cyclase and/or G proteins. These events decrease in the abundance of aquaporin 2 water channels and water permeability in the collecting duct [13,23].

Besides hemodynamic and tubular components, allergic interstitial nephritis is the other reported mechanism of AmB nephrotoxicity [14].

### 2.5. Risk Factors

Several variables have been identified and suggested as risk factors of AmB AKI (Table 1) [13,14,21,26,27,28,29]. Concerning the cumulative dose, AKI may be seen in nearly 30% and more than 80% of patients who received 240 mg and 5 g of AmB, respectively [14]. Although AmB serum levels above 1.0 mg/L have been considered as a potential risk for kidney impairment [18], a clear correlation between serum concentrations of AmB and its kidney safety has not been established so far [26]. A large retrospective study of 507 patients in Japan has identified prior treatment with ACE inhibitors/ARBs or carbapenem, concomitant administration of catecholamines or immunosuppressants (not defined), and daily dosing of AmB ≥ 3.52 mg/kg as factors significantly associated with AKI due to liposomal AmB [27]. Another retrospective study in tertiary care centers in Palestine has demonstrated that hypertension, COVID-19 infection, ICU admission, and mechanical ventilation are significantly associated with conventional AmB AKI [21]. Finally, concurrent treatment with piperacillin and ticarcillin has been reported as a predisposing factor of hypokalemia caused by AmB [28].

### 2.6. Clinical/Paraclinical Manifestations and Outcomes

**Figure 2:**
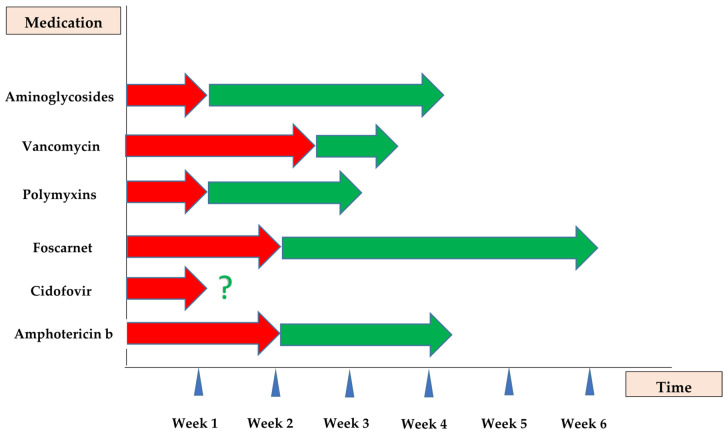
Time onset (depicted by red arrow) and time to recovery (depicted by green arrows) of AKI caused by amphotericin B, cidofovir, foscarnet, polymyxins, vancomycin, and aminoglycosides. These values are just approximations and ranges that have been reported from some relevant clinical studies. Delayed onset and prolonged recovery of AKI with these medications are possible. In the case of cidofovir, since the time to recovery from AKI has not been clearly described, it has been depicted by a question mark [14,32,33,34,35,36,37].

Most cases of AmB nephrotoxicity include a rise in serum creatinine as well as BUN concentrations and electrolyte disorders occurring during the first 2 weeks of treatment (Figure 2). Nevertheless, it may take several weeks to develop full-blown AmB AKI [14]. The serum creatinine level usually does not increase by more than 2.5 mg/dL from baseline values [22]. AmB AKI is usually dose dependent and shows clinical resolution upon discontinuing the treatment. Albeit, up to 15% of patients with AmB-associated AKI may require dialysis, at least temporarily [13]. A retrospective cohort study between January 2008 and March 2015 at Mayo Clinic in Rochester demonstrated that among 98 patients experienced AKI during liposomal AmB; 32 subjects exhibited complete resolution after a mean 9.8 ± 7.8 days. The median (interquartile range) time to partial resolution was 6 days (3–15 days) [30]. From the pharmacoeconomic point of view, AmB-associated AKI leads to a mean increased length of hospital stay by 8.2 days and adjusted additional costs by USD 29,823 per patient [31].

Although urinary potassium wasting, hypokalemia, urinary magnesium wasting, and hypomagnesemia are common with AmB, serious related complications such as rhabdomyolysis and life-threatening arrhythmias appear to be infrequent, especially in the absence of predisposing factors such as cirrhosis, congestive heart failure, and myocardial infarction [38]. Distal renal tubular acidosis due to AmB is characterized by a normal anion gap [25]. Nephrogenic diabetes insipidus caused by AmB is clinically characterized by reduced urine osmolality, polyuria, and polydipsia [22,23]. However, the risk of dehydration is low because affected patients are usually able to increase their urinary osmolality above plasma osmolality. Fortunately, nephrogenic diabetes insipidus due to AmB usually resolves clinically after discontinuation of the offending agent [39].

AmB-associated AKI includes various characteristics, such as an increased serum creatinine and/or BUN, impaired renal concentrating ability, renal tubular acidosis, and electrolyte abnormalities; so, it may either overlap with or contribute to other underlying disorders like dehydration, sepsis, or hereditary renal tubular acidosis. Therefore, the diagnosis of AmB-associated AKI should be carried out with a multifactorial approach considering the temporal/causal relationship, taking a complete history from the patient, conducting a physical examination, and performing different laboratory tests (e.g., kidney function test, urine analysis, serum/urine electrolytes, and urine output), even renal biopsy.

### 2.7. Biomarkers

Besides serum creatinine as a classic index of kidney function, other biomarkers of kidney function have been considered in a few experimental and clinical studies for the assessment of AmB-associated AKI. Some of these studied biomarkers are as follows: urinary osteopontin, N-acetyl-b-D glucosaminidase (NAG), α-glutathione S-transferase (a-GST), p-GST, clusterin, interleukin-18 (IL-18), beta-2-microglobulin, kidney-injury molecule-1 (KIM-1), neutrophil gelatinase-associated lipocalin (NGAL), and serum as well as urinary cystatin C [40,41,42,43]. AmB mostly causes damage to the distal tubule, where NGAL is one of its site-specific biomarkers. NGAL is a glycoprotein that expresses in various tissues, including the kidney. Its expression is markedly upregulated after kidney ischemia [3]. Accordingly, the ability of NGAL to predict AKI on the first day of AmB administration was significantly higher than serum creatinine in hematologic–oncologic patients with an area under the curve (AUC) = 0.765 and 95% confidence interval (CI) = 0.59 to 0.96 [44]. Another prospective cohort study of hemodynamically stable patients with leishmaniasis demonstrated that urinary NGAL detected AmB-associated AKI 3.2 days earlier than serum creatinine. The AUC for urinary NGAL on day 5 of AmB treatment was 0.89 (95% CI, 0.67 to 1.00) [45].

### 2.8. Prevention

During the recent five decades, numerous experimental and clinical studies have been performed to either prevent or minimize AmB AKI (Table 2). Among them, salt load and lipid-based formulations have more clinical evidence.

#### 2.8.1. Salt Loading

Salt loading during AmB therapy is one of the most evidenced-based interventions to prevent its AKI. The rationale behind this approach is that volume expansion via salt loading can block TGF induced by AmB. This may protect against or ameliorate the hemodynamic component of AmB AKI, mostly characterized by a decline in the GFR. Nevertheless, salt loading generally has no beneficial effects on tubular aspects of AmB nephrotoxicity including electrolyte disorders, distal renal tubular acidosis, and nephrogenic diabetes insipidus. For salt loading, intravenous sodium chloride at a dose of 150 mEq (1 L or 10–15 mL/kg of 0.9% sodium chloride solution) per day is given as either a bolus or continuous infusion along with the usual dietary sodium intake. Alternatively, ORS (1 L [90 mEq] 60 min before starting the infusion of AmB and 2 L throughout the rest of the day) may also be effective in preventing AmB-associated AKI. Suggested indicators of optimal salt loading are urinary sodium excretion of 250–300 mEq/day and urinary output of >2 L/day [13].

#### 2.8.2. Lipid-Based Formulations

Apart from salt loading, administering lipid-based formulations instead of conventional AmB is another well-documented approach to minimize its AKI. There are three commercially available lipid formulations of AmB, including AmB lipid complex (Abelcet^®^), AmB colloidal dispersion (Amphotec^®^ or Amphocil^®^), and liposomal AmB (AmBisome^®^). Presently, AmB colloidal dispersion is not commercially available in the United States. These products have different pharmacokinetics and rates of AmB release, resulting in less AKI and allowing for using higher doses [46]. Several mechanisms have been proposed to explain the reduced AKI of lipid formulations of AmB compared to conventional AmB. They include (1) a lack of deoxycholate, which has direct tubular toxicity (up to 40% of tubular toxicity of conventional AmB may be due to deoxycholate); (2) preferential distribution to the reticuloendothelial system that results in selective and slower transfer of AmB to targeted fungal rather than mammalian cell membrane (e.g., kidney); and (3) preferential binding to high-density lipoproteins. Regarding the last mechanism, there are low number of high-density lipoprotein receptors in kidney tubular cells that minimize the access of lipid formulations of AmB, especially liposomal AmB, to the kidney. This contrasts with conventional AmB that mostly binds to low-density lipoproteins [15,22]. Most head-to-head clinical trials along with meta-analyses have generally suggested that liposomal AmB causes less AKI and less severe hypokalemia than conventional AmB [10,15,22]. Interestingly, at least one case series has described the successful use of liposomal AmB for the treatment of invasive fungal infections in three critically ill patients with documented AKI [47]. Despite these benefits, it is noteworthy that the drug acquisition cost of a lipid-based formulations of AmB, particularly liposomal AmB, is significantly higher than that of conventional AmB. This is a dire issue in resource-limited settings [10,15].

#### 2.8.3. Preparing an Intravenous Lipid Emulsion

Preparing conventional AmB in intravenous lipid emulsions such as Intralipid^®^ 20% has been shown to reduce its nephrotoxicity in several clinical studies in different settings including febrile neutropenia, HIV, hematologic malignancies, and critical care [15]. Accordingly, a systematic review and network meta-analysis of 25 randomized controlled trials enrolling a total of 2996 patients identified AmB-intravenous lipid combination as the safest and most cost-saving treatment option in comparison with other formulations [48]. Besides positive findings, this approach has several advantages over using lipid-based formulations of AmB such as the convenience of preparing the admixture, availability of its components, and low cost of the combination. Nevertheless, preparing conventional AmB in intravenous lipid emulsions is currently discouraged in routine clinical practice and has not received FDA approval because of its potential pitfalls and concerns. These include the possible instability of AmB in lipid emulsions, disparity in the preparation method of AmB in fat emulsions, the lack of a standard storage, administration conditions, the optimal concentration of AmB, and, finally, the potential risk of pulmonary embolisms secondary to the formation of an AmB particle or precipitate during the preparation [15].

#### 2.8.4. Prolonging the Duration of Infusion

Traditionally, AmB is administered as a 2–6 h intravenous infusion [13]. Prolonging the duration of AmB infusion has been tested in several clinical studies to simulate a slow transfer of AmB, observed in lipid-based formulations, to achieve less nephrotoxicity. The findings of some studies with limited sample sizes in hematological malignancies, solid organ, and bone marrow transplant recipients are promising [13]. In addition, the only randomized controlled trial comparing continuous with non-continuous treatment of AmB in neutropenic patients showed significantly less nephrotoxic effects with the former technique of administration [49]. In line with these findings, a meta-analysis of five non-randomized clinical studies suggested that there is a trend for lower nephrotoxicity of AmB in continuous infusion (24 h) compared to conventional infusion (2–6 h) group [50]. Finally, a recently published retrospective, multicenter cohort study at two mixed medical–surgical ICUs demonstrated that continuous intravenous infusion of AmB in combination with therapeutic drug monitoring (TDM) of its serum levels (two times per week) was not associated with kidney impairment in critically ill patients with abdominal sepsis [51].

Despite positive findings, prolonging the duration of AmB infusion has several drawbacks and questions that should be considered and resolved before offering any recommendation in favor of or against its routine use. For example, continuous infusion of AmB is usually associated with an increase in nursing workload and a decrease in the feasibility of AmB administration to outpatients. More importantly, clinical efficacy of AmB may be adversely affected. In this regard, several animal studies have suggested that AmB has concentration-dependent fungicidal activity, at least against *Candida albicans* and *Cryptococcus neoformans* [13]; albeit, other studies have concluded that AmB has both time- and concentration-dependent antimicrobial behaviors, characterized by Cmax/MIC, AUC0–24/MIC and %T/MIC parameters [51]. Finally, possible physicochemical stability and compatibility issues of AmB with other medications during continuous infusion cannot be ignored [13].

#### 2.8.5. Co-Administering Diuretics

Diuretics has been investigated in few clinical studies to determine whether they can prevent or mitigate different aspects of AmB-associated AKI. As an osmotic diuretic, mannitol (12.5 g intravenously before and after each course of AmB infusion) has promising preliminary findings in four kidney transplant recipients in preventing AmB-induced azotemia [52]. In contrast, the only prospective, double-blind, controlled clinical trial failed to demonstrate that the co-administration of mannitol (1.0 g/kg added to AmB solution in 5% dextrose in water and infused over 4 h) is effective in protecting against a decrease in creatinine clearance and urinary concentrating ability [53]. Concomitant administration of potassium-sparing diuretics including amiloride (5.0 mg orally twice a day) [54] and spironolactone (100 mg orally twice a day) [55] has been reported to be both effective and safe in preventing hypokalemia and hypomagnesemia caused by AmB in patients with various hematological disorders.

#### 2.8.6. Co-Administering Nephroprotective Agents

Several agents with potential nephroprotective properties have been studied in a few clinical trials so far. These include low-dose dopamine (3 μg/kg/min) [56], the combination of intravenous isotonic sodium bicarbonate (500 mL) and sodium chloride 0.9% (500 mL) [57], oral pentoxifylline (400 mg twice a day) [58], and oral n-acetyl cysteine (600 mg twice a day) [59]. These agents were started at the day of AmB initiation and continued until either the completion of the treatment or the occurrence of AKI that needed AmB discontinuation. Except for n-acetylcysteine [59], co-administering the other above agents resulted in no significant changes in the studied indexes of kidney function. Although n-acetylcysteine demonstrated a significant reduction in the frequency of AmB-associated AKI (defined by an alteration in serum creatinine and estimated creatinine clearance), this agent failed in preventing or ameliorating AmB-induced electrolytes imbalances including hypokalemia, hypomagnesemia, and urinary magnesium and potassium wasting [59].

#### 2.8.7. Other Measures

In the hope of diminishing nephrotoxicity, some clinicians give AmB formulations with breakfast (a high cholesterol meal) to simulate lipid co-administration condition [60]. Based on results of a 9-year retrospective study [18], it has been suggested that patients with more than two risk factors for AmB nephrotoxicity can be assumed to be potential candidates for receiving alternative antifungal therapy with less nephrotoxicity such as itraconazole, voriconazole, and caspofungin [14]. Noting that the clinical relevance of these measures and considerations in preventing AmB AKI is currently unknown. If possible, concomitant administration of other nephrotoxins during AmB treatment should be avoided. Finally, if baseline creatinine clearance is less than 25 mL/min or initial serum creatinine is ≥2.5 mg/dL, it has been suggested not to initiate conventional AmB at all [61].

**Table 2:** Summary of studied strategies for preventing amphotericin b nephrotoxicity [13,15,56,57,58,59] *.

Strategy	Description
Salt loading	Administering intravenous sodium chloride at a dose of 150 mEq (1 L or 10–15 mL/kg of 0.9% sodium chloride solution) per day as a bolus or continuous infusion during the course of treatment is recommended.
Using lipid-based formulations	Using lipid-based formulations, including a lipid complex, colloidal dispersion, or liposomal (preferably), instead of conventional formulation is recommended.
Preparing in intravenous lipid emulsion	Preparing a conventional formulation in intravenous lipid emulsions such as Intralipid^®^ 20% may be effective.
Prolonging the duration of infusion	Administering a conventional formulation as a continuous 24 h infusion may be effective.
Co-administering diuretics	Potassium-sparing diuretics including amiloride (5.0 mg orally twice a day) or spironolactone (100 mg orally twice a day) may be effective in the management of electrolyte disorders including hypokalemia and hypomagnesemia.
Co-administering nephroprotective agents	Intravenous sodium bicarbonate + sodium chloride combination was ineffective in preserving GFR and reducing AKI. Low-dose dopamine (3 μg/kg/min) was ineffective in reducing the rate or severity of AKI. Oral pentoxifylline (400 mg twice a day) was ineffective in reducing AKI and electrolyte disorders. Oral n-acetyl cysteine (600 mg twice a day) was significantly effective in mitigating GFR drop and rate of AKI, but not tubular adverse effects (electrolyte disorders).
Using alternative antifungal therapy	Administering itraconazole, voriconazole, or caspofungin in patients with more than two risk factors of AKI or those with baseline creatinine clearance less than 25 mL/min.

* Some selected references. For complete references, please refer to the relevant subsection.

### 2.9. Monitoring

Regardless the type of preventive approach that has been implemented in clinical practice, close and regular monitoring of kidney function indexes and electrolytes is required during the treatment course of all intravenous formulations of AmB (Table 3) [14].

### 2.10. Treatment

In adult patients with no underlying kidney disease that develop an average serum creatinine level of 2 to 3 mg/dL at therapeutic doses of AmB, therapy generally should not be withheld [46]. Some experts recommend that if the serum creatinine concentration exceeds 2.5 mg/dL (in adults) or 1.5 mg/dL (in children) or reaches 2 mg/dL on 2 consecutive days during treatment, conventional AmB administration should be withheld and substituted with either lipid-based formulations or, preferably, other antifungal agents [14,61]. Within the course of kidney impairment, conventional AmB can be continued on alternate days by doubling the daily dose to a maximum 1.5 mg/kg/day [17]. During the period of AmB nephrotoxicity, serum magnesium, potassium, and calcium concentrations should be monitored daily and corrected as needed [14].

There is no definite and specific pharmacologic option for the management of AmB-associated AKI. Besides the role of salt loading in preventing or minimizing AmB AKI discussed above, this approach may also have beneficial impact on the management of AKI caused by this agent. In this regard, at least, a retrospective, multicenter observational study implicated that daily, consecutive liberal fluid infusion (such as isotonic saline) started from AKI onset was associated with a significant rate of resolution from stage 1 AKI in patients given liposomal AmB. This rate was 91% and 50% in liberal and conservative fluid management groups, respectively. Liberal fluid management was defined as administering daily fluid volume per weight being more than or equal to a specified threshold and for a determined period [80].

## 3. Cidofovir

### 3.1. Introduction

Cidofovir is the first monophosphate nucleotide analogue agent approved for use in humans [81]. It demonstrates in vitro activity against several DNA viruses, including the herpesviruses, adenovirus, polyomavirus, papillomavirus, and poxvirus [32]. In current clinical practice, cidofovir is mostly used and approved for the treatment of cytomegalovirus (CMV) retinitis in patients with acquired immunodeficiency syndrome (AIDS) who have failed to respond to ganciclovir [81]. Other possible clinical applications of systemic cidofovir are acyclovir-resistant varicella zoster virus (VZV) or herpes simplex virus (HSV) infections in either immunocompetent or immunocompromised patients, ganciclovir-resistant CMV, refractory BK virus-associated nephropathy in kidney transplant recipients, and BK virus- or adenovirus-associated hemorrhagic cystitis in hematopoietic stem cell transplant recipients [32].

### 3.2. Epidemiology

The most clinically important and dose-limiting side effect of cidofovir is nephrotoxicity [82]. During cidofovir treatment with a maintenance dose (5 mg/kg every other week), about 12% to 39% of patients may develop proteinuria, and 15% to 24% may experience elevated serum creatinine [32]. According to the package insert of the medication, proteinuria (2+ or greater), increased serum creatinine (rise of at least 0.4 mg/dL), or decreased creatinine clearance (55 mL/min or less) have been identified in about 50% of patients receiving cidofovir in clinical trials [81]. Fanconi syndrome has been observed in 1% of cidofovir recipients. Rare cases of chronic interstitial nephritis and nephrogenic diabetes insipidus caused by cidofovir have been also reported [33]. The rate of cidofovir-induced AKI in pediatric patients may be higher due to having longer medication half-life [83]. According to a retrospective study of pediatric patients with adenovirus infection, 9 out of 29 cidofovir treatment courses were associated with AKI [84].

### 3.3. Definition

Presently, there is no specific and conclusive definition for cidofovir-associated AKI in the literature. A retrospective study of cidofovir in pediatric patients defined AKI as an increase in the serum creatinine level of ≥0.3 mg/dL from baseline or requirement for continuous kidney replacement therapy or hemodialysis at any time during cidofovir therapy [84]. A more recent retrospective study of hematopoietic cell transplant recipients in adults has used the RIFLE criteria (specifically an increase in serum creatinine of ≥ 1.5 times baseline and/or a decrease in ClCr by >25%) for assessing kidney function in cidofovir recipients [85].

### 3.4. Pathophysiological Mechanisms

**Figure 3:**
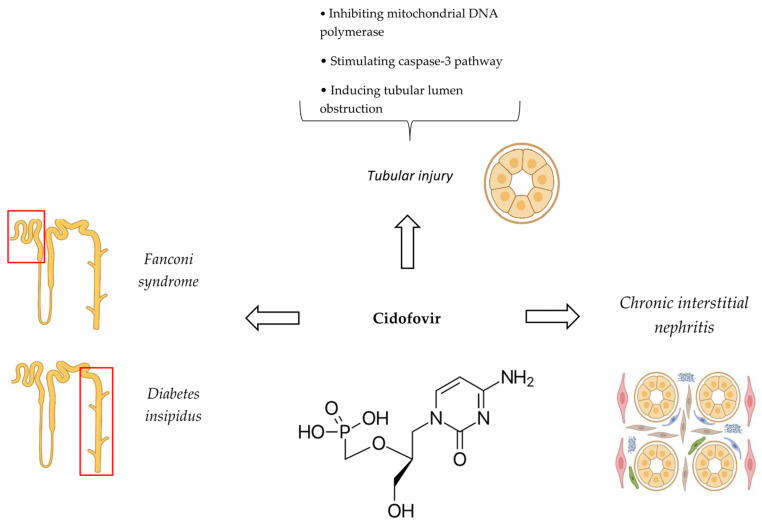
Summary of different aspects and pathophysiologic mechanisms of cidofovir nephrotoxicity [33,86,87].

The major mechanism of cidofovir nephrotoxicity is ATI [33]. This is secondary to the inhibition of mitochondrial DNA polymerase, leading to ATP depletion [86]. In addition, cidofovir can directly stimulate the caspase-3 pathway, a pro-apoptotic regulator [82]. The net consequence of these events is the apoptosis of proximal tubule epithelial cells [86]. Besides ATI, cidofovir can also cause tubular lumen obstruction that has an independent role in both the initiation and extension of tubular injury (Figure 3) [87]. The pathophysiologic pathways of cidofovir-induced chronic interstitial nephritis, diabetes insipidus, and Fanconi syndrome have not been described individually.

### 3.5. Risk Factors

The kidney safety of cidofovir has not been evaluated extensively in patients receiving other known potentially nephrotoxic agents (e.g., tenofovir, aminoglycosides, AmB, foscarnet, intravenous pentamidine, adefovir, and vancomycin) [34]. However, a recently published retrospective, large, multicenter cohort study of adult hematopoietic cell transplant recipients implicated liposomal amphotericin B and IV voriconazole/posaconazole as predictors of cidofovir nephrotoxicity [85]. As mentioned previously, since cidofovir has a longer half-life in children than adults, pediatric patients may be more prone to nephrotoxicity with this agent [83]. Cidofovir nephrotoxicity commonly occurs in a dose-dependent manner [34]. In neonates and older children, it has been clearly demonstrated that higher weekly doses of cidofovir were associated with greater increases in creatinine levels. Finally, previous history of kidney disease can be the other potential risk factor of cidofovir nephrotoxicity [84].

### 3.6. Clinical/Paraclinical Manifestations and Outcomes

Kidney dysfunction depicted by increased serum creatinine (at least 0.4 mg/dL) usually occurs one week after starting cidofovir [33,34] (Figure 2). According to a retrospective review of pediatric patients with adenovirus infection treated with cidofovir, the median time to kidney insufficiency was much shorter in the neonates than in the older children (2 vs. 20 days, respectively) [84].

Since ATI is the major mechanism of cidofovir nephrotoxicity, fractional excretion of sodium and urine osmolality are expected to more than 2% and less than 350 mOsmol/kg, respectively. Granular casts and kidney epithelial cells can be detected in urine analysis [86]. Cidofovir-associated AKI at least partially resolves in some patients. However, irreversible AKI leading to end-stage kidney disease has been also described in few case reports [81]. Fanconi-type syndrome due to cidofovir is generally characterized by proteinuria (2+ or greater), glucosuria, phosphoturia, bicarbonate wasting, and metabolic acidosis [80]. In the case of Fanconi-type syndrome caused by cidofovir, along with these laboratory abnormalities, clinical symptoms like bone pain and decreased muscle strength can be also helpful in the diagnosis [88]. Heavy metal exposure (e.g., cadmium and lead), aristolochic acid (as an herbal remedy) or paraquat poisoning, glue sniffing, Sjogren’s syndrome, and multiple myeloma are other possible causes of acquired Fanconi syndrome [89].

### 3.7. Biomarkers

Conventional biomarkers of kidney function including serum creatinine and serum urea are routinely used in the setting of cidofovir nephrotoxicity. Novel biomarkers of kidney function have not been specifically studied in relevant investigations so far.

### 3.8. Prevention

Hydration with one liter of normal saline is required 1 hour before infusing each dose of cidofovir; if patients can tolerate the fluid load, a second one liter will be given over a 1–3 h period either simultaneously during or immediately after completing the infusion [34].

Since cidofovir excretion in the proximal tubule of kidney is mostly mediated via the human organic anion transporter-1 (hOAT-1), probenecid as a hOAT-1 inhibitor can reduce drug uptake into the proximal tubule epithelium and prevent or minimize AKI [86]. Therefore, high-dose probenecid (2 g two hours before and 1 g two hours and eight hours after each infusion for a total of 4 g) can be given. To reduce the potential nausea/vomiting associated with the administration of probenecid, it has been suggested to administer each dose of probenecid with food or considering pretreatment with antiemetic agents [34]. Despite its potential efficacy, since the doses of cidofovir currently used are about 5% to 10% of the standard dose used for the treatment of CMV (0.25–1.0 mg/kg/dose), administering probenecid to prevent high-dose cidofovir-induced AKI may deem unnecessary in routine clinical practice [90].

Using alternative formulations of cidofovir with less nephrotoxicity, such as brincidofovir (CMX-001), is another potential preventive approach. This agent is not generally accumulated in the kidney tubular epithelial cells because it is not a substrate for hOAT1, located in the basolateral membrane. Several retrospective case studies have demonstrated the clinical effectiveness as well as kidney safety profile of brincidofovir in solid organ and hematopoietic stem cell transplantation [32,62]. In addition, findings of at least one randomized placebo-controlled phase II trial on pre-emptive treatment with brincidofovir for the prevention of adenovirus disease in pediatric and adult allogeneic HCT recipients also supports that this agent lacks nephrotoxicity [91]. However, much more robust clinical evidence is needed to determine the real role of brincidofovir as an alternative agent of cidofovir with less nephrotoxicity in clinical practice.

The co-administration of cidofovir with other nephrotoxic agents, especially tenofovir disoproxil fumarate, is contraindicated because of increasing the risk of Fanconi syndrome [34]. In addition, some of the literature has suggested that all potentially nephrotoxic agents, such as nonsteroidal anti-inflammatory drugs, contrast media, aminoglycosides, AmB, foscarnet, and pentamidine should also be discontinued at least seven days before starting cidofovir [86]. Finally, the initiation of cidofovir is relatively contraindicated in patients with baseline serum creatinine more than 1.5 mg/dL, calculated creatinine clearance less than 55 mL/min, or with underlying significant proteinuria (≥100 mg/dL in 24 h urine or ≥2+ in spot urine) [34,92]. These preventive measures are listed and summarized in Table 4.

### 3.9. Monitoring

Kidney function should be determined and monitored before and during each course of cidofovir treatment. Proteinuria appears to be an early and sensitive indicator of cidofovir nephrotoxicity (Table 3) [62].

### 3.10. Treatment

It has been advised that cidofovir treatment should be withheld temporary and intravenous hydration should be initiated if serum creatinine increases by ≥0.5 mg/dL or persistent ≥2+ proteinuria develops. If these increments are still observed despite hydration, cidofovir therapy should be discontinued permanently. The benefit–risk balance of reintroducing cidofovir is currently unknown [34]. No pharmacologic agent or procedure to attenuate cidofovir-associated AKI has been studied so far. Severe AKI caused by cidofovir may require dialysis [32]. The serum levels of cidofovir can be reduced by about 75% with high-flux hemodialysis [34].

## 4. Foscarnet

### 4.1. Introduction

Foscarnet is a pyrophosphate analog with polymerase and reverse-transcriptase inhibitor activities [32]. It has in vitro antiviral effects against herpes family viruses, hepatitis B virus, and even the human immunodeficiency virus (HIV). In clinical practice, it is almost exclusively employed for the prevention/treatment of ganciclovir-resistant CMV or patients intolerant to ganciclovir, acyclovir-resistant HSV, and acyclovir-resistant VZV [35]. Although it acts synergistically with zidovudine in inhibiting HIV replication [32], foscarnet currently has no clinical indication for the treatment of HIV [35].

### 4.2. Epidemiology

Foscarnet has a narrow therapeutic index, and its administration is associated with a myriad of adverse effects. Nephrotoxicity is usually considered as a major dose-limiting adverse effect [32]. The first cases of foscarnet-induced AKI were described in 5 out of 37 kidney biopsies or necropsies in HIV patients in the 1990s [93]. According to the findings of clinical trials in AIDS, about one-third of patients may develop significant kidney impairment, defined as serum creatinine ≥2 mg/dL, during foscarnet treatment [32,35]. Similarly, in the setting of hematopoietic stem cell transplantation, deterioration in kidney function indexes (e.g., serum creatinine clearance) was reported in 27% to 50% of foscarnet recipients [94]. A retrospective survey on records of both solid organ and hematopoietic cell transplant over a 10-year period demonstrated that kidney dysfunction occurred in 51% and 28% by the end of foscarnet treatment and after 6 months, respectively [95]. The rate of foscarnet-associated AKI in lung transplantation has even reached up to 71% [96]. The frequency of other possible kidney involvements of foscarnet, including Fanconi syndrome, urinary magnesium wasting, nephrogenic diabetes insipidus, renal tubular acidosis, and interstitial nephritis, has not been stated clearly in the literature.

### 4.3. Definition

There is lack of operational criteria for determining foscarnet-associated AKI, and various criteria have been used in clinical studies. For example, a >20% decrease in estimated GFR by the MDRD method is considered kidney dysfunction in foscarnet recipients [95]. Another study used criteria of kidney dysfunction as serum creatinine increase ≥100% or creatinine clearance decrease ≥50% from baseline values [97]. Finally, two recently published studies in hematopoietic cell transplantation settings have provided a criterion of doubling baseline serum creatinine [94] or KDIGO criteria [98] for foscarnet-associated AKI.

### 4.4. Pathophysiological Mechanisms

It appears that foscarnet has direct toxic effects on the renal tubular cells, causing ATI [14]. Additionally, this agent can form complexes with divalent cations including Ca^2+^, Mg^2+^, Fe^2+^, and Zn^2+^ [99]. In the case of ionized calcium, it may result in the precipitation of calcium–foscarnet salt crystals. This precipitate in the tubular cells or tubular lumens, also called crystal nephropathy, partially contributes to ATI [14]. Apart from tubules, calcium–foscarnet salt crystals can also precipitate in kidney glomeruli and the interstitium, causing crystalline glomerulonephritis and interstitial nephritis, respectively. The formation of other types of crystals such as phosphonoformate has been also reported to be associated with the development of foscarnet nephrotoxicity. Crystallization of foscarnet in urine does not seem to be pH dependent [100].

**Figure 4:**
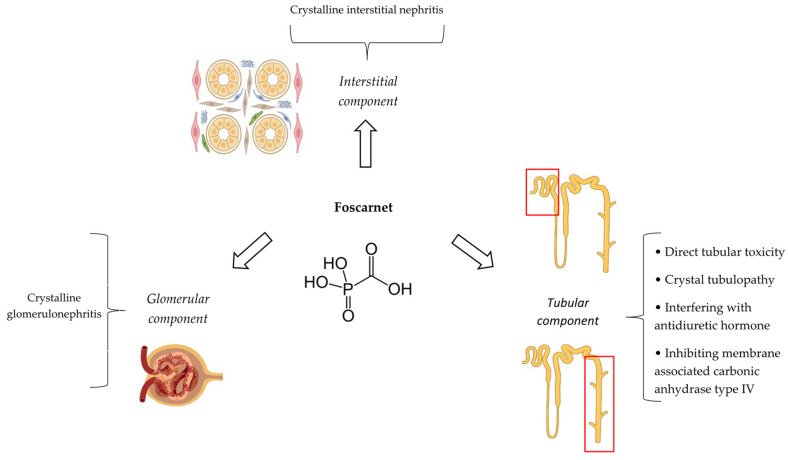
Summary of different aspects and pathophysiologic mechanisms of foscarnet nephrotoxicity [14,33,86].

Foscarnet-induced nephrogenic diabetes insipidus mostly mediates by interfering with the action of antidiuretic hormone on water channel aquaporin-2 located in the collecting duct of the kidney [33]. This can be independent of elevation in the serum creatinine concentration [32]. The precise mechanisms of distal tubular acidosis and magnesium kidney losses caused by foscarnet have remained mostly undetermined. The inhibition of membrane-associated carbonic anhydrase type IV by foscarnet may play a role in the pathogenesis of these kidney involvements [86]. Figure 4 depicts different aspects of foscarnet nephrotoxicity.

### 4.5. Risk Factors

High dosages (6–12 g per day), rapid infusion (less than 1 h and 2 h for induction and maintenance phases, respectively), dehydration, and concomitant administration of nephrotoxic medications appear to increase the risk of foscarnet nephrotoxicity [32]. These nephrotoxic medications include aminoglycosides, AmB, pentamidine, trimethoprim–sulfamethoxazole, cyclosporine, tacrolimus, furosemide, cidofovir, acyclovir, ritonavir, and saquinavir [14,63,92,101,102].

### 4.6. Clinical/Paraclinical Manifestations and Outcomes

The serum creatinine value usually begins to rise 6 to 15 days after starting foscarnet [35]. In most cases, it resolves within 2 to 4 weeks after cessation of foscarnet (Figure 2) [32]. However, the adverse impacts of foscarnet on kidney function can be irreversible [33]. For example, in allogeneic hematopoietic cell transplant, foscarnet recipients have been reported to experience a continued decline in kidney function between months 6 and 12 after its administration [103].

During the period of foscarnet-associated AKI, urinalysis may demonstrate glomerular- or non-glomerular hematuria, leukocyturia, proteinuria, and tubular epithelial cells [32,99,100]. Foscarnet crystals in urinary sediment exhibit rectangular and square shapes. However, the presence of crystalline casts in urine sediment (crystalluria) are not necessarily indicative of or diagnostic for AKI. Even with AKI development, it may be a lag time of about two weeks between the appearance of crystalluria and AKI development [100]. In the presence of hematuria and even modest proteinuria, glomerulonephritis should be considered as possible differential diagnosis. A clear temporal relationship, excluding alternative causes of AKI, and detecting crystal-containing casts in the urine sediment are major diagnostic criteria for foscarnet crystal-induced nephrotoxicity. Notably, definitive diagnosis can be achieved by the kidney biopsy [104].

Foscarnet-induced nephrogenic diabetes insipidus clinically manifests by polyuria and polydipsia with or without elevation in the serum creatinine concentration [35]. Diabetes mellitus and primary polydipsia can also cause polydipsia and polyuria, and they should be ruled out [105].

### 4.7. Biomarkers

In the context of foscarnet-associated AKI, apart from serum creatinine as a traditional index, novel biomarkers of kidney function appear to be addressed in only one prospective, case-control clinical study so far. In this regard, hospitalized patients treated with potentially nephrotoxic medications for at least one week such as foscarnet were recruited. Absolute, normalized to urine creatinine TIMP2 as well as IGFBP7, and their composite urinary levels (TIMP2-IGFBP7) were significantly elevated in AKI cases compared to non-AKI subjects, as early as 2 to 3 days prior to AKI onset. Interestingly, the normalized urinary TIMP2 level had the best average AUROC of 0.81 to predict AKI and outperformed urinary IGFBP7 alone or TIMP2-IGFBP7. In contrast, serum creatinine levels between AKI and non-AKI groups did not show statistical significance at 2 to 3 days prior to the event, with a corresponding average AUROC for subsequent AKI prediction of 0.50 [106].

### 4.8. Prevention

Adequate hydration is a documented strategy to prevent or minimize foscarnet-associated AKI [63]. Intravenous hydration has dramatically reduced the incidence of foscarnet-associated AKI from 60% to 10–20% [107]. The major rational for hydration is decreasing the urinary concentration of the drug, mitigating the chance of crystal formation in urine [100]. To establish adequate diuresis, 750 to 1000 mL of normal saline (preferably) or 5% dextrose should be administered intravenously immediately before the first course of infusion. With subsequent infusions, 500 to 1000 mL of intravenous fluids should be given, which depends on the foscarnet dose [63]. Some references have suggested higher volumes for intravenous hydration (1.5 to 2.5 L per day) [100]. In patients with fluid overload, less, rather than not none, hydration may be given [65].

Oral hydration (at least 16 ounces [450 mL] of fluid until achieving the urine output of 200 mL) has been also implicated to be a convenient alternative approach to intravenous hydration for preventing foscarnet AKI. The type of oral fluid was liberal as well as optional. However, patients were advised against drinking alcohol or caffeine-containing beverages. Nevertheless, the rate of foscarnet-induced AKI has been reported to be higher with oral hydration compared to intravenous hydration (20% and 11%, respectively) [108].

There is a greater ease of administering foscarnet as a continuous infusion (24 h) compared to conventional intermittent infusion (at least 1 to 2 h), especially in outpatients [98]. There is a possibility that continuous infusion of foscarnet allows for the safe administration of outpatient treatment in patients at high risk for nephrotoxicity with CMV reactivation and/or disease [109]. However, a retrospective, single-center observational study reported that the rate of adverse reactions, most notably AKI, was comparable between intermittent infusion and continuous infusion groups. In other words, continuous infusion of foscarnet has no superiority and advantage over intermittent infusion in the terms of nephrotoxicity [98].

In patients with pre-existing moderate-to-severe kidney insufficiency, defined by baseline serum creatinine levels greater than 2.8 mg/dL or measured 24 h creatinine clearances < 50 mL/min, it has been advised not to start foscarnet [63,66]. Table 5 has summarized the suggested strategies for preventing foscarnet nephrotoxicity.

### 4.9. Monitoring

Serum creatinine, creatinine clearance, and serum electrolytes should be measured and monitored during the induction and maintenance phases of foscarnet treatment (Table 3).

### 4.10. Treatment

Treatment is completely supportive. If the calculated creatinine clearance drops below 0.4 mL/min/kg during the treatment, foscarnet should be discontinued [66]. Currently, there is no pharmacologic agent for the treatment of foscarnet-induced AKI. Since foscarnet crystal formation is independent of urine pH, alkalization of urine probably has no role for crystal solubilization [100]. In the case of severe AKI, performing temporary dialysis may be inevitable. Serum concentrations of foscarnet are reduced to 50% by hemodialysis [86].

## 5. Polymyxins

### 5.1. Introduction

Colistin, also known as polymyxin E, and polymyxin B belong to the polymyxin drug class. This class of polypeptide, amphipathic antibiotics exert their bactericidal effects against Gram-negative bacteria through binding to lipopolysaccharides and phospholipids [110]. Currently, polymyxins are mostly used for the treatment of infections caused by multidrug-resistant organisms in critically ill patients. These include carbapenem-resistant Enterobacteriaceae (*Escherichia coli*, *Klebsiella pneumoniae*, and some *Enterobacter* spp.), *Pseudomonas aeruginosa*, and *Acinetobacter baumannii* [110,111].

### 5.2. Epidemiology

Polymyxins have a narrow therapeutic window [112]. The most common side effect of polymyxins that limits their clinical use is nephrotoxicity [113]. The frequency of colistin-associated AKI varies greatly in the literature ranging, from 0% to 76% [69]. The 2019 polymyxin use guidelines have estimated AKI events to between 20% and 50% after exposure for both polymyxins [67]. A meta-analysis of 95 observational studies including 7911 patients found that the colistin- and polymyxin B-associated AKI cases were 26.7% (95% CI, 22.8–30.9%) and 29.8% (95% CI, 23.8–36.7%), respectively [114]. A more recent meta-analysis of five randomized clinical trials with a total of 377 patients, mainly critically ill patients, reported that the incidence of colistin-associated AKI was 36.2% (95% CI, 23.3–51.3%) [115]. A systematic review and meta-analysis of eight studies involving 6199 adult patients reported the pooled incidence of colistin- and polymyxin B-induced AKI using the RIFLE criteria to be 48% (95% CI: 42–54%) and 38% (95% CI: 32–44%), respectively [116]. In neonates and infants, colistin-associated AKI was reported in 5.8% of patients [117]. Finally, in a systematic review and meta-analysis of seven studies including 405 critically ill pediatric patients who received intravenous colistin, AKI was detected in 6.1% of its recipients [118].

This wide variation in the occurrence of polymyxin-associated AKI is mostly due to disparity as well as heterogeneity in the operational criteria for determining AKI, the study population, study methods, medication dose, severity of illness, and presence of other potential confounders such as co-administered nephrotoxic agents [67,115]. Interestingly, there is a growing body of data from observational studies suggesting that the AKI occurrence with colistin is higher than that with polymyxin B [119]. Recently published data from a retrospective cohort study in a university hospital in Turkey suggested that the incidence of AKI during treatment was higher in the colistin compared to polymyxin B group (52.6% and 34.7%, respectively, *p* = 0.013) [120]. Pairwise meta-analysis showed higher rates of AKI with colistin compared with polymyxin B (42.7% and 21.3%, respectively), although not reaching the level of statistical significance (OR = 2.37; 95% CI, 0.62–9.07; *p* = 0.206) [121]. Similar findings were reported from another meta-analysis [122]. In contrast, besides several clinical studies [69], at least a meta-analysis suggested that there was no significant difference in AKI prevalence between polymyxin B and colistin [114]. Therefore, the real clinical relevance of this possible difference between colistin and polymyxin B AKI remains to be elucidated.

### 5.3. Definition

Numerous operational criteria of polymyxin-associated AKI have been used in clinical studies. According to traditional criteria, a rising serum creatinine level of more than 2 mg/dL (only in patients without pre-existing kidney dysfunction), a 50% reduction in calculated creatinine clearance in comparison with the baseline value, or a decline in kidney function that requires kidney replacement therapy is determined as polymyxin AKI. The calculated creatinine clearance in these criteria is based on the Cockroft–Gault formula [123].

By the introduction of more novel and standardized criteria for AKI in clinical practice during the recent two decades including RIFLE and KDIGO, most new studies have utilized these two criteria to describe polymyxin-associated AKI and categorize its severity. For example, a retrospective cohort study that compared AKI events between two different formulations of colistin in 195 critically ill patients between January 2016 and October 2018 in Taiwan defined AKI based on the KDIGO guidelines [124]. Another retrospective study assessing population pharmacokinetics of colistin sulfate in 42 critically ill patients from January 2020 to December 2021 at a university hospital in China assessed AKI by using the RIFLE criteria [125]. This definition has been also utilized in a prospective, observational study at the medical and surgical ICU of a university hospital in Turkey [126].

### 5.4. Pathophysiological Mechanisms

Although polymyxins were introduced in the 1950s and temporary withdrawn from the market mostly because of their nephrotoxicity since the 1970s, knowledge about their mechanisms of nephrotoxicity has significantly increased only within the last decade [127].

The major mechanism of polymyxin nephrotoxicity is tubular damage, mostly in the proximal tubules and, to a lesser extent, in distal tubules, leading to ATI. This occurs because of substantial intracellular accumulation of the drug mediated by the membrane endocytic receptors including megalin and peptide transporter 2 [113]. The accumulation of polymyxins increases membrane permeability, since they are cations with high lipid affinity that can bind to the phospholipids of the kidney tissue. Besides membrane permeability, stress oxidative and apoptosis have also a crucial role in the pathogenesis of polymyxin nephrotoxicity [128]. An increase in oxidative damage is characterized by the rise in reactive oxygen species (ROS) and the reduction in superoxide dismutase 2 (SOD2), endothelial nitric oxide synthase (eNOS), and glutathione (GSH) levels. Peroxidation of membrane lipids, proteins, and, especially, DNA can stimulate apoptosis by triggering apoptotic factors such as p53, cytochrome c, and Bcl-2 [113]. In addition, mitochondrial and endoplasmic reticulum damage due to oxidative stress can also activate proapoptotic genes and related proteins including caspase-9 and caspase-12. Interestingly, polymyxins can directly bind to Fas as a death receptor, leading to caspase-8 and caspase-3 activation, culminating in DNA fragmentation and cellular apoptosis [127,128]. Finally, the matrix metalloproteinase 3 level, an enzyme that contributes to tissue repair, could be decreased by polymyxins [112].

**Figure 5:**
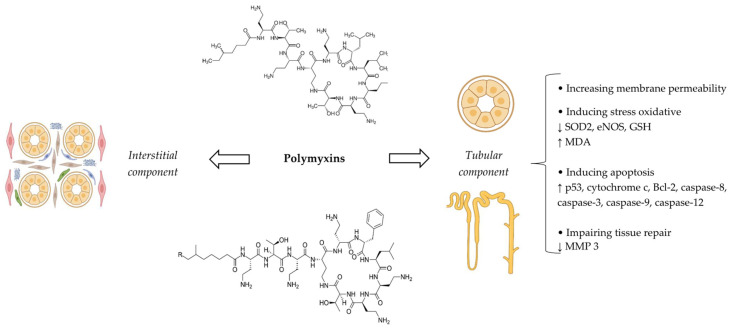
Summary of different aspects and pathophysiologic mechanisms of polymyxin nephrotoxicity [113,127,128].

Apart from ATI, few case reports of acute interstitial nephritis secondary to hypersensitivity reactions of polymyxins have been also described in the literature [129,130]. One case of acute interstitial nephritis was documented by kidney biopsy [128]. A summary of the different aspects and pathophysiologic mechanisms of polymyxin nephrotoxicity is depicted in Figure 5.

The relative difference in potential nephrotoxicity of colistin (polymyxin E) and polymyxin B has been mostly attributed to the different pharmacokinetics properties of these two agents, especially at the distribution and excretion stages. In this regard, the extent of uptake and accumulation in the tubular cells of polymyxin B are lower than that of colistin. This is at least partially secondary to the shape of the plasma concentration-versus-time profiles. The larger “peak-to-trough” fluctuation observed with polymyxin B compared to colistin, which has relatively “flat” profile, can result in different degrees of tubular reabsorption saturability. However, there is insufficient evidence to support this hypothesis. Inefficiency of colistimethate sodium, as a prodrug of colistin, and inadequate processing of colistin as well as colistimethate sodium by the kidneys compared to polymyxin B is another possible explanation for this difference [131]. Besides the type of polymyxin, various formulations of a certain polymyxin may also have different potential for developing AKI. In this regard, a retrospective cohort study of critically ill patients implicated that the rate of stage 2 and stage 3 AKI with Colimycin^®^ was significantly lower than that with Locolin^®^. Not having the same compositions of colistimethate sodium A and colistimethate sodium B, which differ in the fatty acid chain attached to the cyclic decapeptide moiety of the drug, can partially account for the variable risk of nephrotoxicity with different formulations of colistimethate sodium [124].

### 5.5. Risk Factors

Despite polymyxin AKI being deemed to be a common adverse effect, its incidence, severity, and both short- and long-term clinical impacts are largely dependent on the presence of different risk factors (Table 6). These risk factors can be classified into patient related, medication related, and co-administered medications [36,69,116,123,127].

Concomitant use of one or more nephrotoxic medication has been implicated to increase the risk of polymyxin AKI in most relevant studies. More realistically in clinical practice, some studies have suggested the co-administration of at least two or three potentially nephrotoxic agents as a cut-off for significant association with polymyxin nephrotoxicity [132,133,134]. Surprisingly, at least one clinical trial has demonstrated that the incidence of AKI, determined by RIFLE, was significantly higher in the monotherapy group (colistin alone) compared with the combination group (colistin + meropenem) [135]. In line with this finding, a multicenter, retrospective cohort study of 4910 patients has implicated that the frequency of AKI was significantly lower in patients receiving colistin + minocycline than colistin alone [136]. Similarly, glucocorticoid co-treatment was independently associated with a reduced risk of AKI following colistin treatment in critically ill patients (OR = 0.23, 95% CI = 0.10–0.53; *p* = 0.001) [137]. These results are supported by a systematic review and meta-analysis of 4 clinical trials and 14 observational studies in adults with *A. baumannii* infection. Despite using the same doses, the incidence of AKI was higher in colistin monotherapy (OR = 1.66, 95% CI= 0.99–2.78, *p* = 0.05) [138]. One possible justification for these observations is that combination therapy is associated with increased microbiological/inflammatory responses to treatment and earlier recovery from sepsis, leading to a decreased risk of multi-organ failure, including the kidneys [127]. In the case of glucocorticoid exposure, inducing P-glycoprotein by these agents may cause the efflux of colistin from the renal proximal tubular cell epithelium [137]. Notably, the findings of at least three other meta-analyses do not support the nephroprotective effect of combination therapy compared to monotherapy in polymyxins recipients [122,139,140].

Daily dose, cumulative dose, and also dose calculation based on actual body weight (ABW) instead of ideal body weight (IBW) in patients with obesity have been suggested to be other possible risk factors of polymyxin nephrotoxicity [36]. For example, a retrospective study of 126 ICU patients demonstrated a daily dose of ≥5.0 mg/kg/day colistin based on IBW as an independent risk factor for AKI [132]. In the case of polymyxin B, daily doses equal or more than 200 mg polymyxin B were significantly associated with an increased risk of severe kidney insufficiency (OR = 4.5, 95% CI: 1.6–12.9) in 276 patients, of whom 218 (79.0%) were in the ICUs of 14 Chinese teaching hospitals [141]. In terms of cumulative dose, the median total dose of 41.6 mg/kg colistimethate sodium was significantly associated with AKI in a retrospective cohort study [142]. In contrast, several RIFLE-based studies in predominantly ICU-based patient populations have failed to discover any significant association between either daily or cumulative intake of polymyxins and AKI [36]. Finally, two recently published systematic reviews and meta-analyses reported that a higher daily dose was associated with an increased risk of polymyxin AKI (OR = 1.89, 95% CI = 1.40–2.55, *p* < 0.001; OR= 1.46, 95% CI 1.09–1.96, *p* = 0.012) [116,122]. Like the daily and cumulative total dose, the conclusive link between the loading dose of polymyxins and its nephrotoxicity is presently unclear [127]. In patients with obesity, using ABW as opposed to IBW for calculating the daily dose of colistin was linked to 13.2 times greater possibility of developing AKI [143].

Regarding laboratory abnormalities, hypoalbuminemia and hyperbilirubinemia may have an association with polymyxin AKI [127,144]. In terms of hyperbilirubinemia, patients with total bilirubin >5 mg/dL have experienced significantly higher rates of AKI with polymyxins [142]. Although the exact mechanism of hyperbilirubinemia in the development or exacerbation of polymyxin AKI is undetermined so far, similar pathways that have been suggested and described in the context of aminoglycoside nephrotoxicity may also be involved here [36]. Concerning hypoalbuminemia, different criteria have been used in clinical studies (e.g., serum albumin level less than 2.5 g/dL or 3.2 g/dL) [145,146]. Interestingly, a study of critically ill patients has determined the optimal cut-off value of 2.65 g/dL for serum albumin as a predictor of polymyxin AKI [147]. Finally, a systematic review and meta-analysis about polymyxin-induced AKI and its predictors implicated that patients with a high baseline serum albumin level were 0.69 times less likely to experience AKI compared to those with low serum albumin (*p* = 0.001) [116]. Concerning the mechanism of hypoalbuminemia, it is hypothesized that patients with low albumin levels may have higher serum levels of free colistin because the medication is 50% protein bound and preferentially binds to albumin [145]. This might result in the accumulation of more drug in kidney tubular cells, enhancing the risk of AKI. Moreover, hypoalbuminemia per se may also reflect the severity of the underlying illness [148].

There are scare clinical data about the possible association between polymyxin pharmacokinetics parameters such as the serum level and its AKI. This is mostly due to the lack of a precise and accurate analytical technique of measurement [127]. In a prospective, observational cohort study in Spain, a trough level of colistin more than 3.3 µg/mL on day 7 of therapy was identified to be an independent risk factor of AKI both on day 7 and also at end of treatment with colistimethate sodium [133]. A pharmacokinetic/toxicodynamic analysis of 153 critically ill patients implicated that the rates of AKI were substantially higher in patients with an average steady-state plasma colistin concentration greater than 2 μg/mL [149]. In line with these results, a prospective, observational study of 57 adult critically ill patients in Colombia demonstrated that the incidence of AKI in patients with low (<2 μg/mL), normal (2–4 μg/mL), and high (>4 μg/mL) colistin trough levels was 20.7%, 50%, and 50%, respectively [150]. Besides the trough level, a prospective cohort study of 25 patients (of which 64% were admitted to ICU) in a teaching hospital in South Korea implicated that the maximum plasma colistin concentration at day 7 and the end of treatment was significantly higher in patients who developed AKI [151]. Finally, Yang et al., by using a validated Bayesian approach in a retrospective observational study of 393 ICU patients, determined that area under the concentration–time curve across 24 h at steady state (AUCss, 24 h) of polymyxin B > 99.4 mg h/L and 100 mg h/L is a significant predictor of AKI based on classification/regression trees and cox/logical regression analyses, respectively [152].

### 5.6. Clinical/Paraclinical Manifestations and Outcomes

Polymyxin-induced AKI is characterized by a rise in serum creatinine that usually occurs within the first 5–7 days of treatment [36]. The median time of onset of AKI caused by polymyxins has been reported to be 7.5 days (Figure 2) [142]. Interestingly, in a retrospective study of 119 patients in four hospitals in Korea, of which more than three-fourths (78.2%) of them were hospitalized in the ICU, AKI occurred in 46 and 19 patients within 7 days and after 7 days of colistin treatment, respectively [152]. According to more recent observational, cross-sectional study in ICU and non-ICU wards of two hospitals in Iran, the mean ± SD onset of colistin-associated AKI was 5.73 ± 2.53 days (minimum–maximum of 4 and 13 days) [153].

In the setting of polymyxin-induced ATI, an increase in serum creatinine and a decrease in the clearance of creatinine can be accompanied with hematuria, proteinuria, cylindruria (presence of casts in the urine), and even oliguria [123,154]. In an observational study of 17 patients with a history of ICU admission who received intravenous colistin for more than 4 weeks, the median serum creatinine value increased by 0.25 mg/dL during the treatment compared to the baseline. The median maximum value of blood urea in these patients was 60 mg/dL [155]. A cross-sectional, observational study of 33 adult ICU patients demonstrated that the mean (95% CI) difference in serum creatinine values between patients with and without colistin-associated AKI was 1.026 mg/dL (0.704 to 1.349) [156]. In one of the interstitial nephritis cases caused by polymyxins, fever, oliguria, rapid weight gain, and eosinophilia were reported [129]. The other case report described fever along with rapid worsening of kidney function indexes including serum creatinine and BUN. However, rash, eosinophiluria, proteinuria, glucosuria, hematuria, pyuria, and hemodynamic disturbance were absent [130]. To differentiate ATI and interstitial nephritis, as aspects of polymyxin nephrotoxicity, from other possible causes like prerenal disease or glomerulonephritis, history taking as well as physical examination along with kidney function tests are all helpful but not enough. Therefore, urinalysis/urine microscopy, calculating the fractional excretion of sodium, evaluating the response to fluid repletion, and even performing renal biopsy may be necessary to confirm the diagnosis of polymyxin AKI in clinical practice [157,158].

Most cases of polymyxin-associated AKI are mild to moderate and expected to have clinical resolution [123]. This occurs by either discontinuing treatment or decreasing the established dose [144]. However, up to 28% of patients affected by polymyxin AKI may require kidney replacement therapy, at least temporarily [69]. Regarding clinical outcomes, according to a prospective, observational study in an adult ICU tertiary care hospital, among patients who developed kidney failure with colistin and polymyxin B, 75% and 83.3% recovered from AKI within 1 week, respectively [159]. Another study of both critical and non-critical patients reported a median (range) complete clinical resolution period of 10 days (7–15 days) after discontinuing colistin [119]. A retrospective study of 91 critically ill patients in Turkey has demonstrated that ICU mortality was significantly higher in patients with colistin-associated AKI (64.1%) compared to those without AKI (37%) [160]. Similarly, the ICU mortality rate differs significantly in patients with and without colistin-associated AKI (29 out of 39 [74.36%] and 8 out of 17 [47.06%] cases, respectively) [147].

### 5.7. Biomarkers

Apart from experimental studies, novel biomarkers of kidney function in the setting of polymyxin AKI have been also the subjects of several clinical investigations. These biomarkers include but are not limited to urinary KIM-1, urinary liver-type fatty-acid-binding protein (L-FABP), and urinary/plasma NGAL [3,161]. KIM-1 is a cell membrane glycoprotein, which is upregulated in the presence of nephrotoxic/ischemic damage to proximal tubule epithelial cells. L-FABP is expressed in the proximal tubule, and its expression is augmented by hypoxic stress [161]. Regarding KIM-1, a preliminary study of three patients demonstrated that in the setting of colistin-associated AKI, a more than 10-fold elevation in the normalized KIM-1/urine creatinine ratio from the baseline value was observed 6 days prior to the clinically significant increase in serum creatinine [162]. In 23 adult colistimethate sodium recipients, of which 11 were hospitalized in the ICU, a plasma NGAL level more than 285 ng/mL at 56 h after treatment was a powerful predictor of AKI, with an area under the receiver operating characteristics curve (AUC) of 0.796 (95% CI, 0.609 to 0.983) [163]. A prospective, observational study of 42 multidrug-resistant septic patients treated with colistimethate sodium demonstrated that subclinical AKI defined as urinary L-FABP more than 10.5 ng/mL was detected in 45.2% and 54.8% on days 5 and 7 of treatment, respectively. Interestingly, when compared with urinary NGAL, baseline L-FABP was significantly superior for the prediction of clinical AKI on day 5 of treatment [164].

### 5.8. Prevention

Preventive strategies of polymyxin AKI mostly rely on the following approaches and concepts (Table 7):

#### 5.8.1. Selecting the Type of Polymyxin

As discussed above, there is currently no clear advantage regarding kidney safety of polymyxin B over colistin. Nevertheless, some physicians may consider polymyxin B as a polymyxin of choice for the treatment of invasive infections. However, there is inadequate clinical data about the administration of polymyxin B via the intraventricular or intrathecal routes. In addition, colistimethate (the inactive prodrug of colistin) is usually preferred for urinary tract infections [127].

#### 5.8.2. Modifying Loading/Daily Dose, Administration Interval, and Infusion Duration

Inappropriate daily dosing of polymyxins in clinical practice is usually the result of confusion and possible controversies about doses prescribed according to colistimethate sodium versus colistin base, ignoring baseline kidney function indexes for possibly required dose adjustments and considering ABW instead of IBW for dose calculation of colistin in individuals with obesity [36,110]. Colistimethate sodium to colistin base dose conversions and dose adjustment based on kidney function (e.g., calculated creatinine clearance or serum creatinine) charts have been provided and are freely available in the literature [67,110]. Regarding loading dose, since it has not been conclusively shown to be a risk factor for AKI, its administration is recommended, especially in the case of severe infections caused by bacteria with a high MIC [127].

To optimize and individualize dosage regimens of polymyxins with the aim of minimizing the likelihood of AKI, data of population pharmacokinetics model (5000 simulated patients) have been exploited to develop an adaptive feedback control algorithm. The superiority of this model compared to a “one dose fits all” approach in achieving the target range of polymyxin B (2–4 mg/L) has been demonstrated in an in silico setting. Accordingly, the percent of simulated patients with supratherapeutic plasma levels of polymyxin B was significantly lower with the feedback control algorithm compared to those with “one dose fits all” (5% versus 19.8%, respectively) [165]. The role and utility of this model in real clinical practice is unknown.

According to well-documented data about the once-daily dosing of aminoglycoside antibiotics in decreasing the nephrotoxicity potential in certain clinical conditions, this approach has been also proposed and tested for polymyxin administration [127]. Despite achieving promising findings in some experimental studies [166], clinical investigations have failed to replicate this pattern. Paradoxically, a multicenter retrospective study has reported higher rates of AKI with once-daily over twice-daily dosing of polymyxin B [167]. Regarding possible nephroprotective effects of prolonging polymyxin durations of infusion, at least a retrospective record review conducted at a single center in Puerto Rico implicated that the risk of polymyxin B-associated AKI was comparable between intermittent and continuous infusion administration groups (41% vs. 31%, respectively, *p* = 0.4) [168].

#### 5.8.3. Avoiding or Reducing Modifiable Risk Factors

Whenever possible, the co-administration of other known nephrotoxic agents with polymyxins should be avoided. The potential benefit of combination therapy of polymyxins with other antibiotics only to achieve less nephrotoxicity is a matter of debate and is not currently suggested [127]. According to the results of a few retrospective and prospective studies in favor of comparable clinical and microbiological response rates between colistin and ampicillin–sulbactam in the treatment of carbapenem-resistant *Acinetobacter baumannii* infections [169,170], using alternative regimens or agents (e.g., ceftazidime/avibactam, ceftolozane/tazobactam, meropenem/vaborbactam, cefiderocol, imipenem/relebactam, and tigecycline) with much less nephrotoxicity potential can also be kept in mind by physicians in clinical practice [127,171]. Finally, although hypoalbuminemia has been demonstrated to be a risk factor of polymyxin AKI, hypoalbuminemia correction by administering exogenous albumin with the hope of preventing or mitigating nephrotoxicity has no clinical data so far and it is not advisable. In addition, currently, it is not also prudent to modify the daily dose of polymyxins in patients with hypoalbuminemia [127].

#### 5.8.4. Co-Administration of Nephroprotective Agents

Considering the pivotal role of oxidative stress and inflammation in the pathogenesis of polymyxin AKI, many agents with anti-oxidative stress, anti-inflammatory, and anti-apoptotic properties have been tested so far. This has also been the subject of some systematic reviews published recently [113,172], noting that most of the relevant studies have been performed in animal models. More noticeable evidence of efficacy in animal studies is reported with NAC, vitamin C, vitamin E, silymarin, and curcumin [112]. Related clinical investigations have been focused only on NAC, vitamin C, vitamin E, and melatonin. In this regard, two retrospective cohort studies showed that either IV NAC (300 mg two to three times a day) [173] or oral NAC (400–1200 mg/day) [174] did not prevent polymyxin-induced AKI based on changes in serum creatinine and GFR. Similarly, results of a randomized controlled clinical trial suggested that simultaneous administration of oral NAC with a dose of 1200 mg daily for 10 days has no significant effect in the prevention of colistin-induced AKI [175]. Intravenous administration of 2 g of ascorbic acid every 12 h also failed to show any significant benefits on kidney function or a reduction of urinary biomarkers of AKI (NGAL and NAG) caused by colistin [176]. In contrast, a prospective, observational cohort study of patients with septic shock or severe sepsis showed that the co-administration of IV vitamin C (2 g twice daily) may be effective in the prevention of colistin-induced AKI [177]. A randomized clinical trial showed no significant difference in the frequency as well as duration of AKI in patients receiving colistin or colistin plus vitamin E (400 units per day orally or by a nasogastric tube) [178]. Finally, the findings of a double-blind, randomized clinical trial in critically ill adults infected by multidrug-resistant bacteria suggested that the co-administration of 3 mg of oral melatonin per day until the end of colistin treatment had no significant impact on the rate of AKI, serum creatinine, BUN, and daily urine volume [179].

Collectively, considering the lack of conclusive clinical evidence along with possible pharmacokinetic/pharmacodynamic interactions, international consensus guidelines do not recommend the routine use of antioxidants for the prevention of polymyxin AKI [67].

**Table 7:** Summary of preventive strategies for polymyxin AKI based on clinical studies [127,170,180] *.

Strategy	Description
Using polymyxin B instead of colistin	There are inconclusive or controversial data, and it is generally not recommended.
Appropriate dose selection and modification	Consider dose adjustments based on: Kidney function (in patients with kidney impairment); Ideal body weight (in patients with obesity); Correct formulation of polymyxin (colistimethate sodium versus colistin base).
Avoid using loading dose	It is not recommended.
Once-daily dosing	There are inconclusive or controversial data, and it is not recommended.
Performing therapeutic drug monitoring	Although there is no standard analytical method and consensus about the procedure, performing TDM, wherever possible, is recommended, especially in the early treatment period.
Prolonging the duration of infusion	There are no clinical data, and it is not recommended.
Avoiding co-administered nephrotoxic agents	Whenever possible, all agents with nephrotoxic potential should be discontinued.
Using alternative regimens or medications	If the risk of AKI is estimated to be high, consider using less nephrotoxic agents (e.g., ampicillin–sulbactam, tigecycline, and eravacycline ± meropenem) instead of polymyxins.
Correcting hypoalbuminemia	There are no clinical data, and it is not recommended.
Co-administration of nephroprotective agents	N-acetylcysteine, oral (400–1200 mg/day) or intravenous (300 mg two to three times a day), was ineffective in preserving GFR. Intravenous vitamin C (2 g every 12 h) was ineffective in reducing the rate of AKI based on conventional and novel biomarkers of kidney function. Intravenous vitamin C (2 g [3–4 g] twice daily) was significantly effective in reducing the rate of AKI. Vitamin E (400 units oral per day) was ineffective in reducing the frequency and duration of AKI. Melatonin (3 mg of oral per day) had no significant impact on the rate of AKI, serum creatinine, BUN, and daily urine volume.

* Some selected references. For complete references, please refer to the relevant subsection.

### 5.9. Monitoring

Serum creatinine, BUN, and urine output should be measured at baseline and regularly during polymyxin treatment (Table 3) [144,181].

### 5.10. Treatment

Currently, there is no definitive treatment for polymyxin-induced AKI [36]. Supportive care is the mainstay of treatment. Adequate hydration, close monitoring of fluid intake and urine output, serum electrolytes, and the correction of fluid as well as electrolyte disorders, if necessary, are suggested. Although relevant data are inconclusive, discontinuing co-administered nephrotoxic agents, whenever possible, is advisable [36,69]. Intravenous administration of mannitol, exchange transfusions, and kidney replacement therapy may reduce the serum drug level. Hemofiltration and more specifically, hemoadsorption, can also rapidly remove colistin from systemic circulation; however, the definite role of these modalities and their standard procedures for the management of polymyxin-induced AKI in clinical practice is unclear. It is noteworthy that kidney replacement therapy such as hemodialysis is mostly indicated to alleviate complications of AKI associated with polymyxins [69]. There are no clinical data in favor of administering corticosteroids for the management of possible or documented interstitial nephritis caused by polymyxins.

Discontinuation of polymyxins and substituting them with other antibacterial agents/regimens with less nephrotoxicity potential (e.g., tigecycline, ampicillin–sulbactam, ceftolozane–tazobactam, ceftazidime–avibactam, meropenem–vaborbactam, and eravacycline ± meropenem) is a challenging strategy, but it appears to be inevitable in some conditions [36,180,182]. This clinical decision must be made cautiously, case by case, and after weighing the overall risks and benefits of continuation versus discontinuation of polymyxins. In the case of planning to continue treatment with polymyxins, appropriate dose reduction based on the estimated GFR of patients according to reliable charts is mandatory [36]. Currently, there is no standard protocol and consensus for performing any required dose adjustment, if necessary, based on polymyxins serum level in the setting of AKI.

## 6. Vancomycin

### 6.1. Introduction

Vancomycin is a glycopeptide antibiotic. It inhibits bacterial cell wall synthesis through binding to the D-alanyl-D-alanine portion of a cell wall precursor called peptidoglycan. Vancomycin has a broad activity against aerobic and anaerobic Gram-positive microorganisms including Streptococcus, Staphylococcus, Enterococcus (especially *Enterococcus faecalis*), Listeria, Peptostreptococcus, Actinomyces, Propionibacterium, and Clostridium [37]. Because of its long-time use, low cost, and optimal bactericidal effectiveness, vancomycin remains to be the antibiotic of choice for the treatment of suspected or known infections caused by methicillin-resistant *Staphylococcus aureus* (MRSA) or coagulase-negative staphylococci (CONS) in critically ill patients [37,183,184]. In this regard, up to 38% of patients during their ICU stay have been reported to receive vancomycin [185].

### 6.2. Epidemiology

Vancomycin-associated AKI was first identified and reported in 1958, at the same year of its approval for clinical use. The high frequency of AKI with traditional formulations of vancomycin has been attributed to its impurities. By the introduction of modern preparations of vancomycin with much higher purity levels, the incidence of AKI presently ranges from as low as 0% to more than 40% [186]. For example, a large study of 26,865 critically ill adult patients demonstrated that patients receiving vancomycin had higher rates of any stage AKI (hazard risk = 1.57, 95% CI = 1.41–1.76) and also AKI severity (hazard risk = 1.57, 95% CI = 1.36–1.82) [187]. The overall risk for vancomycin AKI appears to be lower in the pediatric population than that in adults, in particular premature infants [37]. In 15,724 young adults (age 16–25 years) hospitalized and treated in an ICU, the administration of certain medications such as vancomycin was independently associated with AKI in the overall cohort as well as each individual age group [188]. This is also the case in a retrospective cohort study of 226 critically ill children with an age between 1 month and 14 years [189]. According to a retrospective review on 50 critically ill adolescent and young adult patients aged between 15 to 25 years, 20 (40%) subjects developed vancomycin-associated AKI [190]. Finally, a systemic review and meta-analysis of 13 articles (6 observational cohort studies and 7 randomized clinical trials enrolling 4033 patients) found that vancomycin administration was associated with a 2.5-fold increase in the risk of AKI [191]. This wide variation in the frequency of vancomycin AKI is mostly related to the retrospective method and lack of control group in the majority of studies, different study settings (critically ill patients versus non-critically ill patients), the presence of risk factors (especially vancomycin trough levels, duration of treatment, and co-administered nephrotoxic medications), and disparity in the definition of AKI [184,186,192]. Therefore, vancomycin use alone has been associated with a relatively low rate of AKI in clinical studies in the absence of confounding factors [193].

### 6.3. Definition

Vancomycin-associated AKI is classically defined as an increase in serum creatinine concentration of 0.5 mg/dL or a ≥50% increase from the baseline, whichever is greater, occurring at least two different time points [186]. Another commonly used criterion for vancomycin-associated AKI is either a 0.5 mg/dL elevation in serum creatinine if the initial serum creatinine was ≤3 mg/dL or a rise of >1 mg/dL if the initial serum creatinine was >3 mg/dL [192]. Obviously, by the introduction of newer diagnostic criteria for AKI, most recent studies have exploited RIFLE, AKIN, and KDIGO for both diagnosis and assessment of vancomycin-associated AKI [184]. For example, a multicenter, retrospective, propensity score-matched cohort study of 1044 adult ICU patients compared the incidence of AKI associated with the combination of vancomycin plus piperacillin/tazobactam to the combination of vancomycin plus cefepime or vancomycin plus meropenem. Both serum creatinine only and serum creatinine with urine output-based KDIGO criteria were used in this investigation [193].

### 6.4. Pathophysiological Mechanisms

**Figure 6:**
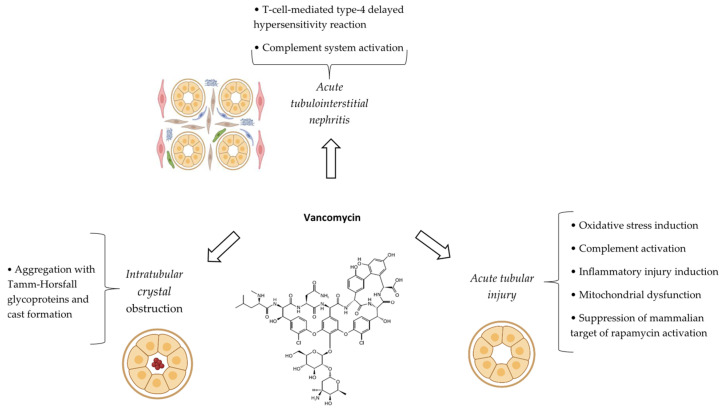
Summary of different aspects and pathophysiologic mechanisms of vancomycin nephrotoxicity [184,194,202].

Vancomycin nephrotoxicity has three identified pathological aspects including ATI, acute tubulointerstitial nephritis (ATIN), and intratubular crystal obstruction (Figure 6) [184]. Since limited kidney biopsies have been carried out in patients with vancomycin nephrotoxicity, the precise prevalence of these pathological manifestations is currently unknown. According to a review between 2010 and 2019 of 37 kidney biopsy specimens from patients treated with vancomycin who developed AKI in a hospital in the United States, the following findings were reported: 25 (67.6%) patients had both ATI and ATIN, 5 (13.5%) patients had ATI alone, 4 (10.8%) patients had interstitial fibrosis and tubular atrophy, and 3 (8.1%) patients had acute or chronic tubulointerstitial nephritis (TIN) alone [194]. A systematic review of case reports/case series published from 1989 to September 2020 about biopsy-proven vancomycin-induced AKI indicated that 10 (47.6%), 9 (42.9%), and 3 (14.3%) patients had ATI alone, ATIN alone, and both ATI and ATIN, respectively [195]. These findings have been confirmed by the other recently published comprehensive clinicopathologic studies [196].

The main mechanism of ATI is vancomycin accumulation in the proximal tubule epithelium (from the apical and basolateral surfaces) that induces an intracellular cascade through several pathways including oxidative stress, complement activation, inflammatory injury, and mitochondrial dysfunction, finally culminating in cellular apoptosis [184,186,197]. In more detail, the ROS induce lipid peroxidation of mitochondrial phospholipid cardiolipin [37]. This along with the permeabilization of lysosomes near the mitochondria and lysosomal membrane damage lead to mitochondrial membrane depolarization, mitochondrial membrane damage, cytochrome C release, caspase activation (caspases 9 and 3), and cell apoptosis [198,199]. In addition, vancomycin suppresses mammalian target of rapamycin activation. This subsequently can mediate massive autophagy in the proximal tubular epithelium [184].

The first biopsy-proven ATIN due to vancomycin was described in 1988 [200]. Although not completely understood yet, vancomycin-induced ATIN is attributed to T-cell-mediated type-4 delayed hypersensitivity reaction and, probably, complement system activation. Albeit ATIN is also classified as part of a type II antibody-mediated hypersensitivity reaction. In other words, both cell-mediated and humoral immunity can be involved in the process [199]. The altered expression of several components from the complement system, including complement component 3 (C3), complement component 4b (C4b), and C-X-C motif chemokine ligand 1 (Cxcl1), supports the involvement of complement system in the pathogenesis of ATIN by vancomycin [184]. A case of recurrent ATIN after secondary exposure to vancomycin can also be interpreted as an immunologic reaction [184].

In 2017, kidney tubular casts composed of vancomycin were first described to have an independent association with vancomycin nephrotoxicity. These tubular casts were confirmed by immunohistochemistry, immunoelectron microscopy, and infrared spectroscopy tests [201]. Surprisingly, reviewing pathologic findings of patients with vancomycin AKI revealed that in most of kidney specimens (89.3%), tubular casts were present [196]. Regarding possible pathogenesis, vancomycin aggregates and Tamm–Horsfall glycoproteins form the casts in the presence of a high urine vancomycin concentration and a low urine pH (<5.5) [198]. Crystallization ensues because of the vicious cycle of tubular obstruction and increased local vancomycin concentration. Cast formation mainly occurs in the distal tubules, which blocks urine flow there. This triggers inflammation and oxidative stress of the surrounding interstitium and, consequently, AKI [194,202].

### 6.5. Risk Factors

Potential risk factors that predispose patients to vancomycin nephrotoxicity can be classified as patient related, treatment related, and concurrent nephrotoxins (Table 8) [37,184,186,197]. Among patient-related factors, altered pharmacogenetics is a relatively new topic in the field of vancomycin nephrotoxicity. Polymorphisms of genes encoding kidney drug transporters and cytochrome P450 enzymes have been the subject of at least one clinical study [203].

Concomitant administration of nephrotoxic agents, especially aminoglycosides, can increase the risk of vancomycin AKI [37]. In addition, several recent studies have reported that the combination of vancomycin and piperacillin–tazobactam is associated with a significant increase serum creatinine. Accordingly, several systematic review and meta-analyses have also implied that the incidence of AKI with vancomycin plus piperacillin–tazobactam combination is much higher than that with vancomycin plus other antibiotic combinations [204,205]. Specifically, in critically ill adult patients, a systematic review and meta-analysis of 14 articles including 30,399 patients demonstrated that vancomycin and piperacillin–tazobactam combination is associated with a greater risk of AKI compared with vancomycin plus alternative agents for Gram-negative coverage (risk ratio: 1.79; 95% CI: 1.46–2.19; *p* < 0.001) [206]. In line with this, a recently published systematic review and network meta-analysis of 70 studies (comprising 76,638 patients) suggested that vancomycin and piperacillin/tazobactam significantly increased the risk of AKI and stages 2–3 AKI compared with vancomycin plus other anti-pseudomonal beta-lactams such as cefepime and meropenem [207]. Suggested mechanisms for this phenomenon are increased risk of ATI and more possibly, allergic acute interstitial nephritis. Besides proposed mechanisms, pseudo-AKI, mediated through the impairment of kidney proximal tubular creatinine secretion via inhibiting organic anion transporters 1 and 3 by piperacillin–tazobactam may also play a role in increased serum creatinine in recipients of vancomycin plus piperacillin–tazobactam combination [70,208]. This hypothesis has been supported by the findings of a prospective cohort study of 739 critically ill patients. Accordingly, vancomycin plus piperacillin–tazobactam recipients had significantly higher rates of AKI compared to those received vancomycin plus cefepime combination based on the creatinine definition. In contrast, alternative biomarkers including serum cystatin C and BUN were comparable between two groups. In addition, renal replacement therapy or mortality at 30 days did not differ significantly [209].

In the context of drug-related factors, the results of several studies on critically ill patients are in favor of a significant association between vancomycin serum trough level (greater than 15 or 20 mg/L) and the risk of AKI [37,186]. For example, a large study of 26,865 critically ill adult patients has reported that a serum trough level greater than 20 mg/L was associated with AKI (HR = 1.90, 95% CI = 1.52–2.30) and stage 2/3 based on the KDIGO criteria (HR = 1.69, 95% CI = 1.31–2.19) [187]. Apart from the risk of AKI, vancomycin trough concentrations 15–20 and >20 mg/L have a significant association with both increased ICU and in-hospital mortality in critically ill patients [210]. In addition to trough level, an interventional clinical study of 53 critically ill adult patients demonstrated that vancomycin peak levels, measured after the end of 1 h infusion of the fifth dose, were significantly associated with AKI [211]. The area under the concentration–time curve (AUC) of vancomycin also contributes to AKI in critically ill patients [76]. Accordingly, a meta-analysis of eight observational studies with a total of 2491 patients suggested that a vancomycin AUC24 less than 650 mg·h/L was associated with a decreased risk of AKI [212]. This cut-off value has been also reported in at least two more recent studies [213,214].

Regarding total daily dose, some available data have suggested that vancomycin doses of >4 g/day can increase the incidence of AKI [186]. Treatment with vancomycin for more than one week can increase the frequency of AKI from 6% to 21%. If the duration of vancomycin treatment exceeds 2 weeks, the incidence of AKI may reach up to 30% [197]. Intermittent intravenous infusion of vancomycin, as the main administration route of vancomycin, has been associated with a higher risk of AKI compared to continuous intravenous infusion [186]. In contrast to the total daily dose and duration of treatment, administering a vancomycin loading dose (25–30 mg/kg) in critically ill patients was associated with achieving optimal therapeutic and AUC/MIC targets without increasing the risk of AKI [186,215]. Interestingly, at least a systematic review and meta-analysis have demonstrated that the risk of AKI in patients who received a loading dose of vancomycin was even lower than the control group [216].

### 6.6. Clinical/Paraclinical Manifestations and Outcomes

AKI often develops 4.3–17 days after initiating vancomycin therapy [37]. In this regard, a recently published cross-sectional study of both ICU and non-ICU patients reported the mean ± SD and minimum–maximum time interval between vancomycin initiation and AKI was 5.44 ± 2.04 days and 3–13 days, respectively (Figure 2) [153]. However, vancomycin-associated AKI with time onsets as early as 2 to 3 days of therapy has also been reported [186].

Preliminary clinical studies have reported an increase in baseline serum creatinine of about 1–1.5 mg/dL within the course of vancomycin-associated AKI [186]. Accordingly, the mean increase in peak serum creatinine values from baseline ranged from 0.85 mg/dL to 1.4 mg/dL; this corresponds to a 35–45% decrease in creatinine clearance from baseline values [217]. Based on a more recent case-control study of 22 patients received vancomycin, the mean ± SD peak serum creatinine in those who developed vancomycin AKI was 3.7 ± 1.4 mg/dL [218]. In terms of ATIN due to vancomycin, urinalysis can show hematuria, pyuria, eosinophiluria, proteinuria, and white blood cell casts on dipstick testing. The classic triad of fever, rash, and eosinophilia suggestive of drug-induced interstitial nephritis is present only in a minority of patients in real clinical condition (>5–10%). Vancomycin-induced ATIN is not an exception from this rule [199]. In the setting of crystal nephropathy, vancomycin casts have variable appearances such as periodic acid–Schiff red, granular aggregates to spherules mixed with Tamm–Horsfall protein, and necrotic epithelial cells [202]. Having a meticulous approach toward clinical manifestations, laboratory findings, and temporal relationship can assist in differentiating vancomycin-associated AKI from other causative factors such as sepsis. For example, vancomycin casts in the urine confirmed by immunohistochemistry have almost a pathognomonic appearance [196].

A vancomycin-associated increase in serum creatinine usually resolves after prompt discontinuation, especially in the case of cast nephropathy [185]. In contrast, the risk of permanent kidney dysfunction in cases of ATIN alone is five-fold higher than ATI alone [195]. In general, vancomycin-associated AKI will either improve or resolve in up to three-quarters of patients by the time of discharge, often within a week or less (Figure 2) [197,217]. According to a retrospective observational study of patients aged 80 years and older, the median time to clinical resolution from vancomycin nephrotoxicity was 5 days (range, 4 to 11 days) [219]. However, some patients, especially those with critical illness, may not experience full kidney function resolution [184]. In this regard, a retrospective, observational cohort study of 415 patients with acute bacterial skin/skin structure infections demonstrated that those with vancomycin nephrotoxicity compared to those without nephrotoxicity had a significantly longer hospital length of stay (9 vs. 6 days, *p* = 0.001), higher 30-day readmission rates (30.8% vs. 9.0%, *p* < 0.001), and increased all-cause 30-day mortality (5.1% vs. 0.3%, *p* = 0.024) [220]. In the setting of ICUs, at least a retrospective, multicenter observational study of 188 adults with hospital-acquired pneumonia, ventilator-associated pneumonia, or healthcare-associated pneumonia demonstrated that patients who developed vancomycin AKI had significantly longer median ICU and hospital stays (17 and 20 days, respectively) compared to those without AKI (12 and 15 days, respectively) [221].

### 6.7. Biomarkers

Assessing novel biomarkers of kidney function in plasma and blood in the setting of vancomycin nephrotoxicity have been the subject of many both animal and human studies. Some of these biomarkers in human studies include urine KIM-1, urine NGAL, urine TIMP2-IGFBP7, serum cystatin C, serum osteopontin, serum trefoil factor-3, serum 5-hydroxy indole acetic acid/serotonin ratio, and serum tumor necrosis factor receptor 1 [3,42,162,184,186,197]. For example, secondary analysis of a prospective, multicenter, Sapphire study enrolling 333 critically ill adult patients receiving vancomycin alone, piperacillin–tazobactam alone, or vancomycin plus piperacillin–tazobactam combination demonstrated that in patients with stages 2–3 AKI, urinary TIMP2-IGFBP7 was significantly elevated at the day of vancomycin initiation, peaking at the following day; while, serum creatinine was significantly elevated and peaked one day after starting the therapy [222]. The results of another prospective study of 87 patients who received vancomycin, of whom 32 (36.8%) were in the ICU, implicated that urinary KIM-1 and NGAL levels were significantly higher on days 0, 1, 2, and 3 in the vancomycin-associated AKI group compared to the non-AKI group. In addition, these two biomarkers could discriminate patients with or without vancomycin AKI better than serum creatinine. Of note, the efficiency of combining these two biomarkers was not substantially improved compared to each biomarker alone [223]. Besides these biomarkers, the up-regulation of inflammatory proteins in urine, including complement C3, complement C4, galectin-3-binding protein, fibrinogen, alpha-2 macroglobulin, immunoglobulin heavy constant mu, and serotransferrin, after vancomycin AKI has been also described in 10 patients [218].

### 6.8. Prevention

In clinical practice, several strategies can be considered to prevent or minimize vancomycin AKI (Table 9).

#### 6.8.1. Risk Stratification

Several risk prediction models for vancomycin-induced AKI have been designed and used in the Japanese population [224,225]. The most recent one is a user-friendly flowchart (a decision-tree type), which comprises three splitting variables including co-administered vasopressors, co-administered piperacillin–tazobactam, and body mass index ≥ 30 kg/m^2^. It categorized vancomycin-induced AKI into low-risk (<10%), intermediate-risk (10–25%), and high-risk groups (>25%). The accuracy of the flowchart was determined to be appropriate (86.4%) [226]. Besides a flowchart, a nomogram has been also constructed and validated to detect the risk of vancomycin-associated AKI in critical care patients [227].

Overall, if the risk of AKI is estimated to be high, it seems prudent to substitute vancomycin with other less nephrotoxic alternative agents, used for the treatment of MRSA infection, including teicoplanin (not available in the United States), linezolid, daptomycin, tigecycline, telavancin, and ceftaroline [37,184].

#### 6.8.2. Optimizing Modifiable Risk Factors

Patients with volume depletion should receive adequate hydration both before and during vancomycin treatment [37]. Whenever possible, concomitant use of other potentially nephrotoxic agents, especially aminoglycosides and piperacillin–tazobactam, with vancomycin should be avoided. A possible alternative agent to piperacillin–tazobactam that can be used in clinical practice is cefepime. If using these combinations is inevitable, the minimum required duration of treatment along with meticulous, regular kidney function assessment and TDM should be considered [71]. According to the relationship between indexes of vancomycin exposure (trough concentration and duration of therapy) and its AKI, the duration of vancomycin therapy must be limited as much as possible, preferably less than 7 days [184]. Notably, the type as well as severity of infection and patient response to treatment are factors that should be considered in determining the duration of treatment with vancomycin.

Vancomycin trough levels should be closely monitored, and the dose should be adjusted, if necessary, in all critically ill patients [73,184]. The aim of this practice is maintaining vancomycin target trough levels between 10 and 20 mcg/mL for adults or 5 and 15 mcg/mL for pediatric patients/neonates and an AUC/MIC ratio between 400 and 650 (by assuming a vancomycin MIC of 1 mg/L) for serious MRSA infections [73]. At least a systematic review and meta-analysis implicated that pharmacokinetics-informed dosing of vancomycin reduces the risk of AKI without compromising efficacy [228]. In patients with obesity, the vancomycin dose should be calculated based on ABW [73]. In addition, the total daily dose of vancomycin usually should not exceed 3600 mg/day and 4500 mg/day for most pediatric and adult patients with obesity, respectively, unless justified by its serum concentration. Finally, regarding the vancomycin loading dose in adults, it should be no more than 3000 mg [229].

#### 6.8.3. Method of Administration as Continuous Infusion

The rationale behind this approach originates from the idea and relevant clinical findings that high peak vancomycin concentrations are linked with AKI. Continuous infusion allows for minimizing the peak concentration of vancomycin. This technique of vancomycin administration deems to be beneficial from both safety and clinical efficacy point of view. It may allow for achieving target concentrations faster with less variability and requiring less TDM [37,184]. A systematic review and meta-analysis of 11 studies, involving 2123 critically ill adult patients, found that continuous infusion was associated with a 53% lower risk of AKI compared to the intermittent infusion strategy [230]. Nevertheless, this meta-analysis has limitations like low number of patients in the two enrolled randomized control trials and variations in the empiric dosing protocol and adjustment strategies [230]. On the other hand, there are technical considerations of continuous infusion (e.g., availability of intravenous access). Therefore, this method of vancomycin administration should be selected only after assessing all clinical and paraclinical aspects of the patients [37].

#### 6.8.4. Co-Administration of Nephroprotective Agents

Up to now, the beneficial effects of various chemical or herbal medications on the prevention of vancomycin AKI have been demonstrated in preclinical studies. These agents include erdosteine, vitamin E, vitamin C, N-acetylcysteine, caffeic acid, phenethyl ester, erythropoietin, cilastatin, fosfomycin, melatonin, zingerone, rutin, naringenin, saffron, silymarin, and dexmedetomidine [198,231,232]. The justification of using nephroprotective antioxidants mostly relies on oxidative stress being one of the major mechanisms of vancomycin nephrotoxicity [233]. The nephroprotective effects of cilastatin and fosfomycin in these studies were generally attributed to modulating tubular reabsorption and the secretion of vancomycin, leading to decreased cellular accumulation in proximal tubules [231].

Regarding clinical data, an observational and retrospective case-control study implicated the lower, but not statistically significant, incidence of AKI with vancomycin + vitamin C (500 mg twice daily for two doses) compared to vancomycin alone [234]. In line with this study, continuous intravenous infusion of high-dose vitamin C (50–200 mg/kg/d based on disease severity) in a retrospective study significantly improved kidney function indexes (serum creatinine and BUN) and shortened the length of ICU stay compared to the control group [235]. Similarly, the results of a randomized controlled clinical trial suggested that concurrent oral NAC (600 mg every 12 h for 10 days) significantly decreased serum creatinine and increased creatinine clearance in vancomycin recipients. Nevertheless, the rate of vancomycin-induced AKI was comparable between NAC and control groups (4.76% and 12.63%, respectively; *p* = 0.066) [236]. Nephroprotective effects against vancomycin AKI have been also reported with the concomitant administration of oral vitamin E (400 units per day for 10 days) in a matched-groups interventional study characterized by creatinine clearance and AKI [237]. According to a retrospective cohort study, the concurrent use of oral melatonin (3 mg or 5 mg per day) during vancomycin treatment was associated with a 63% reduction in AKI [238]. Finally, a double-blinded, placebo-controlled, pilot clinical trial demonstrated that the co-administration of 140 mg silymarin tablet orally three times daily significantly prevented vancomycin AKI (both incidence and severity). These nephroprotective effects were achieved partially by enhancing the antioxidant system including serum total antioxidant capacity as well as glutathione and decreasing serum malondialdehyde [239].

Despite these promising experimental and clinical findings along with appropriate safety, cost, and availability, routine use of these agents in clinical practice appears to be premature and does not stand to reason [232].

**Table 9:** Summary of suggested strategies for preventing vancomycin AKI [184,226,230,232] *.

Strategy	Description
Calculating appropriate dose	The vancomycin dose should be calculated based on actual body weight (even in patients with obesity), preferably not exceeding 3600 mg/day in pediatric patients and 4500 mg/day in adults with obesity.
Monitoring serum level	The trough level and AUC/MIC ratio (preferably) of vancomycin should be closely monitored in all critically ill patients. The vancomycin dose should be adjusted to achieve trough levels of 10–20 mcg/mL for adults or 5–15 mcg/mL for pediatric patients/neonates and an AUC/MIC ratio between 400 and 650 mg·h/L.
Limiting the duration of treatment	The duration of vancomycin therapy must be limited, preferably less than 7 days.
Prolonging the duration of infusion	Administering vancomycin as a continuous infusion (24 h) may be beneficial in selected cases.
Avoiding the co-administration of nephrotoxic agents	Whenever possible, concomitant use of potentially nephrotoxic agents, especially aminoglycosides and piperacillin–tazobactam, should be avoided. If these combinations are inevitable, the minimum required duration of treatment along with regular kidney function assessment and TDM should be considered.
Using less nephrotoxic alternative agents	If the risk of AKI is estimated to be high, consider using other less nephrotoxic agents instead of vancomycin such as teicoplanin (not available in the United States), linezolid, daptomycin, tigecycline, telavancin, or ceftaroline.
Co-administration of nephroprotective agents	Vitamin C (500 mg twice daily for two doses) may be partially effective. Continuous infusion of high-dose vitamin C (50–200 mg/kg/d) was significantly effective in improving kidney function indexes and shortening the length of ICU stay. Oral n-acetyl cysteine (600 mg every 12 h for 10 days) was significantly effective in preserving creatinine clearance. Oral vitamin E (400 units per day for 10 days) was significantly effective in decreasing the rate of AKI and also preserving creatinine clearance. Oral melatonin (3 mg or 5 mg per day) was significantly effective in reducing the rate of AKI. Oral silymarin (140 mg three times a day) was significantly effective in reducing both the incidence and severity of AKI.

* Some selected references. For complete references, please refer to the relevant subsection.

### 6.9. Monitoring

In all settings, classical indexes of kidney function including serum creatinine and BUN should be monitored daily. Table 3 has listed the monitoring indexes/suggestions about vancomycin AKI.

### 6.10. Treatment

Like most cases of DIKD, there is currently no specific and conclusive therapy for most aspects of vancomycin AKI including ATI and crystal nephropathy [37]. The only exception may be biopsy-proven ATIN. In this regard, several case series have attempted oral steroids (prednisone, 1 mg/kg/day for about four weeks) to accelerate the rate of resolution from vancomycin-induced ATIN with inconclusive findings [240,241]. Therefore, steroid therapy has not an established role for this purpose. Collectively, the management of vancomycin AKI mostly relies on either dose de-escalation or discontinuing the causative agent along with supportive therapy [37,242].

Besides these modalities, hemodialysis might also be useful in removing vancomycin and improving kidney outcome of affected patients, especially pediatric patients. Since vancomycin is a large glycopeptide compound, conventional (low-flux) hemodialysis membranes cannot adequately remove this agent from plasma. In this regard, about 7% of vancomycin can be removed during a typical 4 h conventional dialysis run. On the other hand, high-flux hemodialysis filters such as polysulphone, polynitrile, and polymethylmethacrylate can remove vancomycin from plasma more effectively (about 20–40%). According to the fact that plasma vancomycin concentration can rebound 3–6 h after a hemodialysis session, frequent hemodialysis may be necessary to achieve optimal therapeutic results [37,229]. The vancomycin serum concentration should also be checked before each dialysis session [229].

## 7. Aminoglycosides

### 7.1. Introduction

Aminoglycosides are among the oldest antibiotics that have been used to treat serious infections caused by Gram-negative and some Gram-positive bacteria [243]. They exert their antibacterial actions by interacting with the 30S subunit of ribosome and inhibiting protein synthesis [244]. Aminoglycosides have concentration-dependent bactericidal activity along with prolonged post-antibiotic effects [245]. Currently, the main use of these drugs in critically ill patients is the treatment of infections caused by nosocomial Gram-negative bacilli (e.g., *Pseudomonas aeruginosa*, *Acinetobacter pneumoniae*, *Klebsiella pneumoniae*, and Carbapenemase [KPC]-producing *Klebsiella pneumoniae*) [243,246].

Despite their benefits including fast bactericidal effect, synergy with β-lactam antibiotics (for the treatment of staphylococcal or enterococcal bacteremia and endocarditis), low incidence of resistance, and relatively low cost, the utility of aminoglycosides has a sharp downward trend in the late 1970s [245]. Their use in US academic centers has continued to decline by 41% from 2002 to 2009 [244]. This event is mostly due to the introduction of broad-spectrum and better-tolerated antibiotics including fluoroquinolones, carbapenems, third- as well as fourth-generation cephalosporins, and broad-spectrum β-lactam/β-lactamase inhibitor combinations [245]. In other words, the major Achilles’ heel of aminoglycosides in current clinical practice is their adverse effects, most notably ototoxicity and nephrotoxicity [78].

### 7.2. Epidemiology

Aminoglycoside-associated AKI occurs in 10% to 25% of aminoglycoside recipients [14]. Most reports have been in the range of 5% to 15% [244]. AKI ranges between 29% and 58% in critically ill patients [247]. In contrast, according to several prospective, randomized studies in seriously ill patients, only 5% to 10% of aminoglycoside recipients experienced substantive decrement in GFR [244]. This wide variability in the incidence of aminoglycoside-associated AKI has been attributed to disparities in the operational criteria, the type as well as frequency of testing to measure kidney function, nephrotoxicity potentials of different agents in the class, and the clinical setting of the study population [14,244].

### 7.3. Definition

Generally, aminoglycoside-associated AKI is defined as either a rise in the plasma creatinine concentration of more than 0.5 to 1 mg/dL or a 50 percent increase in the plasma creatinine concentration from the baseline value [248]. Apart from this, newer definitions have been applied for aminoglycoside AKI. For example, a multicenter, prospective, descriptive cohort study recruiting 1001 adult patients in ten ICUs in France exploited the KDIGO criteria for determining AKI caused by prescribed nephrotoxic medications such as aminoglycosides [249]. A pharmacoepidemiologic evaluation in 3865 critically ill children identified gentamicin to be significantly associated with AKI as defined by the RIFLE criteria [250].

### 7.4. Pathophysiological Mechanisms

**Figure 7:**
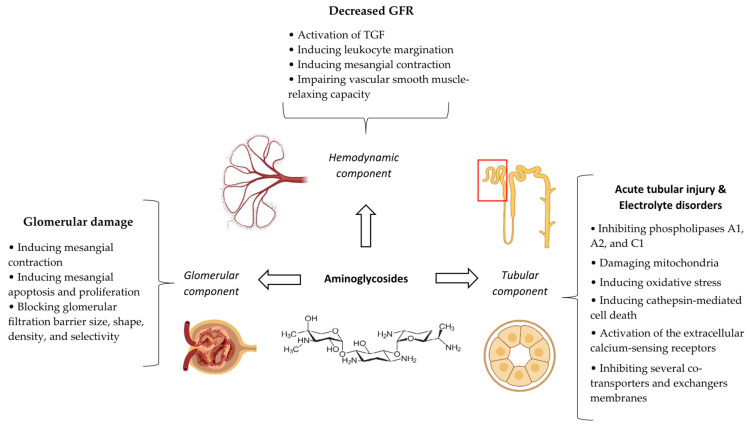
Summary of different aspects and pathophysiologic mechanisms of aminoglycoside nephrotoxicity [14,251].

It appears that aminoglycosides exert their nephrotoxic effects on both tubules and glomerulus. In addition, they can also reduce kidney blood flow. In other words, aminoglycoside nephrotoxicity has hemodynamic, tubular, and glomerular components (Figure 7) [251].

Traditionally, tubular toxicity and ATI are considered as primary mechanisms of aminoglycoside-associated AKI [14]. Aminoglycosides can accumulate, mainly in the proximal tubule and partly in the distal tubule, as well as collecting ducts, by endocytosis [251]. This process is mediated via giant endocytic complex encompassing megalin and cubilin. Following endocytosis, aminoglycosides concentrate in lysosomes, the Golgi body, and endoplasmic reticulum [198]. When the aminoglycoside concentration in these intracellular organelles exceeds an undetermined threshold, the drug is poured into the cytosol [245]. After that, aminoglycosides inhibit phospholipases A1, A2, and C1, leading to phospholipidosis [198]. Aminoglycosides can damage mitochondria both directly and indirectly, thus impairing ATP production, culminating in cell death [251]. Oxidative stress via increasing superoxide anions and hydroxyl radicals also have a role in tubular injury. Apoptosis of tubular cells is mediated through several intracellular pathways such as cathepsins and calcium-sensing receptors (CaSRs) [251]. Aminoglycoside-induced electrolyte disorders have been mostly attributed to inhibiting Na-Pi cotransporter, Na-H exchanger, and Na-K ATPase activity both in the apical and basolateral membranes of tubular cells [198].

Regarding glomerular aspects, aminoglycosides can induce mesangial contraction. Apart from mesangial contraction, they can also stimulate both the apoptosis and proliferation of mesangial cells. Several pathways and related mediators as well as enzymes have been identified or suggested to describe these events in the glomerulus. They include platelet-activating factor, endothelin-1, thromboxane A2, reactive oxygen species, CaSR, calcium, inducible nitric oxide synthase, and the renal renin–angiotensin–aldosterone system. The size, shape, and density of the filtration barrier of glomeruli can be altered by aminoglycosides through neutrophil infiltration. Finally, aminoglycosides can selectively block glomerular filtration barrier selectivity through cationic charge groups in their chemical structure. This can neutralize the negative charge of the glomerulus [251].

A decrease in the GFR during aminoglycoside exposure is partially because of the effects of these medications on the kidney vasculature. The decrease in kidney blood flow is mostly induced by the activation of TGF. In turn, TGF is mostly the consequence of damage to the proximal tubule by aminoglycosides. Endothelin-1, PAF, and arachidonic acid metabolites, mainly prostaglandins and thromboxane A2, are major mediators of vasoconstriction in this setting. Apart from TGF, aminoglycosides can impair vascular smooth muscle-relaxing capacity through the inhibition of phospholipase C, protein kinase C, and calcium movements. Eventually, it has been speculated that aminoglycosides can plug the kidney vascular system by inducing leukocyte margination [251].

### 7.5. Risk Factors

Several factors increased the risk of aminoglycoside AKI. They can be classified and viewed as (1) the aminoglycoside type, dosing, and method of administration, (2) concomitant drugs, and (3) patient demographic, clinical/paraclinical characteristics, and concomitant diseases (Table 10) [14,78,244,248,251].

The number of cationic groups on the aminoglycoside molecule appears to correlate with the degree of injury. In this regard, some prospective comparative trials have demonstrated that neomycin has the greatest nephrotoxic potential, followed by gentamicin, tobramycin, amikacin, and netilmicin in decreasing order [14]. Since streptomycin has the least affinity for the proximal tubule cells’ binding sites, it is deemed to have the least nephrotoxicity [252]. In line with these findings, a meta-analysis of 43 randomized trials in both adults and children demonstrated less AKI with tobramycin compared to gentamicin [253].

Although aminoglycoside AKI occurs in a dose-dependent manner, there is no conclusive relationship between its trough levels and risk of AKI. Nevertheless, gentamicin and tobramycin trough serum concentrations more than 2 mg/mL and amikacin trough level more than 10 mg/mL have been reported to increase the risk of AKI [78]. For example, in critically ill patients with severe sepsis or septic shock, it has been implicated that serum trough values (range) of amikacin were significantly higher in patients with AKI (10.8 [5.1 to 15.5] mg/L) compared to those without AKI (1.8 [0.6 to 4.7] mg/L) [254].

Considering the saturable feature of kidney tubular accumulation of aminoglycosides, conventional multiple dosing has a higher AKI potential relative to once-daily dosing [248]. Prolonged duration of treatment can increase the risk of toxic aminoglycoside concentrations, leading to enhanced AKI. It is defined as ≥3 days [244], more than 10 days [255], or 14 days [256].

Like other DIKDs, the co-administration of potentially nephrotoxic medications is expected to increase the risk of aminoglycoside-associated AKI. Furosemide, vancomycin, and amphotericin B are only some examples [244]. Although initial studies suggested that cephalothin can increase the AKI risk of aminoglycosides, this phenomenon has not been observed with other cephalosporins [248]. An increased risk of AKI with concomitant piperacillin has also been reported. In contrast, carbenicillin or ticarcillin co-administration may be protective against aminoglycoside AKI [244]. The possible mechanism and definite clinical relevance for these findings have not been elucidated yet.

In terms of patient characteristics, older age, pre-existing kidney or liver disease, shock, and hospitalization in an ICU are some major relevant examples [14,244]. Although the female sex was identified as a risk factor of aminoglycoside in one study, this finding has not been confirmed in others [244]. Volume depletion and hypotension, especially in patients with septic shock or sepsis syndrome, are associated with the increased risk of aminoglycoside-induced AKI. This is partially attributed to the release of endotoxins that may have role in enhanced drug accumulation in the tubules. Similarly, reduced effective arterial volume, which occurs in the setting of heart failure and advanced liver disease, is another relevant risk factor. Apart from increasing the risk of aminoglycoside AKI, kidney ischemia in the above conditions can, in turn, accelerate the time of onset of developing this event [248].

In the setting of critically ill patients, few clinical studies have focused on risk factors of aminoglycoside AKI. For example, in a surgical ICU in Brazil, diabetes, hypotension, the use of iodinated contrast, and the simultaneous use of other nephrotoxic drugs (NSAIDs, vancomycin, cephalothin, amphotericin B, cisplatin, cyclosporine, and tacrolimus) were identified as independent risk factors of aminoglycoside AKI (amikacin or gentamicin). In contrast, a calculated GFR < 60 mL/min/1.73 m^2^ was determined to be an independent protective factor against aminoglycoside-induced AKI (OR = 0.42; 95% CI = 0.24–0.72; *p* = 0.02). The authors justified this latter finding that in case of underlying moderate kidney disease, the ICU team may usually prefer to prescribe lower doses of aminoglycoside. This may protect the tubular cells against high systemic exposure and, consequently, a high luminal concentration of antibiotic [247]. Another study of 61 patients in a surgical ICU in the United States was reported concurrent use of vasopressors and vancomycin as independent predictors of aminoglycoside-associated AKI [257].

### 7.6. Clinical/Paraclinical Manifestations and Outcomes

Aminoglycoside-associated AKI usually occurs 5 to 7 days after initiating the treatment (Figure 2) [14], albeit later onset of aminoglycoside-induced AKI (e.g., 10 days) has also been reported [87]. It is typically characterized by a gradual but progressive rise in serum creatinine as well as BUN along with a decrease in creatinine clearance [14]. The increase in the plasma creatinine is generally mild (0.5 to 2.0 mg/dL) [248]. Affected patients are usually non-oliguric, in whom the urine volumes are expected to be greater than 500 mL/day [14]. Nevertheless, in the case of distal tubular dysfunction, polyuria is likely [248]. Regarding urine analysis findings, microscopic hematuria, mild proteinuria, pyuria, hyaline as well as granular casts, and low specific gravity can be detected. Urine sodium concentration is generally more than 40 mEq/L, and the fractional excretion of sodium is estimated to be above 1 percent [14,79,244,248].

Electrolyte abnormalities and Bartter-like syndrome including hypomagnesemia, hypokalemia, hypocalcemia, and hypophosphatemia are infrequently observed. Although magnesium wasting can occur (defined by daily excretion of more than 10–30 mg), the risk of symptomatic hypomagnesemia is generally low [14,79]. Fanconi-like syndrome, which manifests with glycosuria, aminoaciduria, phosphaturia and uricosuria, can also occur [248]. Despite these paraclinical findings, the diagnosis of aminoglycoside-induced AKI in critically ill patients is often challenging. This is due to the presence of multiple comorbidities and possible confounders such as dehydration, hypotension, sepsis, ischemia, and the use of other nephrotoxic drugs [14]. In this regard, paying attention to potentially reversible etiologies (e.g., transient hypovolemia and urinary tract obstruction) and reviewing all possible imaging procedures as well as medications for any nephrotoxic agent (like iodinated contrast media, furosemide, vancomycin, and amphotericin B) seem crucial [14].

Most cases of aminoglycoside-associated AKI resolve [244]. Accordingly, after cessation of aminoglycoside treatment, the serum creatinine level usually returns to its baseline value within 21 days (Figure 2). Kidney function resolution can be delayed if the patient remains hypovolemic, septic, or catabolic [248]. Severe AKI may also develop, which requires kidney replacement therapy. For example, a study of ICU patients with sepsis receiving amikacin demonstrated that 6 out of 15 patients (40%) who developed AKI eventually underwent kidney replacement therapy [254]. In another retrospective review in surgical ICU patients, 12.9% of patients required dialysis following aminoglycoside associated AKI [257]. Regarding mortality, a retrospective cohort analysis of 360 patients under aminoglycoside treatment in a clinical-surgical ICU implicated that in-hospital mortality rate was significantly higher in the AKI (44.5%) compared to non-AKI group (29.1%) [247].

### 7.7. Biomarkers

Among novel biomarkers of kidney function, most clinical studies about aminoglycoside AKI have been focused on urinary KIM-1, urinary beta-2 microglobulin, serum as well as urinary cystatin C, urinary monocyte chemoattractant protein-1, urinary IL-18, urinary NGAL, urinary NAG, and urinary TIMP2-IGFBP7 [3,42,78,161,162]. These studies have been done predominantly in pediatric and non-critically ill patients. There are only few relevant investigations in the ICU setting. For example, a proof-of-concept study of premature neonates hospitalized in a neonatal ICU receiving gentamicin demonstrated that urinary KIM-1, NGAL, and NAG levels normalized to urinary creatinine concentration had significant associations with AKI. However, by using multivariate analysis, only urinary KIM-1 elevation remained significantly associated with gentamicin-associated AKI [258].

Besides these biomarkers, microRNAs also appear to have an association with aminoglycoside AKI. In this regard, at least four animal studies were published about gentamicin AKI by February 2020 [259]. For example, miR-155, which participates in the regulation of inflammation, has been demonstrated to increase significantly in the kidney tissue in the setting of gentamicin AKI in a rat model [259]. Other experimental studies have also reported significant associations between the urinary expression of miR-138, miR-1971, miR-218, miR-489, miR-26, miR-192, and miR-378 and gentamicin-induced AKI [260,261].

### 7.8. Prevention

In clinical practice, several strategies have been suggested to prevent or minimize aminoglycoside AKI. Furthermore, there are also some novel approaches currently under investigation (Table 11).

#### 7.8.1. Choice of Aminoglycoside

If possible, the least toxic aminoglycoside should be selected [252]. Notably, the type of infection and causative pathogens should also be considered in selecting the most appropriate aminoglycoside. For example, in the case empirical or definite treatment of infections caused by *P. aeruginosa*, amikacin or tobramycin appear to be relatively more appropriate aminoglycosides [262]. Notably, the difference in nephrotoxic potential between different agents within this class in real clinical practice is overall small and not profound [248].

#### 7.8.2. Alternative Antibiotics

Fluoroquinolones (e.g., ciprofloxacin or levofloxacin) and third- or fourth-generation cephalosporins (e.g., ceftazidime or cefepime) are some examples of antibiotics with much more appropriate kidney safety profiles that can be considered in clinical practice instead of aminoglycosides [14]. These alternatives are mostly preferred in patients with volume depletion, hypotension, or reduced effective arterial volume [252].

#### 7.8.3. Modifiable Risk Factors

If pre-existing hypokalemia, hypomagnesemia, or hypovolemia is present, it should be corrected or optimized before starting an aminoglycoside [252]. If possible both clinically and bacteriologically, the duration of treatment with aminoglycosides should be limited to less than 7–10 days. If the estimated duration of treatment is expected to be more than 14 days, some experts recommend against starting aminoglycosides [78]. It is also advisable to minimize concomitant nephrotoxic medications (e.g., vancomycin and amphotericin B) whenever possible [14].

Administering aminoglycosides as high-dose intermittent dosing, also known as once-daily dosing, has been intensively investigated with different aminoglycosides (mostly tobramycin) in various clinical conditions (mostly cystic fibrosis) [263]. Although at least comparable clinical efficacy and reduced AKI have been documented with once-daily compared with multiple dosing, this approach of aminoglycoside administration appears not to be a suitable option for most critically ill patients, especially those with pre-existing kidney disease or severe ascites [14]. This is because of altered aminoglycoside pharmacokinetics in this population [77].

The administration of aminoglycosides via nebulization (especially tobramycin) has been investigated widely in children with cystic fibrosis. It has been expected that nebulized tobramycin may be associated with less AKI compared to the intravenous route due to its less systemic bioavailability [264]. Moreover, a systematic review and meta-analysis of randomized controlled trials on amikacin nebulization for the adjunctive therapy of Gram-negative pneumonia in mechanically ventilated patients (13 randomized clinical trials comprising 1733 adults) exhibited that amikacin nebulization did not add significant nephrotoxicity compared to the control group [265].

#### 7.8.4. Therapeutic Drug Monitoring and Dose Calculation

Although the clear link between aminoglycoside levels and its AKI has not been demonstrated yet, performing TDM appears to be helpful. There is no standard guideline for the optimal TDM of aminoglycosides in either a multiple-daily or once-daily dosing regimen. Nevertheless, several relevant tables and nomograms have been published. Target peak and trough levels depend on the site as well as severity of infection, type of causative bacteria, type of aminoglycoside, and the method of aminoglycoside administration (multiple-daily dosing versus once-daily dosing) [77].

Regarding the dosing weight of aminoglycosides, TBW and IBW are suggested for calculating required both loading and maintenance (in particular) doses for underweight and normal weight/overweight, respectively. In the case of patients with obesity, considering the adjusted body weight (calculated as IBW + [0.4 × (TBW − IBW)]), rather than TBW, seems necessary to avoid possible supratherapeutic levels of aminoglycoside and minimize AKI [77,266].

According to the recommendation of the Acute Dialysis Quality Initiative (ADQI), the early prediction of moderate to severe AKI using artificial intelligence may have a favorable impact on clinical outcomes [267]. There are over 300 models published with reasonable performance at predicting AKI onset, AKI severity, and post-AKI complications in hospitalized adult and pediatric populations [267]. Interestingly, in patients receiving aminoglycoside treatment and having a 90% risk of developing stages 2/3 (moderate to severe) AKI within the next 48 h, reviewing the dose and indication of medications along with checking the serum level were displayed and noted to their caregivers in this model. The model successfully predicted 70% of subsequent RRT episodes, 58% of stage 2/3 episodes, and 41% of any AKI episodes [268]. The satisfactory application of machine learning algorithm for predicting nephrotoxic AKI in the context of high-nephrotoxin exposure (e.g., receiving intravenous aminoglycoside for ≥3 days) in hospitalized adults has been also reported [269].

#### 7.8.5. Co-Administering Nephroprotective Agents

At both preclinical and clinical levels, many synthetic chemical drugs and natural products have been investigated to determine their possible functions in preventing aminoglycoside AKI. These studies have been the subject of at least one systematic review and one meta-analysis published so far [270,271]. Among studied nephroprotectants in experimental studies, herbs and natural agents have shown protection against aminoglycoside AKI. Nephroprotection of these agents is mostly attributed to their antioxidant, anti-inflammatory, and anti-apoptotic activities [271]. Alpha-lipoic acid, vitamin E, vitamin C, desferrioxamine, methimazole, selenium, melatonin, N-acetylcysteine, dimethyl-sulfoxide, and pioglitazone are some relevant examples [14,252].

In contrast to preclinical level, related clinical data are quite limited. Results from a small randomized clinical trial in 32 patients receiving gentamicin for the treatment of an upper urinary tract infection demonstrated an improvement in creatinine clearance in the nifedipine group (10 mg three times a day) compared to the placebo group. These findings can be interpreted as either the interference of the calcium channel blocker with the specific pathophysiological mechanisms of aminoglycoside AKI or exerting direct vasodilatory effects of this agent on afferent arteriole. The latter mechanism is independent to nephroprotective activities of nifedipine [272]. In the case of statins, as the potential inhibitor of megalin in proximal tubules, two randomized, controlled clinical trials failed to show any significant reduction of aminoglycoside AKI including amikacin and tobramycin by the co-administration of either atorvastatin (40 mg once daily) in critically ill adults [273,274] or rosuvastatin (10 mg once daily) in children with cystic fibrosis [275]. The studied parameters and endpoints in these clinical trials were AKI, GFR, conventional as well as novel biomarkers of kidney function (e.g., urine NGAL/urine Cr and KIM-1), and electrolytes imbalances. In contrast, the findings of two other double-blinded, randomized clinical trials in non-critically ill patients with infectious diseases have demonstrated that silymarin (140 mg three times a day) [276] and pentoxifylline (400 mg three times a day) [277] co-administration for 7 days were significantly associated with a lesser overall rate of gentamicin AKI compared to the placebo group. In the latter study, a significant decrease in serum levels of MDA and TNF-α in pentoxifylline recipients compared to the placebo group suggests that the antioxidant and anti-inflammatory properties of this agent exert nephroprotective effects against gentamicin [277].

#### 7.8.6. Aminoglycoside Congeners, New Derivatives, and Novel Formulations

Designing, synthesizing, isolating, and preparing aminoglycoside congeners, derivatives, and novel formulations with less cellular toxicity is a relatively novel and ongoing field of research in aminoglycoside AKI.

Commercially available gentamicin in the pharmaceutical market is not a homogeneous compound; rather, it is a mixture of C1, C1a, C2, and C2a congeners. Interestingly, the C2 compound, as the specific congener of gentamicin, has been shown to be bactericidal with less risk of AKI. A potential mechanism for explaining this observation is that C2 is a poor substrate of megalin in proximal tubule cells, and it accumulates to a lesser extent in the Golgi apparatus and lysosomes [252]. The major challenge of using these congeners in clinical practice is the possibility of decreased bactericidal activity and, consequently, an increased risk of treatment failure [278].

In the case of aminoglycosides derivatives, plazomicin is one of the newest semisynthetic agents of this class [244]. It has two additions to the parental sisomicin skeleton at its N-1 (hydroxyl-aminobutyric acid) and N-6′ (hydroxyethyl) positions. Preliminary studies in humans have revealed modest AKI (7% of patients), but no ototoxicity with this agent [279]. In addition, an integrated analysis of three randomized, controlled trials showed that the rates of plazomicin- and comparator-associated AKI were 4.8% and 4.1%, respectively. This difference did not reach the level of statistical significance. The discontinuation of plazomicin because of AKI occurred only in three patients [280].

Novel formulations such as nanostructured aminoglycosides can be prepared by incorporating these agents into different nanoparticle carriers including liposomes, lipids, carbohydrates, gold, silver, and silicon. The potential advantages of these novel formulations are their improved pharmacodynamics and pharmacokinetic properties [281]. In this regard, for example, gentamicin loaded on nanostructured lipid carriers containing tocopheryl polyethylene glycol 1000 succinate surfactant has demonstrated significantly less AKI compared to controls in rabbits [282]. Similarly, amikacin and gamma amino butyric acid combination loaded on chitosan nanoparticles had renoprotective activities in rats [283].

#### 7.8.7. Time of Dosing

Aminoglycosides, especially tobramycin and gentamicin, have an established diurnal rhythm about their pharmacokinetic and pharmacodynamic behaviors [264]. For example, in children with cystic fibrosis under tobramycin treatment, the urinary KIM-1 level was significantly higher in patients who received tobramycin in the evening (8:00 p.m.) compared to those who received this agent in the morning (8:00 a.m.) [284]. Similarly, in 179 patients with severe infections treated with gentamicin or tobramycin, the rate of AKI from midnight to 7:30 a.m., 8:00 a.m. to 3:30 p.m., and 00 p.m. to 11:30 p.m. was 34.6%, 12.5%, and 9.3%, respectively [285]. Lower urinary pH during rest and low food intake in the evening may account for these findings. Accordingly, it has been demonstrated that the likelihood of interaction between aminoglycosides and anionic phospholipids, as one of the major mechanisms of aminoglycoside nephrotoxicity, is higher when the urine pH is low [79].

**Table 11:** Summary of studied strategies for preventing aminoglycoside nephrotoxicity [14,241,248,271] *.

Strategy	Description
Calculating appropriate dose	Considering total, ideal, and adjusted body weights for calculating required doses for patients underweight, with normal weight/overweight, and with obesity, respectively.
Monitoring serum level	Trough level of aminoglycosides should be closely monitored in most cases. Gentamicin/tobramycin and amikacin doses should be adjusted to avoid serum trough concentrations more than 2 mg/mL and 10 mg/mL, respectively.
Using the aminoglycoside with less nephrotoxicity potential	In the case of empirical or definite treatment of infections caused by *P. aeruginosa*, amikacin or tobramycin appear to be relatively more appropriate aminoglycosides.
Using alternative antibiotics with less nephrotoxicity	If the risk of AKI is estimated to be high, fluoroquinolones and third- or fourth-generation cephalosporins can be used instead of aminoglycosides.
Limiting the duration of treatment	If possible, the duration of treatment with aminoglycosides should be limited to less than 7–10 days. If the expected duration of treatment is more than 10 days, some experts recommend not starting aminoglycosides.
Avoiding the co-administration of nephrotoxic agents	Whenever possible, the co-administration of nephrotoxic agents (e.g., vancomycin and amphotericin B) should be avoided.
Administering as once-daily dosing	In certain and selected clinical conditions, aminoglycosides can be given as once-daily dosing (versus multiple-daily dosing).
Co-administration of nephroprotective agents	Oral nifedipine (10 mg three times a day) was effective in improving creatinine clearance. Oral atorvastatin (40 mg once daily) was ineffective in reducing AKI and changes in conventional as well as novel biomarkers of kidney function. Oral rosuvastatin (10 mg once daily) was ineffective in preventing changes in GFR and conventional as well as novel biomarkers of kidney function. Oral silymarin (140 mg three times a day) was significantly effective in reducing AKI and preserving creatinine clearance. Oral pentoxifylline (400 mg three times a day) was significantly effective in reducing AKI and preserving creatinine clearance.
Using aminoglycoside congeners, new derivatives, and novel formulations	Plazomicin appears to be less nephrotoxic and better tolerated compared to other aminoglycosides. The C2 congener of gentamicin has less potential nephrotoxicity and also bactericidal activity. There are no clinical data about the kidney safety of novel formulations such as nanostructured aminoglycosides.
Administering aminoglycosides in the morning	Administering aminoglycosides in the morning may be associated with less nephrotoxicity compared to that given in the evening.

* Some selected references. For complete references, please refer to the relevant subsection.

### 7.9. Monitoring

The serum creatinine and BUN concentrations should be measured at baseline and also every one to three days, depending upon the individual clinical/paraclinical conditions [77,78]. If serum creatinine increases more than 50 percent over baseline values, urinalysis and urine output should also be determined [77]. Table 3 provides more details about the monitoring of aminoglycosides.

### 7.10. Treatment

The management of aminoglycoside AKI is basically supportive [79]. It mostly relies on discontinuing the culprit drug and substituting it by another non- or less nephrotoxic antibiotic (e.g., third/fourth-generation cephalosporins or fluoroquinolones) [14]. If this measure is not clinically feasible, the dose of aminoglycoside should be corrected to achieve targeted levels. According to the fact that aminoglycosides have a concentration-dependent antibacterial activity, increasing the time interval between doses, rather than decreasing each dose, seems more appropriate from a pharmacodynamics point of view [79]. Patients with aminoglycoside AKI should be kept adequately hydrated, and any possible electrolyte disorders should be corrected. Short-term kidney replacement therapy may also be necessary [14].

## 8. Conclusions

Nephrotoxicity is a common and dose-limiting adverse effect of AmB, cidofovir, foscarnet, polymyxins, vancomycin, and aminoglycosides. Anti-infective-associated AKI is generally more frequent in critically ill patients compared to non-critically ill. There is no standard definition or operational criteria for AKI with anti-infectives; although, AKIN, RIFLE, and KDIGO are more commonly used definitions in recent clinical studies. Overall, different criteria for DIKD should not be applied for different drugs. Anti-infective-induced AKI usually occurs during the first two weeks of treatment. It is mostly dose dependent, with an anticipated clinical resolution based on serum creatinine returning to baseline. However, kidney injury can affect a patient’s renal functional reserve and provide susceptibility to future AKI events. DIKD, especially in the ICU population, can adversely affect clinical outcome indexes such as mortality and length of stay.

Different pathophysiological mechanisms impacting glomerular, tubular, and interstitial components of the kidney are usually responsible for the development of nephrotoxicity with anti-infective medications. Among them, ATI is generally more prominent and predominant. Oxidative stress and inflammation play a pivotal role in the pathogenesis of DIKD including anti-infectives. Numerous patient-related, medication-related, and co-administered-medication variables have been demonstrated as risk factors of anti-infective-induced AKI. Some of these risk factors (like co-administered nephrotoxic medications [e.g., loop diuretics and NSAIDs] and the duration of treatment [e.g., more than 1 week]) are usually modifiable and can be optimized to prevent or minimize AKI. Apart from traditional indexes of kidney function (serum creatinine and urine output), novel biomarkers of kidney function (especially serum cystatin C) and damage (mostly urinary KIM-1 and NGAL) have been noticed in recent clinical studies with promising findings. Nevertheless, the diagnosis of AKI caused by anti-infectives in critically ill patients is often challenging due to the presence of multiple comorbidities and possible confounders. Having a multidisciplinary approach including considering casual/temporal relationships, complete history taking as well as physical examination, and performing all required laboratory tests and even renal biopsy are imperative to rule out possible differential diagnoses and confirming DIKD.

The efficiency of preventive strategies against anti-infective-induced AKI in most cases appears to variable, relative, and modest. Hydration is the major preventive modality for AmB, cidofovir, and foscarnet AKI. Despite having some promising clinical findings, nephroprotective agents like antioxidants are not currently recommended for the prevention of anti-infective-induced AKI. Close and regular monitoring of kidney function parameters is crucial during treatment with nephrotoxic anti-infectives. Moreover, in all recipients of vancomycin or aminoglycosides in the critically ill setting, performing TDM is necessary. Currently, there are no definitive treatment modalities for the management of AKI with anti-infectives. Therefore, supportive care is the mainstay of treatment.

## 9. Future Directions

Future studies should address the current knowledge gaps in the field of antibiotic-induced AKI. Accordingly, evaluating the kidney safety of antibiotic formulations based on novel delivery systems in the clinical setting is suggested. For instance, lipid conjugates, micelles, nanoemulsions, cubosomes, liposomes, ethosomes, niosomes, and lipid-polymer hybrid nanoparticles are only some relevant examples of nanotechnology-based formulations of AmB with almost no documented data about their AKI in humans. The definite and direct role of TDM and modifying the administration method (like prolonging the duration of infusion or once-daily dosing) in prevention/attenuating AKI caused by medications such as vancomycin, aminoglycosides, and polymyxins should be determined. The real status and impact of using artificial intelligence techniques and novel kidney biomarkers as part of a multidisciplinary approach related to nephrotoxin stewardship of antibiotics also deserve further attention.

## Figures and Tables

**Table 1:** Suggested risk factors of amphotericin B AKI [13,14,21,26,27,28,29] *.

Patient Related	Medication Related	Co-Administered Medications
Male gender	High average daily dose (e.g., more than 60 mg)	Cyclosporine
Older age	High cumulative dose (e.g., more than 600 mg or 2–5 g)	Foscarnet
Weight equal or more than 90 kg	High serum levels (e.g., more than 1.0 mg/L)	Cidofovir
Baseline kidney dysfunction	Rapid infusion	Pentamidine
		Ganciclovir/Valganciclovir
Dehydration		Furosemide
Mechanical ventilation		
ICU admission		Angiotensin-converting enzyme inhibitors/Angiotensin receptor blockers
		Vasopressors
		Vancomycin
		Aminoglycosides
		Carbapenems
		Cisplatin
		Ifosfamide

* Some selected references. For complete references, please refer to the relevant subsection.

**Table 3:** Monitoring parameters of kidney function and possible nephrotoxicity during treatment with amphotericin B, cidofovir, foscarnet, polymyxins, vancomycin, and aminoglycosides.

Medication	Monitoring Parameters/Suggestions
Amphotericin B	Serum creatinine and BUN concentrations should be checked daily [14]. Serum magnesium, potassium, and calcium concentrations should also be monitored every other day [14].
Cidofovir	Urine protein levels along with serum creatinine should be measured within 24 to 48 h prior to the administration of each dose in induction and maintenance phases [62].
Foscarnet	If possible, 24 h creatinine clearance should be measured at baseline. During the induction and maintenance phases of foscarnet treatment, serum creatinine and electrolytes (Ca^2+^, Mg^2+^, K^+^, and phosphate) should be determined twice weekly and once weekly, respectively [63,64]. Some references have advised that serum creatinine should be monitored daily within the initial phase of treatment during hospitalization. Sustained changes in the serum creatinine level (e.g., at least 0.4 mg/dL or more on 2 subsequent occasions) generally require foscarnet dose adjustment [65]. Creatinine clearance (mL/min/kg) should also be calculated by using the Cockcroft–Gault equation, even if serum creatinine is within the normal range [66]. Checking hydration status before and after each course of foscarnet infusion is also recommended [64]. Note that normal total calcium measurements may not reflect ionized hypocalcemia [39].
Polymyxins	International consensus guidelines are in favor of performing TDM for both colistin and polymyxin B, wherever possible, especially in the early treatment period [67]. In the steady state, average target trough levels of about 2 mg/L and 2–4 mg/L have been proposed for colistin and polymyxin B, respectively [68]. These target trough levels correspond to AUC_ss24 h_ values of about 50 mg h/L and 50–100 mg h/L, respectively [67]. The lack of a standard analytical method of measurement and difficulties in sample collection, handling, and analysis of sodium colistimethate (as a prodrug of colistin) have currently challenged routine TDM of polymyxins in real clinical practice [68,69].
Vancomycin	In the case of vancomycin combination therapy with piperacillin–tazobactam, monitoring novel functional/damage kidney biomarkers (e.g., serum cystatin C and urine NGAL), urine output, and calculated GFR has been also suggested by some researchers to rule out possible pseudo-AKI [70,71]. Performing urinalysis including microscopic sediment examination is also advised to assess the likelihood of ATIN [71]. In patients with uncomplicated skin and soft tissue infections who do not have obesity and have normal kidney function, monitoring vancomycin serum trough is generally not needed [72]. Performing TDM is recommended in all critically ill patients or those with obesity, burns, impaired kidney function, individuals who receive concomitant nephrotoxic agents, and those with impaired kidney function. If TDM is required, it should be conducted 48 h and 72 h after the initiation of therapy in patients with normal and those with impaired kidney function, respectively. Time intervals of repeating TDM are mostly dependent to the method of vancomycin administration (intermittent versus continuous infusion), hemodynamic status of the patient, and also duration of treatment. The vancomycin trough level should be monitored more frequently if serum creatinine increases during the course of treatment [73]. Considering the possibility of significant interpatient variability in the vancomycin level, the most current 2020 guidelines are in favor of using Bayesian-derived AUC monitoring rather than trough concentrations. Findings of recent studies have also supported the role of AUC-based TDM in reducing AKI incidence and the total daily dosage of vancomycin in clinical practice [74,75]. Due to several practical issues such as requiring a higher number of patient samples as well as an application/software to calculate the AUC, more trained personnel, and also potential errors in accurate sampling times, many centers still use therapeutic trough levels [76].
Aminoglycosides	In the case of prophylactic therapy for less than 24 h, the measurement of aminoglycoside concentrations is not necessary [77]. If the duration of aminoglycoside empiric treatment is less than 72 h, performing TDM is generally deemed unnecessary [78]. In patients received aminoglycosides with traditional multiple-daily dosing for therapeutic purposes, serum concentrations should be measured after giving two to three maintenance doses or after adjustment of the dose. Trough concentrations are measured within 30 min or immediately (preferably) before the next dose and peak concentrations 30 to 45 min after the end of an intravenous infusion or approximately 60 min after an intramuscular injection [77,79]. In patients received extended-interval dosing, the serum drug concentration is usually measured after the first dose. By achieving the desired peak and trough serum concentrations, serum aminoglycoside concentrations should be re-evaluated only in the case of any changes in kidney function or treatment duration beyond 7 to 10 days in either once-daily or multiple-daily dosing regimens [77].

**Table 4:** Summary of suggested strategies for preventing cidofovir nephrotoxicity [34,62,86] *.

Strategy	Description
Hydration	One liter of normal saline 1 h before infusing each dose of cidofovir; if the patient tolerates this, a second liter will also be given.
Co-administration of probenecid	2 g two hours before and 1 g two hours and also eight hours after each infusion for a total of 4 g.
Alternative formulations of cidofovir	Using brincidofovir with less nephrotoxicity potential instead of cidofovir may be effective.
Avoiding the co-administration of nephrotoxic agents	Discontinuing NSAIDs, contrast media, aminoglycosides, AmB, foscarnet, and pentamidine at least 7 days before starting cidofovir.
Avoiding cidofovir administration in patients with underlying kidney disease	Not starting cidofovir in patients with: Baseline serum creatinine more than 1.5 mg/dL Calculated creatinine clearance less than 55 mL/min Underlying significant proteinuria (≥100 mg/dL or 2+)

* Some selected references. For complete references, please refer to the relevant subsection.

**Table 5:** Summary of suggested strategies for preventing foscarnet nephrotoxicity [63,66,100,109] *.

Strategy	Description
Intravenous hydration	First infusion: 750 to 1000 mL of normal saline (preferably) or 5% dextrose given intravenously immediately before the infusion. Subsequent infusions: 500 to 1000 mL of normal saline (preferably) or 5% dextrose given intravenously immediately before the infusion.
Oral hydration	Sixteen ounces (450 mL) of any type of oral fluids (preferably non-alcohol and non-caffeine) to achieve a urine output of 200 mL.
Continuous infusion	Administering foscarnet as a continuous infusion (24 h) appears to have no superiority over intermittent infusion (at least 1 to 2 h).
Avoiding in patients with moderate-to-severe underlying kidney disease	Not starting foscarnet in patients with: Baseline serum creatinine levels greater than 2.8 mg/dL Measured 24 h creatinine clearances < 50 mL/min

* Some selected references. For complete references, please refer to the relevant subsection.

**Table 6:** Suggested risk factors of polymyxin nephrotoxicity [36,69,116,123,127] *.

Patient Related	Medication Related	Co-Administered Medications
Higher age	High daily dose (e.g., more than 5.0 mg/kg/day for colistin and equal or more than 200 mg for polymyxin B)	Loop diuretics
Higher weight	High trough level (e.g., more than 4 μg/mL for colistin)	Calcineurin inhibitors
Chronic comorbid conditions (e.g., diabetes and pre-existing kidney disease)	High area under the concentration–time curve 24 h at the steady state (e.g., more than 100 mg h/L for polymyxin B)	Nonsteroidal anti-inflammatory drugs
Severity of illness (e.g., higher APACHE-II scores)		Intravenous contrast media
Septic shock		Glycopeptides
Hypoalbuminemia		Aminoglycosides
Hyperbilirubinemia		Vasopressors
		Proton pump inhibitor
		Rifampin (possibly)

* Some selected references. For complete references, please refer to the relevant subsection.

**Table 8:** Suggested risk factors of vancomycin nephrotoxicity [37,184,186,197] *.

Patient Related	Medication Related	Co-Administered Medications
Older age	Serum trough level greater than 20 mg/L	Aminoglycosides
Female gender	Serum peak level	Piperacillin–tazobactam
Obesity (e.g., total body weight > 91 kg or 101.4 kg)	AUC equal or more than 650 mg·h/L	Loop diuretics
Pre-existing comorbidities: Diabetes Heart failure Underlying kidney disease (creatinine clearance < 60 mL/min/m^2^, nephrotic syndrome) Underlying liver disease (advanced liver cirrhosis and obstructive jaundice) Immunosuppression	Daily doses more than 4 g	Amphotericin b
Severity of current illness: Higher APACHE II score Higher Charlson Comorbidity Index scores Septic shock Hypotension requiring treatment with vasopressors	Treatment duration more than 1 week	Acyclovir
Race	Intermittent intravenous infusion (versus continuous infusion)	Calcineurin inhibitors
Pharmacogenetics		Intravenous contrast media
		Vasopressors
		Renin–angiotensin system blockers

* Some selected references. For complete references, please refer to the relevant subsection.

**Table 10:** Suggested risk factors of aminoglycoside AKI [14,78,244,248,251] *.

Patient Related	Medication Related	Co-Administered Medications
Older age	Type of aminoglycoside: Neomycin > gentamicin > tobramycin > amikacin > netilmicin > streptomycin	Vancomycin
Pregnancy		
Pre-existing kidney disease	Serum concentrations more than 2 mg/mL (gentamicin and tobramycin) or more than 10 mg/mL (amikacin)	Amphotericin B
		Foscarnet
Pre-existing liver disease	Multiple-daily dosing	Cephalotin
Obstructive jaundice	Prolonged duration of treatment (e.g., >10 days)	Piperacillin
Hypoalbuminemia		Clindamycin
Diabetes		Cyclosporine and tacrolimus
Leukemia		Loop diuretics
Hypothyroidism		Iodinated radiographic contrast agents
Volume depletion and hypotension		Cisplatin
Shock		NSAIDs
Sepsis		Loop diuretics
Metabolic acidosis		
Potassium or magnesium deficiencies		
ICU hospitalization		

* Some selected references. For complete references, please refer to the relevant subsection.

## Data Availability

Not applicable.

## References

[1] Weisbord S.D., Palevsky P.M., Chertow G., Luyckx V., Marsden P., Skorecki K., Taal M., Alan Y. (2020). Prevention and management of acute kidney injury. Brenner and Rector’s the Kidney.

[2] Dasta J.F., Kane-Gill S. (2019). Review of the Literature on the Costs Associated With Acute Kidney Injury. J. Pharm. Pract..

[3] Kane-Gill S.L., Smithburger P.L., Kashani K., Kellum J.A., Frazee E. (2017). Clinical Relevance and Predictive Value of Damage Biomarkers of Drug-Induced Kidney Injury. Drug Saf..

[4] Mehta R.L., Awdishu L., Davenport A., Murray P.T., Macedo E., Cerda J., Chakaravarthi R., Holden A.L., Goldstein S.L. (2015). Phenotype standardization for drug-induced kidney disease. Kidney Int..

[5] Dennen P., Douglas I.S., Anderson R. (2010). Acute kidney injury in the intensive care unit: An update and primer for the intensivist. Crit. Care Med..

[6] Stottlemyer B.A., Tran T., Suh K., Kane-Gill S.L. (2025). A Systematic Review of the Costs of Drug-Associated Acute Kidney Injury and Potential Cost Savings With Nephrotoxin Stewardship Prevention Strategies. Clin. Pharmacol. Ther..

[7] Gray M.P., Barreto E.F., Schreier D.J., Kellum J.A., Suh K., Kashani K.B., Rule A.D., Kane-Gill S.L. (2022). Consensus Obtained for the Nephrotoxic Potential of 167 Drugs in Adult Critically Ill Patients Using a Modified Delphi Method. Drug Saf..

[8] Goswami E., Ogden R.K., Bennett W.E., Goldstein S.L., Hackbarth R., Somers M.J.G., Yonekawa K., Misurac J. (2019). Evidence-based development of a nephrotoxic medication list to screen for acute kidney injury risk in hospitalized children. Am. J. Health Syst. Pharm..

[9] Fernández-Llaneza D., Vos R.M.P., Lieverse J.E., Gosselt H.R., Kane-Gill S.L., van Gelder T., Klopotowska J.E., LEAPfROG Consortium (2025). An Integrated Approach for Representing Knowledge on the Potential of Drugs to Cause Acute Kidney Injury. Drug Saf..

[10] Faustino C., Pinheiro L. (2020). Lipid Systems for the Delivery of Amphotericin B in Antifungal Therapy. Pharmaceutics.

[11] Drew R.H. Pharmacology of Amphotericin B. UpToDate 2025. https://www.uptodate.com/contents/pharmacology-of-amphotericin-b.

[12] Cavassin F.B., Baú-Carneiro J.L., Vilas-Boas R.R., Queiroz-Telles F. (2021). Sixty years of Amphotericin B: An Overview of the Main Antifungal Agent Used to Treat Invasive Fungal Infections. Infect. Dis. Ther..

[13] Karimzadeh I., Farsaei S., Khalili H., Dashti-Khavidaki S. (2012). Are salt loading and prolonging infusion period effective in prevention of amphotericin B-induced nephrotoxicity?. Expert Opin. Drug Saf..

[14] Nolin T.D., Perazella M.A., DiPiro J.T., Yee G.C., Posey L.M., Haines S.T., Nolin T.D., Ellingrod V. (2023). Drug-induced kidney disease. Pharmacotherapy: A Pathophysiologic Approach.

[15] Karimzadeh I., Khalili H., Farsaei S., Dashti-Khavidaki S., Sagheb M.M. (2013). Role of diuretics and lipid formulations in the prevention of amphotericin B-induced nephrotoxicity. Eur. J. Clin. Pharmacol..

[16] Mistro S., Maciel I.M., de Menezes R.G., Maia Z.P., Schooley R.T., Badaró R. (2012). Does lipid emulsion reduce amphotericin B nephrotoxicity? A systematic review and meta-analysis. Clin. Infect. Dis..

[17] Smith C.L., Bondi D.S., Bridgeman M.M., Burke J.M. (2021). Infectious Diseases II. Updates in Therapeutics^®^: Pharmacotherapy Preparatory Review and Recertification Course.

[18] Harbarth S., Pestotnik S.L., Lloyd J.F., Burke J.P., Samore M.H. (2001). The epidemiology of nephrotoxicity associated with conventional amphotericin B therapy. Am. J. Med..

[19] Goldman R.D., Koren G. (2004). Amphotericin B nephrotoxicity in children. J. Pediatr. Hematol. Oncol..

[20] Awdishu L., Mehta R.L. (2017). The 6R’s of drug induced nephrotoxicity. BMC Nephrol..

[21] Abdel-Hafez Y., Siaj H., Janajri M., Abu-Baker Y., Nazzal Z., Hamdan Z., Adwan R., Aiesh B.M., Anaya A.I. (2022). Tolerability and epidemiology of nephrotoxicity associated with conventional amphotericin B therapy: A retrospective study in tertiary care centers in Palestine. BMC Nephrol..

[22] Perazella M.A. (2025). Amphotericin B Nephrotoxicity. UpToDate. https://www.uptodate.com/contents/amphotericin-b-nephrotoxicity.

[23] Downes K.J., Hayes M., Fitzgerald J.C., Pais G.M., Liu J., Zane N.R., Goldstein S.L., Scheetz M.H., Zuppa A.F. (2020). Mechanisms of antimicrobial-induced nephrotoxicity in children. J. Antimicrob. Chemother..

[24] Smogorzewski M.J., Rude R.K., Yu A.S.L., Chertow G., Luyckx V., Marsden P., Skorecki K., Taal M., Alan Y. (2020). Disorders of calcium, magnesium, and phosphate balance. Brenner and Rector’s the Kidney.

[25] DuBose T.D., Chertow G., Luyckx V., Marsden P., Skorecki K., Taal M., Alan Y. (2020). Disorders of acid-base balance. Brenner and Rector’s the Kidney.

[26] John J., Loo A., Mazur S., Walsh T.J. (2019). Therapeutic drug monitoring of systemic antifungal agents: A pragmatic approach for adult and pediatric patients. Expert Opin. Drug Metab. Toxicol..

[27] Takazono T., Tashiro M., Ota Y., Obata Y., Wakamura T., Miyazaki T., Nishino T., Izumikawa K. (2020). Factor analysis of acute kidney injury in patients administered liposomal amphotericin B in a real-world clinical setting in Japan. Sci. Rep..

[28] Garcia J.A., Guglielmo B.J., Zeind C.S., Carvalho M.G. (2023). Principles of Infectious Diseases. Applied Therapeutics: The Clinical Use of Drugs.

[29] Gursoy V., Ozkalemkas F., Ozkocaman V., Serenli Yegen Z., Ethem Pinar I., Ener B., Akalın H., Kazak E., Ali R., Ersoy A. (2021). Conventional Amphotericin B Associated Nephrotoxicity in Patients With Hematologic Malignancies. Cureus.

[30] Personett H.A., Kayhart B.M., Barreto E.F., Tosh P., Dierkhising R., Mara K., Leung N. (2019). Renal Recovery following Liposomal Amphotericin B-Induced Nephrotoxicity. Int. J. Nephrol..

[31] Ullmann A.J. (2008). Nephrotoxicity in the setting of invasive fungal diseases. Mycoses.

[32] Aoki F.Y., Bennett J.E., Dolin R., Blaser M.J. (2020). Antivirals against herpesviruses. Mandell, Douglas, and Bennett’s Principles and Practice of Infectious Diseases.

[33] Izzedine H., Launay-Vacher V., Deray G. (2005). Antiviral drug-induced nephrotoxicity. Am. J. Kidney Dis..

[34] (2000). Vistide (Cidofovir) Package Insert.

[35] Rodriguez M., Zachary K.C. (2025). Foscarnet: An Overview. UpToDate. https://www.uptodate.com/contents/foscarnet-an-overview.

[36] Pike M., Saltiel E. (2014). Colistin- and polymyxin-induced nephrotoxicity: Focus on literature utilizing the RIFLE classification scheme of acute kidney injury. J. Pharm. Pract..

[37] Murray B.E., Arias C.A., Nannini E.C., Bennett J.E., Dolin R., Blaser M.J. (2020). Glycopeptides (Vancomycin and Teicoplanin) and Lipoglycopeptides (Telavancin, Oritavancin, and Dalbavancin). Mandell, Douglas, and Bennett’s Principles and Practice of Infectious Diseases.

[38] Wazny L.D., Brophy D.F. (2000). Amiloride for the prevention of amphotericin B-induced hypokalemia and hypomagnesemia. Ann. Pharmacother..

[39] Chan J.C., Santos F., Hand M., Chertow G., Luyckx V., Marsden P., Skorecki K., Taal M., Alan Y. (2020). Fluid, electrolyte, and acid-base disorders in children. Brenner and Rector’s the Kidney.

[40] Ferguson M.A., Vaidya V.S., Bonventre J.V. (2008). Biomarkers of nephrotoxic acute kidney injury. Toxicology.

[41] McDuffie J.E., Lee S., Ma J.Y., Chen Y., Snook S. (2016). Acute biomarker panel changes associated with amphotericin B nephrotoxicity in female Sprague-Dawley rats. J. Toxicol. Sci..

[42] Griffin B.R., Faubel S., Edelstein C.L. (2019). Biomarkers of Drug-Induced Kidney Toxicity. Ther. Drug Monit..

[43] Da Y., Akalya K., Murali T., Vathsala A., Tan C.S., Low S., Lim H.N., Teo B.W., Lau T., Ong L. (2019). Serial Quantification of Urinary Protein Biomarkers to Predict Drug-induced Acute Kidney Injury. Curr. Drug Metab..

[44] Karimzadeh I., Heydari M., Ramzi M., Sagheb M.M., Zomorodian K. (2017). Urinary Neutrophil Gelatinase-associated Lipocalin as a Biomarker of Kidney Injury in Hematologic-Oncologic Patients Receiving Amphotericin B. Iran. J. Kidney Dis..

[45] Rocha P.N., Macedo M.N., Kobayashi C.D., Moreno L., Guimarães L.H., Machado P.R., Badaró R., Carvalho E.M., Glesby M.J. (2015). Role of urine neutrophil gelatinase-associated lipocalin in the early diagnosis of amphotericin B-induced acute kidney injury. Antimicrob. Agents. Chemother..

[46] Stevens D.A., Bennett J.E., Dolin R., Blaser M.J. (2020). Antifungal agents: Amphotericin B. Mandell, Douglas, and Bennett’s Principles and Practice of Infectious Diseases.

[47] Armstrong-James D., Koh M., Ostermann M., Cockwell P. (2020). Optimal management of acute kidney injury in critically ill patients with invasive fungal infections being treated with liposomal amphotericin B. BMJ Case Rep..

[48] Tonin F.S., Steimbach L.M., Borba H.H., Sanches A.C., Wiens A., Pontarolo R., Fernandez-Llimos F. (2017). Efficacy and safety of amphotericin B formulations: A network meta-analysis and a multicriteria decision analysis. J. Pharm. Pharmacol..

[49] Eriksson U., Seifert B., Schaffner A. (2001). Comparison of effects of amphotericin B deoxycholate infused over 4 or 24 hours: Randomized controlled trial. BMJ.

[50] Falagas M.E., Karageorgopoulos D.E., Tansarli G.S. (2013). Continuous versus conventional infusion of amphotericin B deoxycholate: A meta-analysis. PLoS ONE.

[51] Geersing T.H., Franssen E.J.F., Spronk P.E., van Kan H.J.M., den Reijer M., van der Voort P.H.J. (2021). Nephrotoxicity of continuous amphotericin B in critically ill patients with abdominal sepsis: A retrospective analysis with propensity score matching. J. Antimicrob. Chemother..

[52] Olivero J.J., Lozano-Mendez J., Ghafary E.M., Eknoyan G., Suki W.N. (1975). Mitigation of amphotericin B nephrotoxicity by mannitol. Br. Med. J..

[53] Bullock W.E., Luke R.G., Nuttall C.E., Bhathena D. (1976). Can mannitol reduce amphotericin B nephrotoxicity? Double-blind study and description of a new vascular lesion in kidneys. Antimicrob. Agents. Chemother..

[54] Smith S.R., Galloway M.J., Reilly J.T., Davies J.M. (1988). Amiloride prevents amphotericin B related hypokalaemia in neutropenic patients. J. Clin. Pathol..

[55] Ural A.U., Avcu F., Cetin T., Beyan C., Kaptan K., Nazaroglu N.K., Yalcin A. (2002). Spironolactone: Is it a novel drug for the prevention of amphotericin B-related hypokalemia in cancer patients?. Eur. J. Clin. Pharmacol..

[56] Camp M.J., Wingard J.R., Gilmore C.E., Lin L.S., Dix S.P., Davidson T.G., Geller R.B. (1998). Efficacy of low-dose dopamine in preventing amphotericin B nephrotoxicity in bone marrow transplant patients and leukemia patients. Antimicrob. Agents Chemother..

[57] Karimzadeh I., Sepehr-Sobhani A., Khoshnoud M.J., Sagheb M.M., Vejdani R., Jalali A., Mahi-Birjand M. (2020). Comparison of intravenous sodium bicarbonate and sodium chloride combination versus intravenous sodium chloride hydration alone in reducing amphotericin B nephrotoxicity: A randomized clinical trial. Res. Pharm. Sci..

[58] Panahi-Shokouh M., Moghaddas A., Badri S., Jabalameli S., Momenzadeh M., Mehrzad V., Ashrafi F. (2020). Pentoxifylline in Prevention of Amphotericin B-induced Nephrotoxicity and Electrolyte Abnormalities. J. Res. Pharm. Pract..

[59] Karimzadeh I., Khalili H., Sagheb M.M., Farsaei S. (2015). A double-blinded, placebo-controlled, multicenter clinical trial of N-acetylcysteine for preventing amphotericin B-induced nephrotoxicity. Expert Opin. Drug. Metab. Toxicol..

[60] Cleary J.D., Lewis R.E., Zeind C.S., Carvalho M.G. (2023). Fungal Infections. Applied Therapeutics: The Clinical Use of Drugs.

[61] Carver P.L., Eschenauer G.A., DiPiro J.T., Yee G.C., Posey L.M., Haines S.T., Nolin T.D., Ellingrod V. (2023). Invasive Fungal Infections. Pharmacotherapy: A Pathophysiologic Approach.

[62] Londeree J., Winterberg P.D., Garro R., George R.P., Shin S., Liverman R., Serluco A., Romero R., Yildirim I. (2020). Brincidofovir for the treatment of human adenovirus infection in pediatric solid organ transplant recipients: A case series. Pediatr. Transplant..

[63] Heil E.L., Corbett A.H., Zeind C.S., Carvalho M.G. (2023). Opportunistic Infections in patients living with HIV. Applied Therapeutics: The Clinical Use of Drugs.

[64] Foscarnet: Drug Information. UpToDate 2025. https://www.uptodateonline.ir/contents/mobipreview.htm?28/56/29575?source=see_link.

[65] UCSF Medical Center: Foscarnet Dosing and Monitoring. https://idmp.ucsf.edu/sites/g/files/tkssra4251/f/wysiwyg/UCSF%20Medical%20Center_%20Foscarnet%20Dosing%20and%20Monitoring.pdf.

[66] Foscarnet (Foscavir^®^). https://globalrph.com/dilution/foscarnet-foscavir/.

[67] Tsuji B.T., Pogue J.M., Zavascki A.P., Paul M., Daikos G.L., Forrest A., Giacobbe D.R., Viscoli C., Giamarellou H., Karaiskos I. (2019). International Consensus Guidelines for the Optimal Use of the Polymyxins: Endorsed by the American College of Clinical Pharmacy (ACCP), European Society of Clinical Microbiology and Infectious Diseases (ESCMID), Infectious Diseases Society of America (IDSA), International Society for Anti-infective Pharmacology (ISAP), Society of Critical Care Medicine (SCCM), and Society of Infectious Diseases Pharmacists (SIDP). Pharmacotherapy.

[68] Giacobbe D.R., Karaiskos I., Bassetti M. (2022). How do we optimize the prescribing of intravenous polymyxins to increase their longevity and efficacy in critically ill patients?. Expert Opin. Pharmacother..

[69] Kelesidis T., Falagas M.E. (2015). The safety of polymyxin antibiotics. Expert Opin. Drug Saf..

[70] Côté J.M., Kane-Gill S.L., Murray P.T. (2022). A ray of hope in the discord: Is adding piperacillin-tazobactam to vancomycin truly more nephrotoxic?. Intensive Care Med..

[71] Blair M., Côté J.M., Cotter A., Lynch B., Redahan L., Murray P.T. (2021). Nephrotoxicity from Vancomycin Combined with Piperacillin-Tazobactam: A Comprehensive Review. Am. J. Nephrol..

[72] Drew R.H., Sakoulas G. (2025). Vancomycin: Parenteral Dosing, Monitoring, and Adverse Effects in Adults. Uptodate. https://www.uptodate.com/contents/vancomycin-parenteral-dosing-monitoring-and-adverse-effects-in-adults.

[73] He N., Su S., Ye Z., Du G., He B., Li D., Liu Y., Yang K., Zhang X., Zhang Y. (2020). Evidence-based Guideline for Therapeutic Drug Monitoring of Vancomycin: 2020 Update by the Division of Therapeutic Drug Monitoring, Chinese Pharmacological Society. Clin. Infect. Dis..

[74] Park H.Y., Kim B.Y., Song J.Y., Seo K.H., Lee S.H., Choi S., Rhew K. (2025). Effects of AUC-Based Vancomycin Therapeutic Drug Monitoring on AKI Incidence and Drug Utilization: A Propensity Score-Weighted Analysis. J. Clin. Med..

[75] Ruiz-Gaviria R., Norman S.J., Elgendi S.H., Chou J., Ramdeen S. (2025). Incidence of Acute Kidney Injury in Trough and AUC/MIC Vancomycin Dosing Strategies in a Large Tertiary Care Center: A Retrospective Cohort. J. Clin. Pharmacol..

[76] Zamoner W., Eid K.Z.C., de Almeida L.M.B., Pierri I.G., Santos A.D., Balbi A.L., Ponce D. (2022). The Serum Concentration of Vancomycin as a Diagnostic Predictor of Nephrotoxic Acute Kidney Injury in Critically Ill Patients. Antibiotics.

[77] Drew R.H. (2025). Dosing and Administration of Parenteral Aminoglycosides. Uptodate. https://www.uptodate.com/contents/dosing-and-administration-of-parenteral-aminoglycosides.

[78] Destache C.J. (2014). Aminoglycoside-induced nephrotoxicity--a focus on monitoring: A review of literature. J. Pharm. Pract..

[79] Oliveira J.F.P., Cipullo J.P., Burdmann E.A. (2006). Aminoglycoside nephrotoxicity. Braz. J. Cardiovasc. Surg..

[80] Tashiro M., Obata Y., Takazono T., Ota Y., Wakamura T., Shiozawa Y., Tsuyuki A., Miyazaki T., Nishino T., Izumikawa K. (2022). Association between fluid infusions and the recovery from acute kidney injury in patients administered liposomal amphotericin B: A nationwide observational study. Ren. Fail..

[81] Rodriguez M., Zachary K.C. (2025). Cidofovir: An Overview. UpToDate. https://www.uptodate.com/contents/cidofovir-an-overview.

[82] Ortiz A., Justo P., Sanz A., Melero R., Caramelo C., Guerrero M.F., Strutz F., Müller G., Barat A., Egido J. (2005). Tubular cell apoptosis and cidofovir-induced acute renal failure. Antivir. Ther..

[83] Caruso Brown A.E., Cohen M.N., Tong S., Braverman R.S., Rooney J.F., Giller R., Levin M.J. (2015). Pharmacokinetics and safety of intravenous cidofovir for life-threatening viral infections in pediatric hematopoietic stem cell transplant recipients. Antimicrob. Agents. Chemother..

[84] Vora S.B., Brothers A.W., Englund J.A. (2017). Renal Toxicity in Pediatric Patients Receiving Cidofovir for the Treatment of Adenovirus Infection. J. Pediatric. Infect. Dis. Soc..

[85] Stern A., Alonso C.D., Garcia-Vidal C., Cardozo C., Slavin M., Yong M.K., Ho S.A., Mehta Steinke S., Avery R.K., Koehler P. (2021). Safety and efficacy of intravenously administered cidofovir in adult haematopoietic cell transplant recipients: A retrospective multicentre cohort study. J. Antimicrob. Chemother..

[86] Leowattana W. (2019). Antiviral Drugs and Acute Kidney Injury (AKI). Infect. Disord. Drug Targets..

[87] Sharfuddin A.A., Weisbord S.D., Palevsky P.M., Molitoris B.A., Chertow G., Luyckx V., Marsden P., Skorecki K., Taal M., Alan Y. (2020). Acute Kidney Injury. Brenner and Rector’s the Kidney.

[88] Tang Z., Li T., Dai H., Feng C., Xie X., Peng F., Lan G., Yu S., Wang Y., Fang C. (2022). Drug-induced Fanconi syndrome in patients with kidney allograft transplantation. Front. Immunol..

[89] Hall A.M., Bass P., Unwin R.J. (2014). Drug-induced renal Fanconi syndrome. QJM.

[90] Taber D.J., Dupuis R.E., Pilch N.A., Szempruch K., Zeind C.S., Carvalho M.G. (2023). Kidney and liver transplantation. Applied Therapeutics: The Clinical Use of Drugs.

[91] Grimley M.S., Chemaly R.F., Englund J.A., Kurtzberg J., Chittick G., Brundage T.M., Bae A., Morrison M.E., Prasad V.K. (2017). Brincidofovir for Asymptomatic Adenovirus Viremia in Pediatric and Adult Allogeneic Hematopoietic Cell Transplant Recipients: A Randomized Placebo-Controlled Phase II Trial. Biol. Blood Marrow Transplant..

[92] Jacobsen T., Sifontis N. (2010). Drug interactions and toxicities associated with the antiviral management of cytomegalovirus infection. Am. J. Health Syst. Pharm..

[93] Beaufils H., Deray G., Katlama C., Dohin E., Henin D., Sazdovitch V., Jouanneau C. (1990). Foscarnet and crystals in glomerular capillary lumens. Lancet.

[94] Zavras P., Su Y., Fang J., Stern A., Gupta N., Tang Y., Raval A., Giralt S., Perales M.A., Jakubowski A.A. (2020). Impact of Preemptive Therapy for Cytomegalovirus on Toxicities after Allogeneic Hematopoietic Cell Transplantation in Clinical Practice: A Retrospective Single-Center Cohort Study. Biol. Blood Marrow Transplant..

[95] Avery R.K., Arav-Boger R., Marr K.A., Kraus E., Shoham S., Lees L., Trollinger B., Shah P., Ambinder R., Neofytos D. (2016). Outcomes in Transplant Recipients Treated With Foscarnet for Ganciclovir-Resistant or Refractory Cytomegalovirus Infection. Transplantation.

[96] Minces L.R., Nguyen M.H., Mitsani D., Shields R.K., Kwak E.J., Silveira F.P., Abdel-Massih R., Pilewski J.M., Crespo M.M., Bermudez C. (2014). Ganciclovir-resistant cytomegalovirus infections among lung transplant recipients are associated with poor outcomes despite treatment with foscarnet-containing regimens. Antimicrob. Agents Chemother..

[97] Reusser P., Einsele H., Lee J., Volin L., Rovira M., Engelhard D., Finke J., Cordonnier C., Link H., Ljungman P. (2002). Randomized multicenter trial of foscarnet versus ganciclovir for preemptive therapy of cytomegalovirus infection after allogeneic stem cell transplantation. Blood.

[98] Domingo W., Nguyen I.T., Johnsrud J.J., Brown J.W. (2021). Continuous-Infusion Foscarnet Facilitates Administration in Hematopoietic Stem Cell Transplantation Patients. Transplant. Cell. Ther..

[99] Frochot V., Bazin D., Letavernier E., Jouanneau C., Haymann J.P., Daudon M. (2016). Nephrotoxicity induced by drugs: The case of foscarnet and atazanavir—A SEM and μFTIR investigation. Comptes Rendus Chim..

[100] Deffert C., Stoermann C., Ernandez T., Nabergoj M., Chalandon Y., Jaeger P. (2020). Phosphonoformate Crystalluria, A Warning Signal of Foscarnet-Induced Kidney Injury. Kidney Int. Rep..

[101] Lee G.C., Burgess D.S., DiPiro J.T., Yee G.C., Posey L.M., Haines S.T., Nolin T.D., Ellingrod V. (2023). Antimicrobial regimen selection. Pharmacotherapy: A Pathophysiologic Approach.

[102] Bacigalupo A., Boyd A., Slipper J., Curtis J., Clissold S. (2012). Foscarnet in the management of cytomegalovirus infections in hematopoietic stem cell transplant patients. Expert Rev. Anti. Infect. Ther..

[103] Foster G.G., Grant M.J., Thomas S.M., Cameron B., Raiff D., Corbet K., Loitsch G., Ferreri C., Horwitz M. (2020). Treatment with Foscarnet after Allogeneic Hematopoietic Cell Transplant (Allo-HCT) Is Associated with Long-Term Loss of Renal Function. Biol. Blood Marrow Transplant..

[104] Perazella M.A. (2025). Crystalline-Induced Acute Kidney Injury. UpToDate. https://www.uptodate.com/contents/crystalline-induced-acute-kidney-injury.

[105] Refardt J. (2020). Diagnosis and differential diagnosis of diabetes insipidus: Update. Best Pract. Res. Clin. Endocrinol. Metab..

[106] Akalya K., Murali T.M., Vathsala A., Teo B.W., Low S., Dharmasegaran D., Koh L.P., Bonney G.K., Hong W.Z., Da Y. (2022). Elevated Urinary Tissue Inhibitor of Metalloproteinase-2 and Insulin-Like Growth Factor Binding Protein-7 Predict Drug-Induced Acute Kidney Injury. Curr. Drug Metab..

[107] Deray G., Martinez F., Katlama C., Levaltier B., Beaufils H., Danis M., Rozenheim M., Baumelou A., Dohin E., Gentilini M. (1989). Foscarnet nephrotoxicity: Mechanism, incidence and prevention. Am. J. Nephrol..

[108] Cheung T.W., Jayaweera D.T., Pearce D., Benson P., Nahass R., Olson C., Wool G.M. (2000). Safety of oral versus intravenous hydration during induction therapy with intravenous foscarnet in AIDS patients with cytomegalovirus infections. Int. J. STD AIDS.

[109] Brown J.M., Chang A., Lee-Lam F., Tan S.K., Shashidhar S. (2014). Tolerability of foscarnet as a continuous infusion for treatment of herpesvirus infections. Biol. Blood Marrow Transplant..

[110] Kaye K.S., Pogue J.M., Kaye D., Bennett J.E., Dolin R., Blaser M.J. (2020). Polymyxins (Polymyxin Band Colistin). Mandell, Douglas, and Bennett’s Principles and Practice of Infectious Diseases.

[111] Bassetti M., Peghin M., Vena A., Giacobbe D.R. (2019). Treatment of Infections Due to MDR Gram-Negative Bacteria. Front. Med..

[112] Avedissian S.N., Liu J., Rhodes N.J., Lee A., Pais G.M., Hauser A.R., Scheetz M.H. (2019). A Review of the Clinical Pharmacokinetics of Polymyxin B. Antibiotics.

[113] Jafari F., Elyasi S. (2021). Prevention of colistin induced nephrotoxicity: A review of preclinical and clinical data. Expert Rev. Clin. Pharmacol..

[114] Oliota A.F., Penteado S.T., Tonin F.S., Fernandez-Llimos F., Sanches A.C. (2019). Nephrotoxicity prevalence in patients treated with polymyxins: A systematic review with meta-analysis of observational studies. Diagn. Microbiol. Infect. Dis..

[115] Eljaaly K., Bidell M.R., Gandhi R.G., Alshehri S., Enani M.A., Al-Jedai A., Lee T.C. (2021). Colistin Nephrotoxicity: Meta-Analysis of Randomized Controlled Trials. Open Forum Infect. Dis..

[116] Sisay M., Hagos B., Edessa D., Tadiwos Y., Mekuria A.N. (2021). Polymyxin-induced nephrotoxicity and its predictors: A systematic review and meta-analysis of studies conducted using RIFLE criteria of acute kidney injury. Pharmacol. Res..

[117] Nakwan N., Chokephaibulkit K., Imberti R. (2019). The Use of Colistin for the Treatment of Multidrug-resistant Gram-negative Infections in Neonates and Infants: A Review of the Literature. Pediatr. Infect. Dis. J..

[118] Karageorgos S.A., Bassiri H., Siakallis G., Miligkos M., Tsioutis C. (2019). Intravenous colistin use for infections due to MDR Gram-negative bacilli in critically ill paediatric patients: A systematic review and meta-analysis. J. Antimicrob. Chemother..

[119] Aggarwal R., Dewan A. (2018). Comparison of nephrotoxicity of Colistin with Polymyxin B administered in currently recommended doses: A prospective study. Ann. Clin. Microbiol. Antimicrob..

[120] Ballı F.N., Ekinci P.B., Kurtaran M., Kara E., Dizman G.T., Sönmezer M.Ç., Hayran M., Demirkan K., Metan G. (2024). Battle of polymyxin induced nephrotoxicity: Polymyxin B versus colistin. Int. J. Antimicrob. Agents.

[121] Wang J.L., Xiang B.X., Song X.L., Que R.M., Zuo X.C., Xie Y.L. (2022). Prevalence of polymyxin-induced nephrotoxicity and its predictors in critically ill adult patients: A meta-analysis. World J. Clin. Cases.

[122] Wagenlehner F., Lucenteforte E., Pea F., Soriano A., Tavoschi L., Steele V.R., Henriksen A.S., Longshaw C., Manissero D., Pecini R. (2021). Systematic review on estimated rates of nephrotoxicity and neurotoxicity in patients treated with polymyxins. Clin. Microbiol. Infect..

[123] Ordooei Javan A., Shokouhi S., Sahraei Z. (2015). A review on colistin nephrotoxicity. Eur. J. Clin. Pharmacol..

[124] Feng J.Y., Lee Y.T., Pan S.W., Yang K.Y., Chen Y.M., Yen D.H., Li S.Y., Wang F.D. (2021). Comparison of colistin-induced nephrotoxicity between two different formulations of colistin in critically ill patients: A retrospective cohort study. Antimicrob. Resist. Infect. Control.

[125] Yu X.B., Zhang X.S., Wang Y.X., Wang Y.Z., Zhou H.M., Xu F.M., Yu J.H., Zhang L.W., Dai Y., Zhou Z.Y. (2022). Population Pharmacokinetics of Colistin Sulfate in Critically Ill Patients: Exposure and Clinical Efficacy. Front. Pharmacol..

[126] Ozel A.S., Ergönül Ö., Korten V. (2019). Colistin nephrotoxicity in critically ill patients after implementation of a new dosing strategy. J. Infect. Dev. Ctries..

[127] Nation R.L., Rigatto M.H.P., Falci D.R., Zavascki A.P. (2019). Polymyxin Acute Kidney Injury: Dosing and Other Strategies to Reduce Toxicity. Antibiotics.

[128] Gai Z., Samodelov S.L., Kullak-Ublick G.A., Visentin M. (2019). Molecular Mechanisms of Colistin-Induced Nephrotoxicity. Molecules.

[129] Beirne G.J., Hansing C.E., Octaviano G.N., Burns R.O. (1967). Acute renal failure caused by hypersensitivity to polymyxin B sulfate. JAMA.

[130] Kallel H., Hamida C.B., Ksibi H., Bahloul M., Hergafi L., Chaari A., Chelly H., Bouaziz M. (2005). Suspected acute interstitial nephritis induced by colistin. J. Nephrol..

[131] Zavascki A.P., Nation R.L. (2017). Nephrotoxicity of Polymyxins: Is There Any Difference between Colistimethate and Polymyxin B?. Antimicrob. Agents Chemother..

[132] Pogue J.M., Lee J., Marchaim D., Yee V., Zhao J.J., Chopra T., Lephart P., Kaye K.S. (2011). Incidence of and risk factors for colistin-associated nephrotoxicity in a large academic health system. Clin. Infect. Dis..

[133] Sorlí L., Luque S., Grau S., Berenguer N., Segura C., Montero M.M., Álvarez-Lerma F., Knobel H., Benito N., Horcajada J.P. (2013). Trough colistin plasma level is an independent risk factor for nephrotoxicity: A prospective observational cohort study. BMC Infect. Dis..

[134] Doshi N., Mount K., Murphy C. (2011). Nephrotoxicity associated with intravenous colistin in critically ill patients. Pharmacotherapy.

[135] Paul M., Daikos G.L., Durante-Mangoni E., Yahav D., Carmeli Y., Benattar Y.D., Skiada A., Andini R., Eliakim-Raz N., Nutman A. (2018). Colistin alone versus colistin plus meropenem for treatment of severe infections caused by carbapenem-resistant Gram-negative bacteria: An open-label, randomised controlled trial. Lancet Infect. Dis..

[136] Lodise T.P., Fan W., Griffith D.C., Dudley M.N., Sulham K.A. (2017). A Retrospective Cohort Analysis Shows that Coadministration of Minocycline with Colistin in Critically Ill Patients Is Associated with Reduced Frequency of Acute Renal Failure. Antimicrob. Agents Chemother..

[137] Heybeli C., Canaslan K., Oktan M.A., Yıldız S., Arda H.Ü., Çavdar C., Çelik A., Gökmen N., Cömert B. (2021). Acute kidney injury following colistin treatment in critically-ill patients: May glucocorticoids protect?. J. Chemother..

[138] Wang J., Niu H., Wang R., Cai Y. (2019). Safety and efficacy of colistin alone or in combination in adults with *Acinetobacter baumannii* infection: A systematic review and meta-analysis. Int. J. Antimicrob. Agents.

[139] Gu W.J., Wang F., Tang L., Bakker J., Liu J.C. (2014). Colistin for the treatment of ventilator-associated pneumonia caused by multidrug-resistant Gram-negative bacteria: A systematic review and meta-analysis. Int. J. Antimicrob. Agents.

[140] Chen Z., Chen Y., Fang Y., Wang X., Chen Y., Qi Q., Huang F., Xiao X. (2015). Meta-analysis of colistin for the treatment of *Acinetobacter baumannii* infection. Sci. Rep..

[141] Chang K., Wang H., Zhao J., Yang X., Wu B., Sun W., Huang M., Cheng Z., Chen H., Song Y. (2022). Risk factors for polymyxin B-associated acute kidney injury. Int. J. Infect. Dis..

[142] Kwon J.A., Lee J.E., Huh W., Peck K.R., Kim Y.G., Kim D.J., Oh H.Y. (2010). Predictors of acute kidney injury associated with intravenous colistin treatment. Int. J. Antimicrob. Agents.

[143] Deryke C.A., Crawford A.J., Uddin N., Wallace M.R. (2010). Colistin dosing and nephrotoxicity in a large community teaching hospital. Antimicrob. Agents Chemother..

[144] Justo J.A., Bosso J.A. (2015). Adverse reactions associated with systemic polymyxin therapy. Pharmacotherapy.

[145] Kim J., Lee K.H., Yoo S., Pai H. (2009). Clinical characteristics and risk factors of colistin-induced nephrotoxicity. Int. J. Antimicrob. Agents.

[146] Giacobbe D.R., di Masi A., Leboffe L., Del Bono V., Rossi M., Cappiello D., Coppo E., Marchese A., Casulli A., Signori A. (2018). Hypoalbuminemia as a predictor of acute kidney injury during colistin treatment. Sci. Rep..

[147] Özkarakaş H., Köse I., Zincircioğlu Ç., Ersan S., Ersan G., Şenoğlu N., Köse Ş., Erbay R.H. (2017). Risk factors for colistin-associated nephrotoxicity and mortality in critically ill patients. Turk. J. Med. Sci..

[148] Spapen H., Jacobs R., Van Gorp V., Troubleyn J., Honoré P.M. (2011). Renal and neurological side effects of colistin in critically ill patients. Ann. Intensive Care.

[149] Forrest A., Garonzik S.M., Thamlikitkul V., Giamarellos-Bourboulis E.J., Paterson D.L., Li J., Silveira F.P., Nation R.L. (2017). Pharmacokinetic/Toxicodynamic Analysis of Colistin-Associated Acute Kidney Injury in Critically Ill Patients. Antimicrob. Agents Chemother..

[150] Sanabria J., Garzón V., Pacheco T., Avila M.P., Garcia J.C., Jaimes D., Torres A., Bustos R.H., Escobar-Perez J., Abril D. (2021). Estimation of the Difference in Colistin Plasma Levels in Critically Ill Patients with Favorable or Unfavorable Clinical Outcomes. Pharmaceutics.

[151] Jeong Y.J., Gu N., Kwack W.G., Kang Y., Park S.Y., Yoon Y.S. (2021). Prospective observational study of the impact of plasma colistin levels in patients with carbapenem-resistant *Acinetobacter baumannii* pneumonia. J. Glob. Antimicrob. Resist..

[152] Yang J., Liu S., Lu J., Sun T., Wang P., Zhang X. (2022). An area under the concentration-time curve threshold as a predictor of efficacy and nephrotoxicity for individualizing polymyxin B dosing in patients with carbapenem-resistant gram-negative bacteria. Crit. Care.

[153] Shabani-Borujeni M., Farvadi F., Abolmaali S.S., Firouzabadi D., Rasaei N., Karimzadeh I. (2025). The frequency, risk factors, onset time, and outcome of acute kidney injury induced by vancomycin, colistin, and liposomal amphotericin B in hospitalized patients. J. Renal Inj. Prev..

[154] Ko H.j., Jeon M.h., Choo E.j., Lee E.j., Kim T.h., Jun J.B., Gil H.W. (2011). Early acute kidney injury is a risk factor that predicts mortality in patients treated with colistin. Nephron Clin. Pract..

[155] MacLaren G., Spelman D. (2025). Polymyxins: An Overview. UpToDate. https://www.uptodate.com/contents/polymyxins-an-overview.

[156] Falagas M.E., Rizos M., Bliziotis I.A., Rellos K., Kasiakou S.K., Michalopoulos A. (2005). Toxicity after prolonged (more than four weeks) administration of intravenous colistin. BMC Infect. Dis..

[157] Okusa M.D., Knicely D.H. (2025). Etiology and Diagnosis of Prerenal Disease and Acute Tubular Necrosis in Acute Kidney Injury in Adults. UpToDate. https://www.uptodate.com/contents/etiology-and-diagnosis-of-prerenal-disease-and-acute-tubular-necrosis-in-acute-kidney-injury-in-adults.

[158] Moledina D.G., Perazella M.A. (2017). Drug-Induced Acute Interstitial Nephritis. Clin. J. Am. Soc. Nephrol..

[159] Vazin A., Malek M., Karimzadeh I. (2020). Evaluation of colistin nephrotoxicity and urinary level of kidney injury molecule-1 in hospitalized adult ICU patients. J. Renal Inj. Prev..

[160] Ciftci A., Izdes S., Altintas N.D. (2018). Factors Determining Nephrotoxicity and Mortality in Critical Care Patients Receiving Colistin. J. Infect. Dev. Ctries..

[161] Desai R.J., Kazarov C.L., Wong A., Kane-Gill S.L. (2022). Kidney Damage and Stress Biomarkers for Early Identification of Drug-Induced Kidney Injury: A Systematic Review. Drug Saf..

[162] Babic J.T., Manchandani P., Ledesma K.R., Tam V.H. (2017). Evaluation of Urinary KIM-1 for Prediction of Polymyxin B-Induced Nephrotoxicity. Antimicrob. Agents Chemother..

[163] Park S.Y., Eom J.S., Lee J.S., Ju Y.S., Park J.Y. (2018). Neutrophil Gelatinase-associated Lipocalin as a Predictor of Acute Kidney Injury in Patients during Treatment with Colistimethate Sodium. Infect. Chemother..

[164] Thammathiwat T., Tiranathanagul K., Srisawat N., Susantitaphong P., Praditpornsilpa K., Eiam-Ong S. (2020). Clinical and subclinical acute kidney injury in multidrug-resistant septic patients treated with colistimethate sodium: Incidence and clinical outcomes. Nephrology.

[165] Lakota E.A., Landersdorfer C.B., Nation R.L., Li J., Kaye K.S., Rao G.G., Forrest A. (2018). Personalizing Polymyxin B Dosing Using an Adaptive Feedback Control Algorithm. Antimicrob. Agents Chemother..

[166] Abdelraouf K., Braggs K.H., Yin T., Truong L.D., Hu M., Tam V.H. (2012). Characterization of polymyxin B-induced nephrotoxicity: Implications for dosing regimen design. Antimicrob. Agents Chemother..

[167] Okoduwa A., Ahmed N., Guo Y., Scipione M.R., Papadopoulos J., Eiras D.P., Dubrovskaya Y. (2018). Nephrotoxicity Associated with Intravenous Polymyxin B Once- versus Twice-Daily Dosing Regimen. Antimicrob. Agents Chemother..

[168] Alvarado Reyes Y., Cruz R., Gonzalez J., Perez Y., Wolowich W.R. (2019). Incidence of Acute Kidney Injury in Intermittent Versus Continuous Infusion of Polymyxin B in Hospitalized Patients. Ann. Pharmacother..

[169] Khalili H., Shojaei L., Mohammadi M., Beigmohammadi M.T., Abdollahi A., Doomanlou M. (2018). Meropenem/colistin versus meropenem/ampicillin-sulbactam in the treatment of carbapenem-resistant pneumonia. J. Comp. Eff. Res..

[170] Zalts R., Neuberger A., Hussein K., Raz-Pasteur A., Geffen Y., Mashiach T., Finkelstein R. (2016). Treatment of Carbapenem-Resistant *Acinetobacter baumannii* Ventilator-Associated Pneumonia: Retrospective Comparison Between Intravenous Colistin and Intravenous Ampicillin-Sulbactam. Am. J. Ther..

[171] Bassetti M., Garau J. (2021). Current and future perspectives in the treatment of multidrug-resistant Gram-negative infections. J. Antimicrob. Chemother..

[172] Mirjalili M., Mirzaei E., Vazin A. (2022). Pharmacological agents for the prevention of colistin-induced nephrotoxicity. Eur. J. Med. Res..

[173] Bozkurt I., Sharma A., Esen S. (2017). Colistin-induced nephrotoxicity and the role of N-acetylcysteine: A retrospective cohort study. J. Infect. Dev. Ctries..

[174] Timuroğlu A., Muslu S., Menteş S., Ünver S. (2018). Effect of N-Acetyl Cysteine on acute kidney injury in patients with colistin used in intensive care; retrospective study. Acta Oncol. Turc..

[175] Mosayebi S., Soltani R., Shafiee F., Assarzadeh S., Hakamifard A. (2022). Evaluation of the Effectiveness of N-Acetylcysteine in the Prevention of Colistin-Induced Nephrotoxicity: A Randomized Controlled Clinical Trial. J. Res. Pharm. Pract..

[176] Sirijatuphat R., Limmahakhun S., Sirivatanauksorn V., Nation R.L., Li J., Thamlikitkul V. (2015). Preliminary clinical study of the effect of ascorbic acid on colistin-associated nephrotoxicity. Antimicrob. Agents Chemother..

[177] Dalfino L., Puntillo F., Ondok M.J., Mosca A., Monno R., Coppolecchia S., Spada M.L., Bruno F., Brienza N. (2015). Colistin-associated Acute Kidney Injury in Severely Ill Patients: A Step Toward a Better Renal Care? A Prospective Cohort Study. Clin. Infect. Dis..

[178] Samsami M., Shabani M., Hajiesmaeili M., Tavakoli-Ardakani M., Ardehali S.H., Fatemi A., Barati S., Moradi O., Sahraei Z. (2021). The effects of vitamin E on colistin-induced nephrotoxicity in treatment of drug-resistant gram-negative bacterial infections: A randomized clinical trial. J. Infect. Chemother..

[179] Shojaei S., Torabi M., Sistanizad M., Kouchek M., Miri M.M., Salarian S., Ansar P. (2023). Oral melatonin for colistin-induced nephrotoxicity reduction in intensive care unit: A randomized placebo controlled clinical trial. Nephro-Urol. Mon..

[180] Shields R.K., Paterson D.L., Tamma P.D. (2023). Navigating Available Treatment Options for Carbapenem-Resistant Acinetobacter baumannii-calcoaceticus Complex Infections. Clin. Infect. Dis..

[181] Falagas M.E., Kasiakou S.K. (2006). Toxicity of polymyxins: A systematic review of the evidence from old and recent studies. Crit. Care.

[182] Zasowski E.J., Blackford M., DiPiro J.T., Yee G.C., Posey L.M., Haines S.T., Nolin T.D., Ellingrod V. (2023). Lower respiratory tract infections. Pharmacotherapy: A Pathophysiologic Approach.

[183] Roberts J.A., Taccone F.S., Udy A.A., Vincent J.L., Jacobs F., Lipman J. (2011). Vancomycin dosing in critically ill patients: Robust methods for improved continuous-infusion regimens. Antimicrob. Agents Chemother..

[184] Kan W.C., Chen Y.C., Wu V.C., Shiao C.C. (2022). Vancomycin-Associated Acute Kidney Injury: A Narrative Review from Pathophysiology to Clinical Application. Int. J. Mol. Sci..

[185] Chotiprasitsakul D., Tamma P.D., Gadala A., Cosgrove S.E. (2018). The Role of Negative Methicillin-Resistant Staphylococcus aureus Nasal Surveillance Swabs in Predicting the Need for Empiric Vancomycin Therapy in Intensive Care Unit Patients. Infect. Control Hosp. Epidemiol..

[186] Filippone E.J., Kraft W.K., Farber J.L. (2017). The Nephrotoxicity of Vancomycin. Clin. Pharmacol. Ther..

[187] Arnaud F.C.S., Libório A.B. (2020). Attributable nephrotoxicity of vancomycin in critically ill patients: A marginal structural model study. J. Antimicrob. Chemother..

[188] Fuhrman D.Y., Kane-Gill S., Goldstein S.L., Priyanka P., Kellum J.A. (2018). Acute kidney injury epidemiology, risk factors, and outcomes in critically ill patients 16-25 years of age treated in an adult intensive care unit. Ann. Intensive Care.

[189] Almeida J.P., João P.R.D., Sylvestre L.C. (2020). Impact of the use of nephrotoxic drugs in critically ill pediatric patients. Rev. Bras. Ter. Intensiv..

[190] Hays W.B., Tillman E. (2020). Vancomycin-Associated Acute Kidney Injury in Critically Ill Adolescent and Young Adult Patients. J. Pharm. Pract..

[191] Sinha Ray A., Haikal A., Hammoud K.A., Yu A.S. (2016). Vancomycin and the Risk of AKI: A Systematic Review and Meta-Analysis. Clin. J. Am. Soc. Nephrol..

[192] Bailie G.R., Neal D. (1988). Vancomycin ototoxicity and nephrotoxicity. A review. Med. Toxicol. Advers. Drug Exp..

[193] Buckley M.S., Komerdelj I.A., D’Alessio P.A., Rangan P., Agarwal S.K., Tinta N.C., Martinez B.K., Ziadat D.S., Yerondopoulos M.J., Kobic E. (2022). Vancomycin with concomitant piperacillin/tazobactam vs. cefepime or meropenem associated acute kidney injury in the critically ill: A multicenter propensity score-matched study. J. Crit. Care.

[194] Tantranont N., Luque Y., Hsiao M., Haute C., Gaber L., Barrios R., Adrogue H.E., Niasse A., Truong L.D. (2021). Vancomycin-Associated Tubular Casts and Vancomycin Nephrotoxicity. Kidney Int. Rep..

[195] Bellos I., Pergialiotis V., Perrea D.N. (2022). Kidney biopsy findings in vancomycin-induced acute kidney injury: A pooled analysis. Int. Urol. Nephrol..

[196] Nachiappa Ganesh R., Edwards A., El Zaatari Z., Gaber L., Barrios R., Truong L.D. (2024). Vancomycin nephrotoxicity: A comprehensive clinico-pathological study. PLoS ONE.

[197] Elyasi S., Khalili H., Dashti-Khavidaki S., Mohammadpour A. (2012). Vancomycin-induced nephrotoxicity: Mechanism, incidence, risk factors and special populations. A literature review. Eur. J. Clin. Pharmacol..

[198] Kwiatkowska E., Domański L., Dziedziejko V., Kajdy A., Stefańska K., Kwiatkowski S. (2021). The Mechanism of Drug Nephrotoxicity and the Methods for Preventing Kidney Damage. Int. J. Mol. Sci..

[199] Kannan L., Raj R. (2022). Case Report: Vancomycin-Associated Tubulointerstitial Nephritis in Clinical Practice-Case Report and Review of Literature. Front. Med..

[200] Michail S., Vaiopoulos G., Nakopoulou L., Revenas C., Aroni K., Karam P., Stathakis C., Thosios T. (1998). Henoch-Schoenlein purpura and acute interstitial nephritis after intravenous vancomycin administration in a patient with a staphylococcal infection. Scand. J. Rheumatol..

[201] Luque Y., Louis K., Jouanneau C., Placier S., Esteve E., Bazin D., Rondeau E., Letavernier E., Wolfromm A., Gosset C. (2017). Vancomycin-Associated Cast Nephropathy. J. Am. Soc. Nephrol..

[202] Stokes M.B., Stevens J.S. (2021). Vancomycin-Associated Cast Nephropathy: Reality or Fantasy?. Kidney360.

[203] Van Driest S.L., McGregor T.L., Velez Edwards D.R., Saville B.R., Kitchner T.E., Hebbring S.J., Brilliant M., Jouni H., Kullo I.J., Creech C.B. (2015). Genome-Wide Association Study of Serum Creatinine Levels during Vancomycin Therapy. PLoS ONE.

[204] Luther M.K., Timbrook T.T., Caffrey A.R., Dosa D., Lodise T.P., LaPlante K.L. (2018). Vancomycin Plus Piperacillin-Tazobactam and Acute Kidney Injury in Adults: A Systematic Review and Meta-Analysis. Crit. Care Med..

[205] Bellos I., Karageorgiou V., Pergialiotis V., Perrea D.N. (2020). Acute kidney injury following the concurrent administration of antipseudomonal β-lactams and vancomycin: A network meta-analysis. Clin. Microbiol. Infect..

[206] Blears E.E., Morris J., Popp D., Lee J.O., Norbury W.B. (2022). Kidney Injury in Critically Ill Patients Treated with Vancomycin and Zosyn or an Alternative: A Systematic Review and Meta-Analysis. Surg. Infect. (Larchmt).

[207] Pan K., Li R., Li Y., Ding X., Li X., Lv Q. (2025). Vancomycin combined with piperacillin/tazobactam increases the risk of acute kidney injury compared with vancomycin plus other anti-pseudomonal beta-lactams: A systematic review and network meta-analysis. J. Antimicrob. Chemother..

[208] Aslan A.T., Akova M. (2022). Piperacillin-Tazobactam Plus Vancomycin-Associated Acute Kidney Injury in Adults: Can Teicoplanin or Other Antipseudomonal Beta-Lactams Be Remedies?. Healthcare.

[209] Miano T.A., Hennessy S., Yang W., Dunn T.G., Weisman A.R., Oniyide O., Agyekum R.S., Turner A.P., Ittner C.A.G., Anderson B.J. (2022). Association of vancomycin plus piperacillin-tazobactam with early changes in creatinine versus cystatin C in critically ill adults: A prospective cohort study. Intensive Care Med..

[210] Hou Y., Ren J., Li J., Jin X., Gao Y., Li R., Zhang J., Wang X., Li X., Wang G. (2021). Relationship Between Mean Vancomycin Trough Concentration and Mortality in Critically Ill Patients: A Multicenter Retrospective Study. Front. Pharmacol..

[211] Ghasemiyeh P., Vazin A., Zand F., Haem E., Karimzadeh I., Azadi A., Masjedi M., Sabetian G., Nikandish R., Mohammadi-Samani S. (2022). Pharmacokinetic assessment of vancomycin in critically ill patients and nephrotoxicity prediction using individualized pharmacokinetic parameters. Front. Pharmacol..

[212] Aljefri D.M., Avedissian S.N., Rhodes N.J., Postelnick M.J., Nguyen K., Scheetz M.H. (2019). Vancomycin Area Under the Curve and Acute Kidney Injury: A Meta-analysis. Clin. Infect. Dis..

[213] Wong S., Selby P.R., Reuter S.E. (2024). Determination of a vancomycin nephrotoxicity threshold and assessment of target attainment in hematology patients. Pharmacol. Res. Perspect..

[214] Tangvichitrerk P., Changpradub D., Hemapanpairoa J., Juntanawiwat P., Santimaleeworagun W. (2025). Impact of vancomycin area under the curve in early or later phase on efficacy and nephrotoxicity in patients with enterococcal bloodstream infections: A multicenter study. BMC Infect. Dis..

[215] Haseeb A., Alqurashi M.K., Althaqafi A.S., Alsharif J.M., Faidah H.S., Bushyah M., Alotaibi A.F., Elrggal M.E., Mahrous A.J., Abuhussain S.S.A. (2022). A Systematic Review on Clinical Safety and Efficacy of Vancomycin Loading Dose in Critically Ill Patients. Antibiotics.

[216] Mei H., Wang J., Che H., Wang R., Cai Y. (2019). The clinical efficacy and safety of vancomycin loading dose: A systematic review and meta-analysis. Medicine.

[217] Wong-Beringer A., Joo J., Tse E., Beringer P. (2011). Vancomycin-associated nephrotoxicity: A critical appraisal of risk with high-dose therapy. Int. J. Antimicrob. Agents.

[218] Awdishu L., Le A., Amato J., Jani V., Bal S., Mills R.H., Carrillo-Terrazas M., Gonzalez D.J., Tolwani A., Acharya A. (2021). Urinary Exosomes Identify Inflammatory Pathways in Vancomycin Associated Acute Kidney Injury. Int. J. Mol. Sci..

[219] Wang Y., Dai N., Wei W., Jiang C. (2021). Outcomes and Nephrotoxicity Associated with Vancomycin Treatment in Patients 80 Years and Older. Clin. Interv. Aging.

[220] Jorgensen S.C.J., Murray K.P., Lagnf A.M., Melvin S., Bhatia S., Shamim M.D., Smith J.R., Brade K.D., Simon S.P., Nagel J. (2020). A Multicenter Evaluation of Vancomycin-Associated Acute Kidney Injury in Hospitalized Patients with Acute Bacterial Skin and Skin Structure Infections. Infect. Dis. Ther..

[221] Cano E.L., Haque N.Z., Welch V.L., Cely C.M., Peyrani P., Scerpella E.G., Ford K.D., Zervos M.J., Ramirez J.A., Kett D.H. (2012). Improving Medicine through Pathway Assessment of Critical Therapy of Hospital-Acquired Pneumonia (IMPACT-HAP) Study Group. Incidence of nephrotoxicity and association with vancomycin use in intensive care unit patients with pneumonia: Retrospective analysis of the IMPACT-HAP Database. Clin. Ther..

[222] Kane-Gill S.L., Ostermann M., Shi J., Joyce E.L., Kellum J.A. (2019). Evaluating Renal Stress Using Pharmacokinetic Urinary Biomarker Data in Critically Ill Patients Receiving Vancomycin and/or Piperacillin-Tazobactam: A Secondary Analysis of the Multicenter Sapphire Study. Drug Saf..

[223] Pang H.M., Qin X.L., Liu T.T., Wei W.X., Cheng D.H., Lu H., Guo Q., Jing L. (2017). Urinary kidney injury molecule-1 and neutrophil gelatinase-associated lipocalin as early biomarkers for predicting vancomycin-associated acute kidney injury: A prospective study. Eur. Rev. Med. Pharmacol. Sci..

[224] Imai S., Yamada T., Kasashi K., Kobayashi M., Iseki K. (2017). Usefulness of a decision tree model for the analysis of adverse drug reactions: Evaluation of a risk prediction model of vancomycin-associated nephrotoxicity constructed using a data mining procedure. J. Eval. Clin. Pract..

[225] Imai S., Yamada T., Kasashi K., Niinuma Y., Kobayashi M., Iseki K. (2019). Construction of a risk prediction model of vancomycin-associated nephrotoxicity to be used at the time of initial therapeutic drug monitoring: A data mining analysis using a decision tree model. J. Eval. Clin. Pract..

[226] Miyai T., Imai S., Kashiwagi H., Sato Y., Kadomura S., Yoshida K., Yoshimura E., Teraya T., Tsujimoto T., Kawamoto Y. (2020). A Risk Prediction Flowchart of Vancomycin-Induced Acute Kidney Injury to Use When Starting Vancomycin Administration: A Multicenter Retrospective Study. Antibiotics.

[227] Bao P., Sun Y., Qiu P., Li X. (2024). Development and validation of a nomogram to predict the risk of vancomycin-related acute kidney injury in critical care patients. Front. Pharmacol..

[228] He N., Su S., Yan Y., Liu W., Zhai S. (2020). The Benefit of Individualized Vancomycin Dosing Via Pharmacokinetic Tools: A Systematic Review and Meta-analysis. Ann. Pharmacother..

[229] Rybak M.J., Le J., Lodise T.P., Levine D.P., Bradley J.S., Liu C., Mueller B.A., Pai M.P., Wong-Beringer A., Rotschafer J.C. (2020). Executive Summary: Therapeutic Monitoring of Vancomycin for Serious Methicillin-Resistant Staphylococcus aureus Infections: A Revised Consensus Guideline and Review of the American Society of Health-System Pharmacists, the Infectious Diseases Society of America, the Pediatric Infectious Diseases Society, and the Society of Infectious Diseases Pharmacists. Pharmacotherapy.

[230] Flannery A.H., Bissell B.D., Bastin M.T., Morris P.E., Neyra J.A. (2020). Continuous Versus Intermittent Infusion of Vancomycin and the Risk of Acute Kidney Injury in Critically Ill Adults: A Systematic Review and Meta-Analysis. Crit. Care Med..

[231] Elyasi S., Khalili H., Hatamkhani S., Dashti-Khavidaki S. (2013). Prevention of vancomycin induced nephrotoxicity: A review of preclinical data. Eur. J. Clin. Pharmacol..

[232] Rahmani H., Khalili H. (2022). Prevention of Vancomycin-Induced Nephrotoxicity; An Updated Review of Clinical and Preclinical Studies. Infect. Disord. Drug Targets.

[233] Bamgbola O. (2016). Review of vancomycin-induced renal toxicity: An update. Ther. Adv. Endocrinol. Metab..

[234] Akundi S., Lee Y.R., Perry G.K., Fike D.S., Mnjoyan S. (2015). Nephrotoxicity in Recipients of Vancomycin vs. Vancomycin with Vitamin C. Int. J. Med. Pharm..

[235] He J., Mao E., Xu W., Zhao B., Jing F., Bian X., Chen E. (2020). High dose vitamin C significantly reduces the nephrotoxicity of vancomycin in critically ill patients. Zhonghua Wei Zhong Bing Ji Jiu Yi Xue.

[236] Badri S., Soltani R., Sayadi M., Khorvash F., Meidani M., Taheri S. (2020). Effect of N-acetylcysteine against Vancomycin-Induced Nephrotoxicity: A Randomized Controlled Clinical Trial. Arch. Iran. Med..

[237] Soltani R., Khorvash F., Meidani M., Badri S., Alaei S., Taheri S. (2020). Vitamin E in the prevention of vancomycin-induced nephrotoxicity. Res. Pharm. Sci..

[238] Hong T.S., Briscese K., Yuan M., Deshpande K., Aleksunes L.M., Brunetti L. (2021). Renoprotective Effects of Melatonin against Vancomycin-Related Acute Kidney Injury in Hospitalized Patients: A Retrospective Cohort Study. Antimicrob. Agents Chemother..

[239] Karimian A., Karimzadeh I., Shafiekhani M., Heidari R., Masjedi F., Izadi F., Barshan-Tashnizi N., Kane-Gill S.L., Mahmoudi L. (2025). Protective effects of silymarin on preventing vancomycin nephrotoxicity in infectious patients: A randomized, double-blinded, placebo-controlled, pilot clinical trial. Naunyn-Schmiedeberg’s Arch. Pharmacol..

[240] (2011). Vancomycin-Induced Acute Interstitial Nephritis. J. Hosp. Med..

[241] Sawada A., Kawanishi K., Morikawa S., Nakano T., Kodama M., Mitobe M., Taneda S., Koike J., Ohara M., Nagashima Y. (2018). Biopsy-proven vancomycin-induced acute kidney injury: A case report and literature review. BMC Nephrol..

[242] Karimzadeh I., Strader M., Kane-Gill S.L., Murray P.T. (2023). Prevention and management of antibiotic associated acute kidney injury in critically ill patients: New insights. Curr. Opin. Crit. Care.

[243] Pagkalis S., Mantadakis E., Mavros M.N., Ammari C., Falagas M.E. (2011). Pharmacological considerations for the proper clinical use of aminoglycosides. Drugs.

[244] Leggett J.E., Bennett J.E., Dolin R., Blaser M.J. (2020). Aminoglycosides. Mandell, Douglas, and Bennett’s Principles and Practice of Infectious Diseases.

[245] Wargo K.A., Edwards J.D. (2014). Aminoglycoside-induced nephrotoxicity. J. Pharm. Pract..

[246] Duong A., Simard C., Wang Y.L., Williamson D., Marsot A. (2021). Aminoglycosides in the Intensive Care Unit: What Is New in Population PK Modeling?. Antibiotics.

[247] Oliveira J.F., Silva C.A., Barbieri C.D., Oliveira G.M., Zanetta D.M., Burdmann E.A. (2009). Prevalence and risk factors for aminoglycoside nephrotoxicity in intensive care units. Antimicrob. Agents Chemother..

[248] Molitoris B.A. Manifestations of and Risk Factors for Aminoglycoside Nephrotoxicity. Uptodate, 2025. https://www.uptodate.com/contents/manifestations-of-and-risk-factors-for-aminoglycoside-nephrotoxicity.

[249] Ehrmann S., Helms J., Joret A., Martin-Lefevre L., Quenot J.P., Herbrecht J.E., Benzekri-Lefevre D., Robert R., Desachy A., Bellec F. (2019). Nephrotoxic drug burden among 1001 critically ill patients: Impact on acute kidney injury. Ann. Intensive Care.

[250] Slater M.B., Gruneir A., Rochon P.A., Howard A.W., Koren G., Parshuram C.S. (2017). Identifying High-Risk Medications Associated with Acute Kidney Injury in Critically Ill Patients: A Pharmacoepidemiologic Evaluation. Paediatr. Drugs.

[251] Lopez-Novoa J.M., Quiros Y., Vicente L., Morales A.I., Lopez-Hernandez F.J. (2011). New insights into the mechanism of aminoglycoside nephrotoxicity: An integrative point of view. Kidney Int..

[252] Molitoris B.A. Pathogenesis and Prevention of Aminoglycoside Nephrotoxicity and Ototoxicity. Uptodate, 2025. https://www.uptodate.com/contents/pathogenesis-and-prevention-of-aminoglycoside-nephrotoxicity-and-ototoxicity.

[253] Buring J.E., Evans D.A., Mayrent S.L., Rosner B., Colton T., Hennekens C.H. (1988). Randomized trials of aminoglycoside antibiotics: Quantitative overview. Rev. Infect Dis..

[254] Duszynska W., Taccone F.S., Hurkacz M., Kowalska-Krochmal B., Wiela-Hojeńska A., Kübler A. (2013). Therapeutic drug monitoring of amikacin in septic patients. Crit. Care.

[255] Drusano G.L., Ambrose P.G., Bhavnani S.M., Bertino J.S., Nafziger A.N., Louie A. (2007). Back to the future: Using aminoglycosides again and how to dose them optimally. Clin. Infect. Dis..

[256] Alinejad S., Yousefichaijan P., Rezagholizamenjany M., Rafie Y., Kahbazi M., Arjmand A. (2018). Nephrotoxic effect of gentamicin and amikacin in neonates with infection. Nephro-Urol. Mon..

[257] Gerlach A.T., Stawicki S.P., Cook C.H., Murphy C. (2011). Risk factors for aminoglycoside-associated nephrotoxicity in surgical intensive care unit patients. Int. J. Crit. Illn. Inj. Sci..

[258] McWilliam S.J., Antoine D.J., Sabbisetti V., Turner M.A., Farragher T., Bonventre J.V., Park B.K., Smyth R.L., Pirmohamed M. (2012). Mechanism-based urinary biomarkers to identify the potential for aminoglycoside-induced nephrotoxicity in premature neonates: A proof-of-concept study. PLoS ONE.

[259] Shihana F., Barron M.L., Mohamed F., Seth D., Buckley N.A. (2021). MicroRNAs in toxic acute kidney injury: Systematic scoping review of the current status. Pharmacol. Res. Perspect..

[260] Klementa V., Petejova N., Horak P., Kurasova E., Zadrazil J. (2025). Acute kidney injury due to gentamicin nephrotoxicity and specific miRNAs as biomarkers. Biomed. Pap. Med. Fac. Univ. Palacky Olomouc Czech Repub..

[261] Saikumar J., Hoffmann D., Kim T.M., Gonzalez V.R., Zhang Q., Goering P.L., Brown R.P., Bijol V., Park P.J., Waikar S.S. (2012). Expression, circulation, and excretion profile of microRNA-21, -155, and -18a following acute kidney injury. Toxicol. Sci..

[262] Mensa J., Barberán J., Soriano A., Llinares P., Marco F., Cantón R., Bou G., González Del Castillo J., Maseda E., Azanza J.R. (2018). Antibiotic selection in the treatment of acute invasive infections by Pseudomonas aeruginosa: Guidelines by the Spanish Society of Chemotherapy. Rev. Española Quimioter..

[263] Karimzadeh I., Abdollahpour-Alitappeh M., Ghaffari S., Mahi-Birjand M., Barkhordari A., Alemzadeh E. (2024). Aminoglycosides: Single- or Multiple-daily Dosing? An Updated Qualitative Systematic Review of Randomized Trials on Toxicity and Efficacy. Curr. Mol. Med..

[264] McWilliam S.J., Antoine D.J., Smyth R.L., Pirmohamed M. (2017). Aminoglycoside-induced nephrotoxicity in children. Pediatr. Nephrol..

[265] Qin J.P., Huang H.B., Zhou H., Zhu Y., Xu Y., Du B. (2021). Amikacin nebulization for the adjunctive therapy of gram-negative pneumonia in mechanically ventilated patients: A systematic review and meta-analysis of randomized controlled trials. Sci. Rep..

[266] Velissaris D., Karamouzos V., Marangos M., Pierrakos C., Karanikolas M. (2014). Pharmacokinetic changes and dosing modification of aminoglycosides in critically ill obese patients: A literature review. J. Clin. Med. Res..

[267] Sutherland S.M., Chawla L.S., Kane-Gill S.L., Hsu R.K., Kramer A.A., Goldstein S.L., Kellum J.A., Ronco C., Bagshaw S.M. (2016). Utilizing electronic health records to predict acute kidney injury risk and outcomes: Workgroup statements from the 15th ADQI consensus conference. Can. J. Kidney Health Dis..

[268] Dong J., Feng T., Thapa-Chhetry B., Cho B.G., Shum T., Inwald D.P., Newth C.J.L., Vaidya V.U. (2021). Machine learning model for early prediction of acute kidney injury (AKI) in pediatric critical care. Crit. Care.

[269] Griffin B.R., Mudireddy A., Horne B.D., Chonchol M., Goldstein S.L., Goto M., Matheny M.E., Street W.N., Vaughan-Sarrazin M., Jalal D.I. (2024). Predicting Nephrotoxic Acute Kidney Injury in Hospitalized Adults: A Machine Learning Algorithm. Kidney Med..

[270] Vicente-Vicente L., Casanova A.G., Hernández-Sánchez M.T., Pescador M., López-Hernández F.J., Morales A.I. (2017). A systematic meta-analysis on the efficacy of pre-clinically tested nephroprotectants at preventing aminoglycoside nephrotoxicity. Toxicology.

[271] Mahi-Birjand M., Yaghoubi S., Abdollahpour-Alitappeh M., Keshtkaran Z., Bagheri N., Pirouzi A., Khatami M., Sineh Sepehr K., Peymani P., Karimzadeh I. (2020). Protective effects of pharmacological agents against aminoglycoside-induced nephrotoxicity: A systematic review. Expert Opin. Drug Saf..

[272] Vlasic-Matas J., Rumboldt Z., Karelovic D. (2000). Renoprotective role of nifedipine during gentamicin therapy: Randomized controlled trial. Croat. Med. J..

[273] Heydari B., Khalili H., Dashti-Khavidaki S., Beig-Mohammadi M.T., Mohammadi M. (2016). Atorvastatin for Prevention of Amikacin-induced Electrolytes Imbalances; a Randomized Clinical Trial. Iran. J. Pharm. Res..

[274] Heydari B., Khalili H., Beigmohammadi M.T., Abdollahi A., Karimzadeh I. (2017). Effects of atorvastatin on biomarkers of acute kidney injury in amikacin recipients: A pilot, randomized, placebo-controlled, clinical trial. J. Res. Med. Sci..

[275] McWilliam S.J., Rosala-Hallas A., Jones A.P., Shaw V., Greenhalf W., Jaki T., Smyth A.R., Smyth R.L., Pirmohamed M. (2020). A randomised controlled trial of rosuvastatin for the prevention of aminoglycoside-induced kidney toxicity in children with cystic fibrosis. Sci. Rep..

[276] Mahi-Birjand M., Karimzadeh I., Zarban A., Abdollahpour-Alitappeh M., Seyed Alireza Saadatjoo S.A., Ziaee M. (2020). Protective effects of silymarin on gentamicin-induced nephrotoxicity in infectious patients: A randomized double blinded placebo-controlled clinical trial. Pharm. Sci..

[277] Mousavinasab S.R., Akhoundi-Meybodi Z., Mahmoudi L., Karimzadeh I. (2021). A randomized double-blinded placebo-controlled clinical trial on protective effects of pentoxifylline on gentamicin nephrotoxicity in infectious patients. Clin. Exp. Nephrol..

[278] Bulman Z.P., Cirz R., Hildebrandt D., Kane T., Rosario Z., Wlasichuk K., Park M., Andrews L.D. (2020). Unraveling the Gentamicin Drug Product Complexity Reveals Variation in Microbiological Activities and Nephrotoxicity. Antimicrob. Agents Chemother..

[279] Jospe-Kaufman M., Siomin L., Fridman M. (2020). The relationship between the structure and toxicity of aminoglycoside antibiotics. Bioorg. Med. Chem. Lett..

[280] Tang H.J., Lai C.C. (2020). Plazomicin-associated Nephrotoxicity. Clin. Infect. Dis..

[281] Bera S., Mondal D. (2022). Antibacterial Efficacies of Nanostructured Aminoglycosides. ACS. Omega.

[282] Elfaky M.A., Thabit A.K., Sirwi A., Fahmy U.A., Bahabri R.M., Al-Awad E.A., Basaeed L.F. (2019). Development of a Novel Pharmaceutical Formula of Nanoparticle Lipid Carriers of Gentamicin/α-Tocopherol and In Vivo Assessment of the Antioxidant Protective Effect of α-Tocopherol in Gentamicin-Induced Nephrotoxicity. Antibiotics.

[283] Madbouly N., Ooda A., Nabil A., Nasser A., Ahmed E., Ali F., Mohamed F., Faried H., Badran M., Ahmed M. (2024). The renoprotective activity of amikacin-gamma-amino butyric acid-chitosan nanoparticles: A comparative study. Inflammopharmacology.

[284] Prayle A.P., Jain K., Touw D.J., Koch B.C., Knox A.J., Watson A., Smyth A.R. (2016). The pharmacokinetics and toxicity of morning vs. evening tobramycin dosing for pulmonary exacerbations of cystic fibrosis: A randomised comparison. J. Cyst. Fibros..

[285] Prins J.M., Weverling G.J., van Ketel R.J., Speelman P. (1997). Circadian variations in serum levels and the renal toxicity of aminoglycosides in patients. Clin. Pharmacol. Ther..

